# Prevention of contrast-induced acute kidney injury in patients undergoing cardiovascular procedures-a systematic review and network meta-analysis

**DOI:** 10.1371/journal.pone.0168726

**Published:** 2017-02-02

**Authors:** Eliano P. Navarese, Paul A. Gurbel, Felicita Andreotti, Michalina Marta Kołodziejczak, Suetonia C. Palmer, Sofia Dias, Antonino Buffon, Jacek Kubica, Mariusz Kowalewski, Tomasz Jadczyk, Michał Laskiewicz, Marek Jędrzejek, Maximillian Brockmeyer, Flavio Airoldi, Marinella Ruospo, Stefano De Servi, Wojciech Wojakowski, Christopher O’ Connor, Giovanni F. M. Strippoli

**Affiliations:** 1 Department of Cardiology, Multimedica IRCCS, Milan, Italy; 2 Systematic Investigation and Research on Interventions and Outcomes (SIRIO) MEDICINE research network, Milan, Italy; 3 Inova Heart and Vascular Institute, Fairfax, VA, United States of America; 4 Institute of Cardiology, Catholic University, Rome, Italy; 5 Collegium Medicum in Bydgoszcz, University of Nicolaus Copernicus, Toruń, Poland; 6 Department of Medicine, University of Otago Christchurch, Christchurch, New Zealand; 7 University of Bristol, School of Social and Community Medicine, Bristol, United Kingdom; 8 Department of Cardiology and Internal Medicine, Collegium Medicum, Nicolaus Copernicus University, Bydgoszcz, Poland; 9 Department of Cardiac Surgery, Dr Antoni Jurasz Memorial University Hospital, Bydgoszcz, Poland; 10 Department of Cardiology, Medical University of Silesia in Katowice, Katowice, Poland; 11 Department of Cardiology, University of Duesseldorf, Duesseldorf, Germany; 12 Diaverum Medical Scientific Office, Diaverum Sweden AB, Lund, Sweden; 13 Sydney School of Public Health, University of Sydney, Sydney, Australia; 14 Department of Emergency and Organ Transplantation, University of Bari, Bari, Italy; Icahn School of Medicine at Mount Sinai, UNITED STATES

## Abstract

**Background:**

Interventional diagnostic and therapeutic procedures requiring intravascular iodinated contrast steadily increase patient exposure to the risks of contrast-induced acute kidney injury (CIAKI), which is associated with death, nonfatal cardiovascular events, and prolonged hospitalization. The aim of this study was to investigate the efficacy of pharmacological and non-pharmacological treatments for CIAKI prevention in patients undergoing cardiovascular invasive procedures with iodinated contrast.

**Methods and findings:**

MEDLINE, Google Scholar, EMBASE and Cochrane databases as well as abstracts and presentations from major cardiovascular and nephrology meetings were searched, up to 22 April 2016. Eligible studies were randomized trials comparing strategies to prevent CIAKI (alone or in combination) when added to saline versus each other, saline, placebo, or no treatment in patients undergoing cardiovascular invasive procedures with administration of iodinated contrast. Two reviewers independently extracted trial-level data including number of patients, duration of follow-up, and outcomes. Eighteen strategies aimed at CIAKI prevention were identified. The primary outcome was the occurrence of CIAKI. Secondary outcomes were mortality, myocardial infarction, dialysis and heart failure. The data were pooled using network meta-analysis. Treatment estimates were calculated as odds ratios (ORs) with 95% credible intervals (CrI). 147 RCTs involving 33,463 patients were eligible. Saline plus N-acetylcysteine (OR 0.72, 95%CrI 0.57–0.88), ascorbic acid (0.59, 0.34–0.95), sodium bicarbonate plus N-acetylcysteine (0.59, 0.36–0.89), probucol (0.42, 0.15–0.91), methylxanthines (0.39, 0.20–0.66), statin (0.36, 0.21–0.59), device-guided matched hydration (0.35, 0.12–0.79), prostaglandins (0.26, 0.08–0.62) and trimetazidine (0.26, 0.09–0.59) were associated with lower odds of CIAKI compared to saline. Methylxanthines (0.12, 0.01–0.94) or left ventricular end-diastolic pressure-guided hydration (0.09, 0.01–0.59) were associated with lower mortality compared to saline.

**Conclusions:**

Currently recommended treatment with saline as the only measure to prevent CIAKI during cardiovascular procedures may not represent the optimal strategy. Vasodilators, when added to saline, may significantly reduce the odds of CIAKI following cardiovascular procedures.

## Introduction

Interventional diagnostic and therapeutic procedures requiring intravascular iodinated contrast are performed in millions of patients worldwide and are steadily increasing patient exposure to the risks of contrast-induced acute kidney injury (CIAKI)[1]. As CIAKI is associated with death, nonfatal cardiovascular events, and prolonged hospitalization[2], the adoption of optimal therapeutic strategies to prevent this complication offers an opportunity to reduce patient morbidity and mortality. International guidelines advocate hydration as the standard therapeutic strategy to minimise CIAKI, with lower levels of recommendation assigned to other preventive strategies, given limited evidence from individual studies[3–5]. Although there are numerous candidate therapies to prevent CIAKI, individual head-to-head trials and pairwise meta-analyses cannot simultaneously compare all the available treatments tested in the periprocedural period.

We therefore performed a network meta-analysis of pharmacological and non-pharmacological treatments assessed in randomised controlled trials (RCTs) for the prevention of CIAKI in patients undergoing cardiovascular invasive procedures with iodinated contrast.

## Methods

We registered the study protocol with PROSPERO (CRD42015016488) and conducted and reported this review using the Preferred Reporting Items for Systematic Reviews and Meta-analyses (PRISMA) extension statement for reporting systematic reviews incorporating network meta-analyses of health care interventions (S1 File)[6].

### Data source and search strategy

We searched electronic databases (MEDLINE, Cochrane Central Register of Controlled Trials (CENTRAL), Google Scholar and EMBASE) and the websites www.clinicaltrials.gov, www.clinicaltrialresults.org, as well as proceedings of major cardiovascular and nephrology societies (American Society of Nephrology, European Dialysis and Transplant Association, World Congress of Nephrology, European Society of Cardiology, American College of Cardiology, and American Heart Association), up to 22 April 2016. We used the following search terms: randomised controlled trial, contrast-induced acute kidney injury, contrast-induced nephropathy, acute renal failure, contrast renal failure, contrast nephropathy, acetylcysteine, N-acetylcysteine, aminophylline, ascorbic acid, vitamin C, atrial natriuretic peptide (ANP), brain natriuretic peptide (BNP), fenoldopam, furosemide, haemodialysis, haemofiltration, renal replacement therapy, iloprost, prostaglandin E1 (PGE-1), mannitol, nebivolol, probucol, matched hydration, RenalGuard®, saline, 0.9% NaCl, statin, HMG-CoA reductase inhibitor, sodium bicarbonate, theophylline, trimetazidine, and left ventricular end-diastolic pressure (LVEDP)-guided hydration. The MEDLINE search strategy is provided in Table A in S1 Appendix.

### Selection criteria

We screened the title and abstract of all retrieved records for eligibility according to the study protocol. We applied no restrictions on language or publication status during this assessment. We then reviewed in full text all citations that appeared relevant to determine inclusion in the systematic review. We included RCTs comparing strategies to prevent CIAKI (alone or in combination) when added to saline versus each other, saline, placebo, or no treatment in patients undergoing cardiovascular invasive procedures with administration of iodinated contrast. We excluded non-randomised studies, studies in the setting of computed tomography alone, studies in which the number of events for the clinical outcome of interest was not reported, studies in which the evidence on a treatment was limited to one RCT with fewer than 100 patients, studies testing early-stage investigational strategies, and studies comparing the same drug at different doses without other control groups.

### Data extraction, synthesis and quality assessment

Data were independently abstracted by three investigators (TJ, MJ and VG) on prespecified forms. Differences were resolved by consensus after discussion with a fourth investigator (MK). Study risk of bias was assessed using Cochrane Collaboration criteria[7].

### Study endpoints

The primary endpoint was CIAKI as defined in the protocol of the original RCT. If multiple definitions of CIAKI were reported, the trial’s primary endpoint definition was applied. As a prespecified analysis, CIAKI in subsets of patients with moderate or severe kidney disease at baseline was assessed. We included mortality, myocardial infarction, need for dialysis, and heart failure as secondary outcomes.

### Statistical analyses

Network meta-analyses compare different treatments within a connected analytical network[8], integrating data from direct and indirect treatment comparisons whilst maintaining the randomisation design[9]. We used a Bayesian hierarchical random-effects model, which is the most conservative and accounts for differences among trials[10,11]. Comparative treatment estimates were calculated as odds ratios (ORs) with 95% credible intervals (CrI). Results for which the 95% CrI of the OR did not include unity were considered significant and are presented in bold in the outcome tables.

Model fit was assessed by comparing the posterior mean of the residual deviance to the number of data points[12,13]. We investigated the extent of heterogeneity in each network by examining the magnitude of the common heterogeneity variance τau^2^ for the network[14]. We applied a standard 0.5 zero-cell correction when there were zero events in one arm of the trial. Studies in which all arms had zero events were excluded as not providing evidence of relative treatment effects.

The network meta-analysis approach assumes consistency, i.e., that one can learn about treatment A versus treatment B through a common comparator, treatment C. We assumed that, in principle, participants in studies fulfilling our inclusion criteria could be randomly allocated to any of the treatments being compared. We evaluated evidence of inconsistency in the network, defined as difference in treatment estimates derived from direct and indirect treatment comparisons, using the node splitting method and the corresponding Bayesian p- value, which determines the statistical agreement between direct and indirect evidence for each split node[15]. This was implemented using the gemtc R package[16].

We also explored potential sources of statistical heterogeneity and inconsistency through subgroup analyses and univariate random-effects meta-regression[7]. To this end, we stratified studies according to baseline estimated glomerular filtration rate (eGFR), as either 30–59 mL/min per 1.73 m^2^ (moderate chronic kidney disease [CKD]), or <30 mL/min per 1.73 m^2^ (severe CKD) according to KDOQI stages of CKD[17]. We additionally performed meta-regression analyses, considering baseline mean age and diabetes as effect modifiers on estimates for CIAKI.

Calculation of the probability that each treatment is the best was performed by counting the proportion of iterations in the Monte Carlo simulation at which each treatment had the most favourable outcome. We also estimated the relative ranking probability of each treatment and obtained the treatment hierarchy of competing interventions using rankograms. To obtain the absolute probabilities of events for all treatments, a reference study[18] was chosen to provide the probability of events for the reference treatment onto which the odds ratios were applied (on the log-odds scale) to obtain the absolute probabilities of events for all treatments[19]. These were then used to calculate the numbers needed to treat (NNT) or to harm (NNH) to prevent or cause one event for the compared treatments[13,20].

In additional prespecified sensitivity analyses we excluded studies in which <100 mL of contrast medium was administered for assumed low complexity of the procedure, or studies that did not define CIAKI as a ≥25% relative or ≥0.5 mg/dL (≥44 μmol/L) absolute increase in serum creatinine from baseline within 48–72 hours after contrast exposure. We explored potential publication bias by constructing funnel plots for the clinical outcomes in which the standard error of the log of the OR was plotted against the OR[7]. The network meta-analysis models used noninformative prior distributions for effect sizes (Normal(0,100^2^)) and between-studies standard deviation (Uniform(0,2)), which yield comparable results to those obtained by conventional statistical analysis. Models were estimated using Markov Chain Monte Carlo implemented in WinBUGS 1.4.3. Convergence was achieved at 20,000 iterations for all outcomes and lack of autocorrelation was checked and confirmed.

We conducted the analyses in WinBugs version 1.4.3 (MRC Biostatistics Unit, Cambridge, UK) and R version 3.1.4 (R Development Core Team, Vienna, Austria).

## Results

### Study selection and characteristics

**Fig 1:**
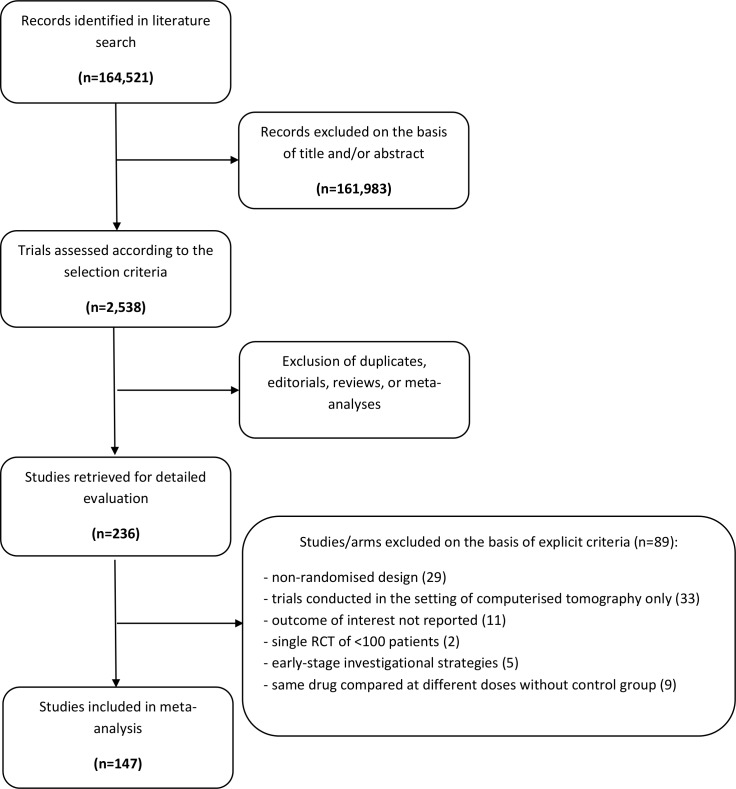
Flow diagram of network meta-analysis.

**Fig 2:**
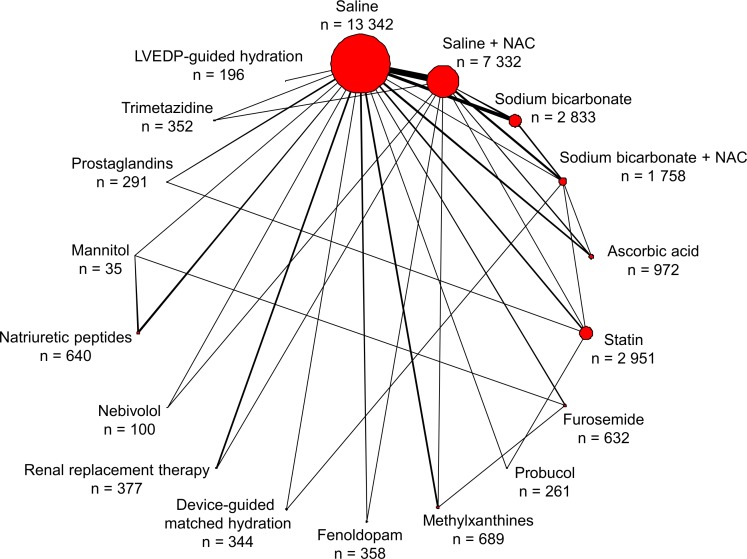
Network of treatment comparisons for prevention of contrast-induced acute kidney injury in patients undergoing cardiovascular invasive procedures. The size of the nodes corresponds to the number of trials that studied the treatments. Directly compared treatments are linked with a line, the thickness of which corresponds to the number of trials that assessed the comparison. LVEDP = left ventricular end-diastolic pressure; NAC = N-acetyl cysteine; n = number of patients allocated to a specific treatment.

A total of 33,463 patients from 147 studies were eligible for inclusion (Table B in S1 Appendix). The PRISMA flowchart showing the electronic search process is available as Fig 1. Eighteen treatments, including saline (reference treatment), saline plus N-acetylcysteine, sodium bicarbonate, sodium bicarbonate plus N-acetylcysteine, ascorbic acid, statins, furosemide, probucol, methylxanthines, fenoldopam, device-guided matched hydration, renal replacement therapy, nebivolol, natriuretic peptides, mannitol, prostaglandins, trimetazidine and LVEDP-guided hydration, were compared (Fig 2). The large majority of cardiovascular invasive procedures were coronary angiographies with or without percutaneous coronary intervention. The study mean age of participants was 65.0 years (Table C in S1 Appendix). On average, 142.80 mL of contrast agent per procedure was administered (Table D in S1 Appendix). The number of patients and events for single pairwise comparisons are shown in Table E in S1 Appendix.

### Risk of bias

The risk of bias in studies contributing to the primary outcome is shown in Fig A in S1 Appendix. The large majority of studies showed low risk of bias in random sequence generation and in incomplete outcome reporting. Several studies were open-label.

### Network consistency

There was no evidence of inconsistency between direct and indirect estimates for the compared drug treatments in CIAKI, with non-significant Bayesian p-values for the comparisons (Table F in S1 Appendix). Visual inspection of funnel plots did not suggest any small study effects (Figure B A-E in S1 Appendix). Evaluation of the goodness of fit of the models showed adequate fit for all outcomes (Table G in S1 Appendix). Heterogeneity among trials was moderate for CIAKI, mortality and heart failure, whereas it was substantial for need for dialysis and myocardial infarction (Table G in S1 Appendix).

### Primary endpoint: CIAKI

**Fig 3:**
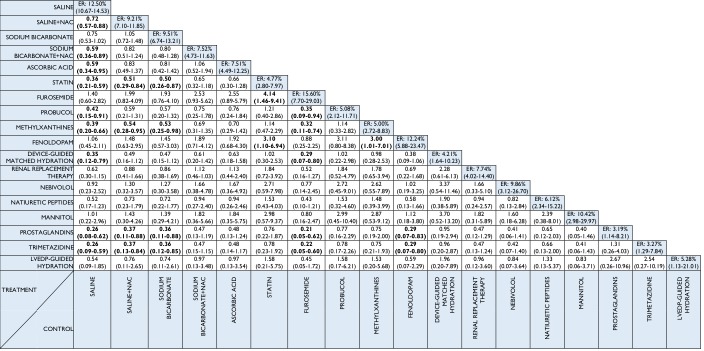
Network meta-analysis odds ratios and 95% credible intervals for contrast-induced acute kidney injury. Comparisons between treatments should be read from left to right. Estimates of treatment effects in the cell in common between the row-defining treatment and the column-defining treatment. Odds ratios lower than 1 favour the row-defining treatment. To obtain odds ratios for comparisons in the opposite direction, reciprocals should be taken. Significant results are in bold and underlined. ER = event rate; LVEDP = left ventricular end-diastolic pressure; NAC = N-acetylcysteine.

All 147 studies (n = 33,463) contributed to the network analysis for the primary endpoint of CIAKI (Fig 2). Several interventions were associated with lower odds of CIAKI compared with saline treatment (Fig 3). Saline plus N-acetyl cysteine (OR 0.72, 95% CrI 0.57–0.88), ascorbic acid (0.59, 0.34–0.95), sodium bicarbonate plus N-acetyl cysteine (0.59, 0.36–0.89), probucol (0.42, 0.15–0.91), methylxanthines (0.39, 0.20–0.66), statin (0.36, 0.21–0.59), device-guided matched hydration (0.35, 0.12–0.79), prostaglandins (0.26, 0.08–0.62) and trimetazidine (0.26, 0.09–0.59) were associated with lower odds of CIAKI compared to saline. Prostaglandin, trimetazidine, methylxanthine or statin therapy was associated with lower odds of CIAKI compared with saline plus N-acetyl cysteine. Table 1 summarises the probability that each treatment is the best compared to saline (reference treatment): there was a 31.03% probability that prostaglandins had the lowest CIAKI rate, followed by trimetazidine (27.38%) and LVEDP-guided hydration (14.37%). Rankograms for the competing treatments are shown in Fig C in S1 Appendix.

**Table 1:** Overall and GFR stratified analysis for CIAKI prevention, with NNT.

Treatment	Overall analysis		GFR 59–30 ml/min		GFR <30 ml/min	
	Probability to be the best [%]	NNT	Probability to be the best [%]	NNT	Probability to be the best [%]	NNT
Saline	-	-	-	-	-	-
Saline plus N-acetylcysteine	0.0	30.79	0.0	33.90	16.99	5.35
Sodium bicarbonate	0.0	32.61	0.0	35.74	na	na
Sodium bicarbonate plus N-acetyl cysteine	0.006	20.22	0.001	24.89	na	na
Ascorbic acid	0.03	19.97	0.004	25.63	na	na
Statin	1.53	13.07	1.14	14.57	na	na
Furosemide	0.0	-14.29	0.13	-5.35	0.73	-3.51
Probucol	5.24	13.48	na	na	na	na
Methylxanthines	2.17	13.48	3.66	14.10	na	na
Fenoldopam	0.01	14.81	0.02	-9.52	1.64	-2.31
Device-guided matched hydration	12.45	12.15	11.74	11.62	na	na
Renal replacement therapy	0.14	19.74	0.002	-5.61	62.40	4.72
Nebivolol	0.94	14.81	1.93	13.97	na	na
Natriuretic peptides	3.22	14.84	0.78	-3.93	18.24	5.49
Mannitol	1.13	13.52	8.22	10.71	na	na
Prostaglandins	31.03	10.87	35.63	10.24	na	na
Trimetazidine	27.38	10.97	33.24	10.43	na	na
LVEDP-guided hydration	14.37	12.21	3.51	13.43	na	na

CIAKI = contrast-induced acute kidney injury. GFR = glomerular filtration rate; na = not available; NNT = number needed to treat to prevent one episode of CIAKI.

### Secondary outcomes

**Fig 4:**
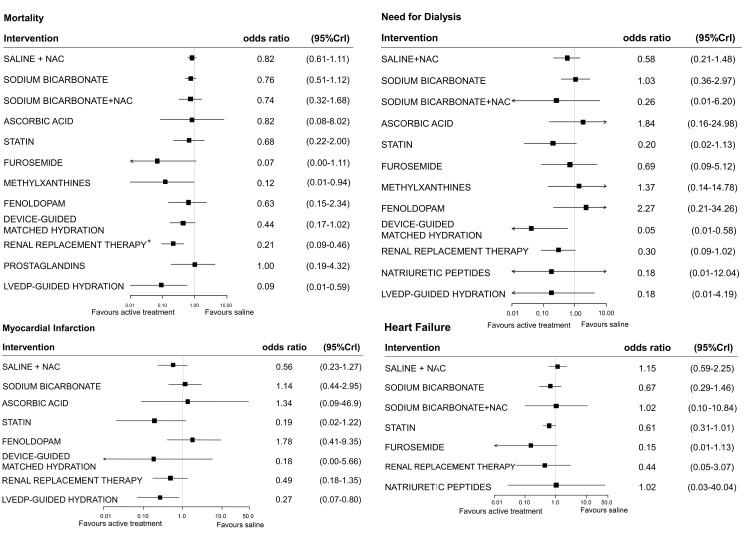
Forest plots for effect sizes of treatment strategies compared with saline, for mortality, dialysis, myocardial infarction and heart failure. Estimates are presented as odds ratios and 95% credible intervals (95% CrI). LVEDP = left ventricular end-diastolic pressure; NAC = N-acetylcysteine; *in severe chronic kidney disease.

Thirty-eight studies (involving 14,372 patients) contributed to the network for mortality (Fig D in S1 Appendix). Compared with saline, methylxanthines (0.12, 0.01–0.94) and LVEDP-guided hydration (0.09, 0.01–0.59) were associated with lower odds of death in the overall analysis (Fig 4, Table G in S1 Appendix). Data on the need for dialysis were derived from 43 studies (involving 14,985 patients). There were significantly lower odds of need for dialysis with device-guided matched hydration (0.05, 0.01–0.58). Myocardial infarction and heart failure data were available in 12 (n = 2,900) and 17 studies (n = 6,284), respectively. Among compared treatments, LVEDP-guided hydration was associated with lower odds of myocardial infarction compared to saline (0.27, 0.07–0.80). There was no evidence of significantly different effects on heart failure among treatments.

### Sensitivity and subgroup analyses

#### Kidney function

Sixty-nine studies (involving 12,934 patients) contributed to the analysis of CIAKI restricted to patients with moderate CKD. Among these patients, prostaglandins and trimetazidine were associated with lower odds of CIAKI when compared to either saline or saline plus N-acetyl-cysteine (Fig E in S1 Appendix); saline plus N-acetylcysteine was superior to saline alone. Renal replacement therapy was associated with increased odds of CIAKI when compared to saline plus N-acetylcysteine, statin, device-guided matched hydration, prostaglandins, trimetazidine and LVEDP-guided hydration. Nine studies (involving 1,205 patients) contributed to the analysis of CIAKI in patients with severe CKD: in these patients, renal replacement therapy and saline plus N-acetylcysteine were associated with lower odds of CIAKI when compared to saline (Fig F in S1 Appendix). Renal replacement therapy was associated with lower odds of death among severe CKD patients (0.21, 0.09–0.46) compared to saline (Fig 4).

#### Contrast volume

Among patients receiving >100 mL of contrast, the lowest odds of CIAKI were found with prostaglandins (0.26, 0.08–0.62), trimetazidine (0.26, 0.07–0.66), methylxanthines (0.27, 0.11–0.53) and device-guided matched hydration (0.33, 0.11–0.76) compared to saline (Fig G in S1 Appendix).

#### Stringent definition of CIAKI

In studies that defined CIAKI as a ≥25% relative, or ≥0.5 mg/dL or ≥44 μmol/L absolute, increase in serum creatinine from baseline within 48–72 hours after contrast exposure, the results were consistent with the main analyses (Fig H in S1 Appendix). The greatest CIAKI odds reductions were observed with trimetazidine (0.21, 0.05 to 0.75), prostaglandins (0.23, 0.09 to 0.56), probucol (0.27, 0.09 to 0.79) and statin (0.30, 0.16 to 0.52) as compared to saline. Similar results were observed when these treatments were compared to the combination of saline plus N-acetylcysteine.

#### Meta-regression

Meta-regression analysis showed that neither baseline prevalence of diabetes (beta: -0.25 [-0.61 to 0.10]) nor mean age (beta: -0.02 [-0.05 to 0.02]) influenced the overall results.

## Discussion

Cardiovascular invasive procedures with contrast media are increasingly performed in routine clinical practice. Contrast-induced acute kidney injury (CIAKI) is a leading cause of hospital-acquired renal failure, associated in turn with a stepwise increase of mortality and a relevant impact on public health[2]. The present report is the largest database ever analysed on different treatments to prevent CIAKI in the setting of cardiovascular invasive procedures.

The main findings of this network meta-analysis, including 147 RCTs and 33,463 patients, are that methylxanthines, prostaglandins and trimetazidine resulted in the lowest odds of CIAKI as compared to saline or to saline plus N-acetylcysteine following cardiovascular invasive procedures requiring iodinated contrast. When compared to saline alone, methylxanthines, renal replacement therapy limited to severe kidney disease patients, and LVEDP-guided hydration were associated with lower odds of mortality, while LVEDP-guided hydration was also associated with reduced odds of myocardial infarction.

European Society of Cardiology guidelines recommend hydration to prevent CIAKI in addition to limited contrast volume and to the use of iso- or low- osmolar contrast agents[5]. Similarly, the KDIGO Clinical Practice Guideline for Acute Kidney Injury recommends volume expansion with either isotonic sodium chloride or sodium bicarbonate in patients at high risk of CIAKI[4,21]. To date, however, no robust conclusions on the comparative efficacy of all potential preventive measures against CIAKI could be drawn, given the small numbers of patients enrolled in the single studies and the limited treatment group comparisons. As a consequence, preventive measures other than saline have been discouraged by international guidelines. The limited available trial evidence is reflected by variations in routine clinical practice. Current practice indicates that prevention of CIAKI with saline or saline plus N-acetylcysteine provides only limited efficacy, leaving the physician ultimately challenged by the choice of other treatment options[22].

A key finding of this network meta-analysis is the significantly lower odds of CIAKI with added-on vasodilator agents, namely methylxanthines, prostaglandins, and trimetazidine, compared to recommended standard treatments such as saline or saline plus N-acetylcysteine alone. This benefit persisted in the analyses stratified by baseline kidney function.

The pathophysiological basis for the present findings includes the reduction in renal perfusion and the toxic effect on tubular cells induced by contrast media that are generally recognised as important determinants of CIAKI[23]. This network meta-analysis challenges current international guidelines by demonstrating that effective prevention of CIAKI may not be optimally achieved with saline hydration alone. The addition of other agents, particularly vasodilators such as prostaglandins, methylxanthines and trimetazidine, has the potential to significantly reduce the occurrence of CIAKI and other patient-relevant endpoints, and warrants evaluation in large randomised trials. The complete biological mechanism underlying the effectiveness of vasodilator agents is not entirely clarified, and may include beneficial effects on cardiac function and haemodynamic performance.

In this network meta-analysis, the benefits of vasodilator agents assumed a particular distribution in patients with different baseline renal function. The protective effects of prostaglandins were evident in the main analysis and persisted in the sensitivity analyses of patients who had moderate CKD, studies in which a stringent CIAKI definition was used, and studies in which >100 mL contrast volume was administered. In response to ischaemia, the synthesis of prostaglandins is normally increased to modulate renal vascular resistance with a predominance of vasodilatory PGI2 and PGE2, in contrast to vasoconstricting PGF2 and thromboxane[24]. In the presence of CKD, the release of eicosanoids, predominantly PGE2, is impaired[25]. Therefore, it is biologically plausible that the external administration of prostaglandins might abrogate renal hypoxia that is indeed increased in more advanced stages of kidney dysfunction. The association of methylxanthines with lower odds of CIAKI, observed in the main analysis, was present but less pronounced in moderate stages of CKD. These results might be explained by the fact that methylxanthines affect renal haemodynamics by blocking compensatory adenosine-mediated vasoconstriction in the early stages of CKD [25,26]. This effect can be less pronounced in more advanced stages of renal dysfunction, owing to increased renal hypoxia and reduced synthesis of nitric oxide and adenosine.

Our findings that vasodilator agents can be beneficial are concordant with single pairwise meta-analyses showing that methylxanthines and trimetazidine reduce the incidence of CIAKI[27,28]. This large-scale network meta-analysis, however, substantially differs from the single pairwise meta-analyses by providing unified hierarchies of evidence for the several available treatments to prevent CIAKI. In this meta-analysis a significant benefit was observed with LVEDP-guided hydration on the clinical outcomes of death and myocardial infarction. Multiple reasons may explain this finding including plasma volume expansion, reduced renin activation, reduced loss of nitric oxide, reduced concentrations of reactive oxygen species, as well as higher dilution of contrast within the tubular lumen[29,30], although in our paper the magnitude of CIAKI reduction with this strategy was only numerical and not significant in comparison with other treatments, partly because the available evidence was limited to a single RCT.

Notably, in our network meta-analysis, renal replacement therapy exerted a possible GFR-dependent effect on CIAKI prevention compared to saline hydration, with signals of potential harm in patients with moderate CKD in contrast to lower odds of CIAKI among patients with severe CKD. The directionally opposite biological effects of renal replacement therapy in moderate and severe kidney dysfunction may have contributed at least in part to some heterogeneity in the network for the main CIAKI endpoint. The underlying mechanisms leading to harmful effects of renal replacement therapy are possibly multiple; hypotension and myocardial stunning may correlate with loss of residual renal function[31,32]; blood contact with the dialysis membrane may promote a pro-inflammatory systemic response which may in itself lead to accelerated kidney injury; as a result, renal replacement therapy should be considered only in severe CKD patients[33,34], who are most predisposed to develop CIAKI, and in whom the benefits of renal replacement therapy would be expected to outnumber the risks. Our current findings on the favorable effects of vasodilator treatment strategies are in line with results observed in recent network meta-analysis[35]. At variance with the previous meta-analysis focused on drug treatments only and with no inference on other outcomes than CIAKI [35], we extended the analysis to both drug and invasive treatments to prevent CIAKI as LVDEP-guided hydration that in our present paper has been proven to improve significantly survival. Moreover, in the paper by Su et al statin therapy was divided in high and low doses based on a non declared statin type and dose classification strategy, an operation that prompts caution when interpreting results on high dose statin therapy referred as a potential best strategyOur network meta-analysis has limitations that need to be considered when interpreting its findings. The results were analysed on trial level data and not on individual patient data. Not all trials assessed or reported GFR or CIAKI by the same method or definition; however, sensitivity analyses for different stages of CKD and using a stringent definition of CIAKI indicated stable results. Moderate to significant heterogeneity was found in the networks for most endpoints. A certain degree of heterogeneity is inevitable when integrating the large body of available RCT evidence on CIAKI. We explored potential sources of heterogeneity and found no evidence in meta-regression analyses that prespecified study-level variables such as age and prevalence of diabetes impacted the results. We also noted no statistically significant differences in treatment estimates from direct and indirect drug treatment comparisons, indicating that the assumption of transitivity, necessary for network analyses, was appropriate. Although the power of consistency checks is limited when direct comparisons are few, the non-significant Bayesian p-values describing the probability that the direct and indirect evidence differ, corroborate the a priori assumptions of transitivity. Some heterogeneity for the main CIAKI endpoint is likely explained by the directionally opposite treatment effect of renal replacement therapy in moderate versus severe CKD patients; however, other sources of unexplained heterogeneity cannot be excluded. Several studies presented design limitations including the small sample size and the fact that some of them were single center RCTs; however the present network meta-analysis by design addresses the small sample size in single studies by providing a large-scale analysis and more precise estimates. this network although is the largest source of evidence does not include several treatment agents such as sodium/potassium citrate, allopurinol for which however exists large uncertainty as measures to prevent CIAKI; a stratified analysis based on different statin doses was not performed given the substantial variations in doses and types used across different trials. Caution should be exerted when referring to the LVEDP-guided hydration findings given the single trial available so far. Given the association of LVEDP-guided therapy with lower CIAKI and mortality, further studies are needed to assess the LVEDP-guided strategy. Clinical endpoints other than CIAKI were analysed in a minority of studies and individual RCTs were underpowered for the assessment of mortality. Thus, these endpoint data should be viewed as preliminary, requiring formal testing in large RCTs. The potential harms and costs for the considered treatments were not explored.

## Conclusion

The results of the present network meta-analysis support the notion that treatment with saline during cardiovascular invasive procedures may not represent the optimal strategy to prevent CIAKI. Large specific RCTs aimed at enhancing CIAKI prevention are warranted, as the findings, if confirmed, would have a profound impact on public health.

## Supporting information

S1 Appendix Appendix. (DOC)

S1 File PRISMA Network Meta-analysis checklist. (DOCX)

## Abbreviations

CIAKI: contrast-induced acute kidney injury
CDK: chronic kidney disease
CrI: credible interval
GFR: glomerular filtration rate
LVEDP: left ventricular end-diastolic pressure
NAC: N-acetylcysteine
NMA: network meta-analysis
OR: odds ratio
RCT: randomised controlled trial
