# Supplementary material for: Prevention of contrast-induced acute kidney injury in patients undergoing cardiovascular procedures-a systematic review and network meta-analysis
Source: PLoS One. 2017 Feb 2;12(2):e0168726. doi: 10.1371/journal.pone.0168726 (PMC5289438; doi:10.1371/journal.pone.0168726)
Supplement: S1 Appendix — (DOC) [file pone.0168726.s001.doc]

Supporting File 1: Appendix

List of contents:

1. Tables:
   1. Appendix Table A. Full electronic search in MEDLINE database (up to 22 December 2015).
   2. Appendix Table B. Study characteristics. Contrast induced acute kidney injury definitions.
   3. Appendix Table C. Patient characteristics.
   4. Appendix Table D. Procedural characteristics.
   5. Appendix Table E. Data synthesis for pairwise comparisons of primary and secondary outcomes.
   6. Appendix Table F. Difference in treatment estimates from direct and indirect comparisons using node splitting method and corresponding test for inconsistency.
   7. Appendix Table G. Between-trial heterogeneity and evaluation of model fit.
   8. Appendix Table H. Event rates for secondary clinical outcomes.
2. Figures:
   1. Appendix Figure A. Risk of bias of studies included in the network meta-analysis.
   2. Appendix Figure B. Funnel plots of randomised controlled trials included in the network meta-analysisfor risk of: CIAKI (A), mortality (B), myocardial infarction (C), need for dialysis (D), heart failure (E).
   3. Appendix Figure C. Rankograms of strategies to prevent contrast-induced acute kidney injury.
   4. Appendix Figure D. Network of treatments for secondary clinical outcomes.
   5. Appendix Figure E. Pooled odds ratio and 95% credible intervals determined by network meta-analysis for contrast-induced acute kidney injury in patients with moderate chronic kidney disease.
   6. Appendix Figure F. Pooled odds ratio and 95% credible intervals determined by network meta-analysis for contrast-induced acute kidney injury in patients with severe chronic kidney disease.
   7. Appendix Figure G. Pooled odds ratio and 95% credible intervals determined by network meta-analysis for contrast-induced acute kidney injury in patients receiving >100 ml of contrast medium.
   8. Appendix Figure H. Pooled odds ratio and 95% credible intervals determined by network meta-analysis for contrast-induced acute kidney injury defined according to a relative (>25%) or absolute (>0.5 mg/dL or >44 umol/L) increase in serum creatinine from baseline within 48-72 hours.
3. Guideline summaries:
   1. Summary of current 2014 European Society of Cardiology guideline recommendations on CIAKI prevention.
   2. Summary of current 2011 ACCF/AHA/SCAI Guideline for Percutaneous Coronary Intervention recommendations on CIAKI prevention.
   3. Summary of current 2012 KDIGO Clinical Practice Guideline for Acute Kidney Injury recommendations on CIAKI prevention.
   4. Summary of 2014 KHA-CARI guideline recommendations on CIAKI prevention.
4. Appendix references

**Appendix Table A. Full electronic search in MEDLINE database (up to 22 December 2015)**.

| **Search** | **Query** | **Items found** |
| --- | --- | --- |
| 1 | Search contrast-induced acute kidney injury | 745 |
| 2 | Search contrast-induced acute kidney injury AND randomised trial | 159 |
| 3 | Search contrast-induced nephropathy | 1657 |
| 4 | Search contrast-induced nephropathy AND randomised trial | 346 |
| 5 | Search acute renal failure | 63267 |
| 6 | Search acute renal failure AND randomised trial | 2188 |
| 7 | Search contrast renal failure | 9141 |
| 8 | Search contrast renal failure AND randomised trial | 689 |
| 9 | Search contrast nephropathy | 21761 |
| 10 | Search contrast nephropathy AND randomised trial | 987 |
| 11 | Search saline AND contrast | 17085 |
| 12 | Search 0.9% NaCl AND contrast | 409 |
| 13 | Search acetyl cysteine AND contrast | 1498 |
| 14 | Search N-acetyl cysteine AND contrast | 1498 |
| 15 | Search sodium bicarbonate AND contrast | 839 |
| 16 | Search probucol AND contrast | 114 |
| 17 | Search statin AND contrast | 1536 |
| 18 | Search HMG-CoA reductase inhibitor AND contrast | 1382 |
| 19 | Search ascorbic acid AND contrast | 1919 |
| 20 | Search vitamin C AND contrast | 2143 |
| 21 | Search aminophylline AND contrast | 192 |
| 22 | Search atrial natriuretic peptide AND contrast | 1683 |
| 23 | Search brain natriuretic peptide AND contrast | 724 |
| 24 | Search fenoldopam AND contrast | 166 |
| 25 | Search furosemide AND contrast | 971 |
| 26 | Search haemodialysis AND contrast | 3966 |
| 27 | Search haemofiltration AND contrast | 295 |
| 28 | Search renal replacement therapy AND contrast | 5453 |
| 29 | Search iloprost AND contrast | 245 |
| 30 | Search prostaglandin E1 AND contrast | 786 |
| 31 | Search PGE-1 AND contrast | 62 |
| 32 | Search mannitol AND contrast | 1595 |
| 33 | Search nebivolol AND contrast | 57 |
| 34 | Search matched hydration AND contrast | 32 |
| 35 | Search RenalGuard AND contrast | 9 |
| 36 | Search theophylline AND contrast | 2032 |
| 37 | Search trimetazidine AND contrast | 28 |
| 38 | Search left ventricular end-diastolic pressure guided hydration AND contrast | 29 |

**Appendix Table B. Study characteristics. Contrast-induced acute kidney i**njury definitions.

| **Study** | **Year** | **ITT** | **Intervention** | **Control** | **CIAKI definition** | **CIAKI timepoint (h)** | **Follow-up** |
| --- | --- | --- | --- | --- | --- | --- | --- |
| **PRATO-ACS [Leoncini M. et al][1]** | 2014 | 543 | - Rosuvastatin 40 mg p.o. on admission, followed by 20 mg/day. Following discharge, 10 mg/day for eGFR <30 ml/min/m². | No statin treatment during hospitalisation; 40 mg/day atorvastatin at discharge | Increase in SCr of ≥ 0.5 mg/dl or ≥ 25% (within 48-72h)  or  Increase in SCr of ≥ 0.3 mg/dl within 48h-72h  or  Decrease in eGFR ≥ 25% within 72h | 48 to 72 | In-hospital  30 days 6 months |
| **MYTHOS [Marenzi G] [2]** | 2012 | 174 | - Furosemide 0.5 mg/kg i.v. (max. 50 mg) 30 min after 250 mL of saline i.v.  - procedure was started after urine flow >300 mL/h  - additional furosemide up to 2.0 mg/kg was applied when urine output <300 mL/h during procedure (21% of cases)  - urine flow matched hydration with i.v. isotonic saline continued until 4h post procedure | Isotonic saline i.v. (1 mL/kg/h; 0.5 mL/kg/h for patients with LVEF <40%) for 12h before and after procedure | Increase in SCr of ≥ 0.5 mg/dl  or  ≥ 25% | 72 | In-hospital |
| **Shaikh F et al. [3]** | 2007 | 320 | - NAC 1200 mg p.o. (2-12h before and 6-12h after procedure) with sodium bicarbonate 154 mEq/L i.v. (3mL/kg/h for 1h before and 1mL/kg/h for 6h after procedure) | NAC 1200 mg p.o. (2-12h before and 6-12h after procedure) with Saline 154 mEq/L (3 mL/kg/h for 1h before and 1 mL/kg/h for 6h after procedure) | Increase in SCr of ≥ 0.5 mg/dL  or  ≥ 25% | 48 | In-hospital |
| **CINSTEMI [Thayssen P et al][4]** | 2014 | 715 | 1) NAC 1200 mg p.o. before procedure and daily for 48h  or  2) isotonic sodium bicarbonate (167 mmol/L), 500 mL in the first hour followed by 100 mL/h for 5h  or  3) NAC 1200 mg p.o. and isotonic NaHCO_3_ i.v. as in 1) + 2)  all three with isotonic saline ≥60 mL/h i.v. for a minimum of 6h | Isotonic saline i.v. (≥ 60 mL/h i.v. for a minimum of 6h) | Increase in SCr of ≥25% | 48 to 72 | In-hospital  30 days |
| **Ozcan et al.[5]** | 2007 | 264 | - sodium bicarbonate 154 mEq/L i.v. (in 5% dextrose in water), 1 mL/kg/h, max. 100 mL/h, for 6h before and after procedure  - NAC 600 mg p.o. (b.i.d. starting the day before and continued through the day of procedure) and 154 mEq/L saline i.v. (1 mL/kg/h, max. 100 mL/h, for 6h before and after procedure) | 154 mEq/L saline i.v. (1 mL/kg/h, max 100 mL/h) for 6h before and after procedure | Increase in SCr of > 0.5 mg/dL  or  > 25% (within 48h after procedure) | 48 | In-hospital |
| **Castini et al.[6]** | 2010 | 156 | - NAC 600 mg p.o (b.i.d. the day before and the day of procedure), with isotonic saline i.v. (1 mL/kg/h for 12h before and after procedure)  - Sodium bicarbonate 154 mEq/L i.v. (3 mL/kg for 1h before procedure, continued with 1 mL/kg/h during and after procedure for 6h | Isotonic saline i.v. (1 mL/kg/h for 12h before and after procedure) | Increase in SCr of ≥ 0.5 mg/dL  or  ≥ 25% (within up to 120h after procedure) | 24  48  120 | In-hospital |
| **Brueck et al.[7]** | 2013 | 520 | - NAC 600 mg i.v. (in 250 mL isotonic saline over 30min, 24h and 1h before procedure)  - Ascorbic acid 500 mg i.v. (in 250 mL isotonic saline over 30min, 24h and 1h before procedure) | - Placebo (250 mL 250 mL isotonic saline over 30min, 24h and 1h before procedure) | Increase in SCr of ≥ 0.5 mg/dL (within 72h after procedure) | 72 | In-hospital |
| **Albabtain et al.[8]** | 2013 | 185 | - NAC 600 mg p.o. (b.i.d. for 2 days starting the evening before procedure)  - Ascorbic acid p.o. (3 g 2h before procedure, 2 g immediately after and 2 g 24h after procedure) | Placebo p.o. | Increase in SCr of ≥ 0.5 mg/dL  or  decrease in CCr of  ≥ 25 % | 96 to 120 | In-hospital |
| **Kinbara et al.[9]** | 2010 | 45 | - NAC 704 mg p.o. (b.i.d. day before and the day of procedure)  - Aminophylline 250 mg i.v. (30 min before procedure in 100 ml isotonic saline)  both with isotonic saline i.v. (1 mL/kg/h for 30 min before and 10h after procedure) | Isotonic saline i.v. (1 mL/kg/h for 30 min before and 10h after procedure) | Increase in SCr of > 0.5 mg/dL | 48 | In-hospital |
| **Baskurt et al.[10]** | 2009 | 217 | - NAC 600 mg p.o. (b.i.d. day before and the day of procedure)  - NAC 600 mg p.o. and Theophylline 200 mg p.o. (b.i.d. day before and the day of procedure)  both with isotonic saline i.v. (1 mL/kg/h for 12h before and after procedure) | Isotonic saline i.v. (1 mL/kg/h for 12h before and after procedure) | Increase in SCr of > 0.5 mg/dL | 48 | In-hospital |
| **Allaqaband et al.[11]** | 2002 | 123 | - NAC 600 mg p.o. (b.i.d. day before and the day of procedure)  - Fenoldopam 0.1 µg/kg/min i.v. (4h before and after procedure)  both with 0.45% saline i.v. (1 mL/kg/h for 12h before and after the procedure) | Saline 0.45% i.v. (1 mL/kg/h for 12h before and after procedure) | Increase in SCr of ≥ 0.5 mg/dL | 48 | In-hospital |
| **Loutrianakis et al.[12]** | 2003 | 68 | - NAC 600 mg p.o. (b.i.d. day before and on the day of procedure)  - Fenoldopam 0.1 µg/kg/min i.v. (starting 30 min before and for 4h after procedure)  both with 0.45% saline i.v. (1 mL/kg/h before and after the procedure) | Saline 0.45% i.v. (1 mL/kg/h for 12h before and after procedure) | Increase in SCr of > 0.5 mg/dL  or  > 25% from baseline | 120 to 168 | In-hospital |
| **Reinecke et al.[13]** | 2007 | 424 | - single hemodialysis (within 20 min after procedure)  - NAC 600mg p.o. (2 doses before and 2 doses after procedure)  both with Glucose 5% 500 mL i.v. with isotonic saline 500 mL i.v. (over 12h before, and again for 12h after procedure) | Glucose 5% 500 mL i.v. with isotonic saline 500 mL i.v. (over 12h before and again for 12h after procedure) | Increase in SCr of > 0.5 mg/dL within 48-72h after procedure | 24h, 72h,  and  30 to 60 days | Long term 63d-1316d with median of 553d |
| **Gunebakmaz et al.[14]** | 2012 | 120 | - NAC 600 mg p.o. (b.i.d. 2 days before till 1 day after procedure)  - Nebivolol 5 mg p.o. (o.d. 2 days before till 1 day after procedure)  both with isotonic saline i.v. (1 mL/kg/h for 6h before and 12h after procedure) | Isotonic saline i.v. (1 mL/kg/h for 6h before and 12h after procedure) | Increase in SCr of ≥ 0.5 mg/dL  or  ≥ 25% | 48h and/or  120h | In-hospital |
| **ACT Investigators [15]** | 2011 | 2308 | - NAC 1200 mg p.o. (b.i.d. day before and after procedure) | 1200 mg Placebo p.o. (b.i.d. day before and after procedure) | Increase in SCr of ≥ 25%; increase in SCr >0.5 mg/dL between 48-96h | 48 to 96 | 30 days |
| **Miner et al.[16]** | 2004 | 180 | - NAC 2000 mg p.o. (3 or 2 doses depending on time of admission in relation to procedure) | Placebo p.o. (3 or 2 doses depending on time of admission in relation to procedure) | Increase in SCr of > 0.5 mg/dL | 48 to 72 | 6 months |
| **Goldenberg et al.[17]** | 2004 | 80 | - NAC 600 mg p.o. (t.i.d. for 48h, starting 24h before procedure) and Saline 0.45% (1 mL/kg/h for 12h before and 12h after procedure) | Saline 0.45% i.v. (1 mL/kg/h for 12h before and after procedure) | Increase in SCr of ≥ 0.5 mg/dL | 48 | 7 days |
| **Durham et al.[18]** | 2002 | 79 | - NAC 1200mg p.o. (1h before and 3h after procedure) | Placebo p.o. (1h before and 3h after procedure) | Increase in SCr of > 0.5 mg/dL | 48 | 6 days |
| **Oldemeyer et al.[19]** | 2003 | 96 | - NAC 1500 mg p.o. (before angiography and every 12h for 4 doses) | Placebo p.o. (before angiography and every 12h for 4 doses) | Increase in SCr of ≥ 0.5 mg/dL  or  ≥ 25% | 24 or 48 | In-hospital |
| **Baker et al. (RAPPID trial)[20]** | 2003 | 80 | - NAC 150 mg/kg i.v. (in 500 mL isotonic saline, over 30 min before procedure) followed by NAC 50 mg/kg (in 500 mL isotonic saline over 4h) | Isotonic saline i.v. (1 mL/kg/h for 12h before and after procedure | Increase in SCr of > 25% | 48 or 96 | In-hospital |
| **Kefer et al.[21]** | 2003 | 104 | - NAC 1200 mg i.v (in 200 mL of isotonic saline; once 12h before and again after procedure) | Placebo i.v. (in 200 mL of isotonic saline; once 12h before and again after procedure) | Increase in SCr of > 0.5 mg/dL  or  > 25% | 24 | In-hospital |
| **Efrati et al.[22]** | 2003 | 55 | - NAC 1 g p.o. (b.i.d. 24h before and 24h after procedure) | Coca-Cola p.o. (b.i.d. 24h before and 24h after procedure) | Increase in SCr of > 25% | 24 and 96 | In-hospital and 4 days |
| **Diaz-Sandoval et al. (APART trial)[23]** | 2002 | 54 | - NAC 600 mg p.o. (b.i.d., 1 dose before and 3 doses after procedure) | Placebo p.o. (Isotonic saline p.o.; 1 dose before and 3 doses after procedure) | Increase in SCr of ≥ 0.5 mg/dL  or  > 25% (within 24h-48h after procedure) | 48 | In-hospital |
| **Briguori et al.[24]** | 2002 | 183 | - NAC 600 mg p.o. (b.i.d. day before and on the day of procedure) | Placebo p.o. (b.i.d. day before and on the day of procedure) | Increase in SCr of > 25%  or  the need for dialysis | 48 | In-hospital |
| **Shyu et al.[25]** | 2002 | 121 | - NAC 400 mg p.o. (b.i.d. day before and on the day of procedure) | Placebo p.o. (b.i.d. day before and on the day of procedure) | Increase in SCr of ≥ 0.5 mg/dL | 48 | 7d |
| **Boccalandro et al.[26]** | 2003 | 179 | - NAC 600 mg p.o. (b.i.d. day before and on the day of procedure) and Saline 0.45% i.v. (75 mL/h for 12h before and after procedure) | Saline 0.45% i.v.  (75 mL/h for 12h before and after procedure) | Increase in SCr of ≥ 0.5 mg/dL | 48 | In-hospital |
| **Kay et al.[27]** | 2003 | 200 | - NAC 600mg p.o. (b.i.d. for 2 days, three doses before and one after procedure) | Placebo p.o. (b.i.d. for 2 days, three doses before and one after procedure) | Increase in SCr of > 25% (within 24h, 48h, 168h after procedure) | 48 | 7 days |
| **Ueda et al.[28]** | 2011 | 60 | - Sodium bicarbonate 154 mEq/L 0.5 ml/kg bolus injection i.v. (at admission), followed by sodium bicarbonate 154 mEq/L i.v. (1 mL/kg/h during and for 6h after procedure) | Isotonic saline i.v. 0.5 ml/kg bolus injection i.v. (at admission), followed by sodium bicarbonate 154 mEq/L i.v. (1 mL/kg/h during and for 6h after procedure) | Increase in SCr of > 0.5 mg/dL  or  > 25% (within 48h, 72h after procedure and at the discharge) | 48 | In-hospital |
| **Ochoa et al.[29]** | 2004 | 80 | - NAC 1000 mg p.o. in 20 mL CocaCola (1h before and 4h after procedure) | Placebo in 20 mL CocaCola | Increase in SCr of ≥0.5 mg/dL  or  ≥25% (within 24h, 48h after catheterization) | 48 | 30 days |
| **Thiele et al. (LIPSIA-N-ACC trial)[30]** | 2010 | 251 | - NAC 1200 mg i.v. before procedure and again 1200 mg i.v. b.i.d. for 48h after procedure | 10 mL isotonic saline i.v. before procedure and again b.i.d. for 48h after procedure | Increase in SCr of ≥25% (within 24h, 48h, 72h after procedure) | 72 | 6 months |
| **Webb et al.[31]** | 2004 | 487 | - NAC 500 mg i.v. (in 50 mL of 5% dextrose; over 15 min starting 1h before procedure) and Isotonic saline i.v. (200 mL before and 1.5 mL/kg/h for 6h after procedure or until discharge) | 50 mL of 5% dextrose, and isotonic saline i.v. (200 mL before and 1.5 mL/kg/h for 6h after procedsure or until discharge) | Decline in CCr of >5 mL/min | 48 | 2-8 days |
| **Carbonell et al.[32]** | 2007 | 216 | - NAC 600 mg i.v. (in 50 mL of isotonic saline; b.i.d., total of 4 doses, starting at least 6h before procedure) | 50 ml of isotonic saline i.v. (b.i.d., total of 4 doses, starting at least 6h before procedure) | Increase in SCr of ≥ 0.5 mg/dL  or  > 25% (within 24h, 48h after procedure) | 48 | In-hospital |
| **Koc et al.[33]** | 2012 | 220 | - NAC 600 mg bolus i.v. (b.i.d. before and on the day of procedure) and isotonic saline i.v. (1mL/kg/h before, on and after the day of procedure) | Isotonic saline i.v. (1 mL/kg/h for 12h before and after procedure or the day before until the day after procedure) | Increase in SCr of ≥ 0.5 mg/dL  and/or  ≥ 25% | 48 | In-hospital |
| **Amini et al.[34]** | 2009 | 90 | - NAC 600 mg p.o. (b.i.d. starting 24h before and after procedure) | Placebo p.o. (b.i.d. starting 24h before and after procedure) | Increase in SCr of ≥ 0.5 mg/dL  and/or  ≥ 25% | 48 | In-hospital |
| **Azmus et al.[35]** | 2005 | 414 | - NAC 600 mg p.o. (b.i.d. day before and on the day of procedure, and 1 dose the day after procedure) | Placebo p.o. (b.i.d day before and on the day of procedure and 1 dose the day after procedure | Increase in SCr of > 0.5 mg/dL  and/or  ≥ 25% | 24 to 48 | In-hospital |
| **Carbonell et al.[36]** | 2010 | 90 | - NAC 600 mg i.v. (in 50 mL of isotonic saline; b.i.d. for 30 min, total of 4 doses) | Placebo i.v. (in 50 mL of isotonic saline; b.i.d for 30 min, total of 4 doses) | Increase in SCr of ≥ 0.5 mg/dL  and/or  > 25% (within 24h, 48h after procedure) | 48 | In-hospital |
| **Coyle et al. (AID trial)[37]** | 2006 | 137 | - NAC 600 mg p.o. (b.i.d. 2 doses before and after procedure)  with 1L of clear fluids p.o. over 10h before procedure and saline 0.45% i.v. 300 mL/h for 6h after the procedure | 1L of clear fluids p.o. over 10h before procedure and saline 0.45% i.v. 300 mL/h for 6h after the procedure | Increase in SCr of ≥ 0.5 mg/dL | 48 to 96 | In-hospital |
| **Drager et al.[38]** | 2004 | 30 | - NAC 600 mg p.o. (b.i.d. on 4 consecutive days beginning 2 days prior to procedure) | Placebo p.o. (b.i.d. on 4 consecutive days beginning 2 days prior to procedure) | NA | NA | In-hospital |
| **Mahmoodi et al.[39]** | 2014 | 350 | - Sodium bicarbonate 154 mEq/L (in 5% dextrose with water, 6h before and after procedure) and NAC 1200 mg (day before and on the day of procedure) | Saline (6h before and 6h after proceure) and NAC 1200 mg day before and on the day of procedure) | Increase in SCr of > 0.5 mg/dL  and/or  ≥ 25% | 48 | In-hospital |
| **Ferrario et al.[40]** | 2009 | 215 | - NAC 600 mg p.o. (b.i.d. day before and the day of procedure) | Placebo p.o. (b.i.d. day before and the day of procedure) | Increase in SCr of > 0.5 mg/dL  and/or  ≥ 25% (within 24h, 48h, 72h after procedure) | 72 | In-hospital |
| **Fung et al.[41]** | 2004 | 91 | - NAC 400 mg p.o. (t.i.d. day before and day of procedure) with saline i.v. (100 mL/h from 12h before to 12h after procedure) | Saline i.v. (100 mL/h from 12h before to 12h after procedure) | Increase in SCr of ≥ 0.5 mg/dL  or  reduction in eGRF of  ≥ 25% | 48 | In-hospital |
| **Gomes et al.[42]** | 2004 | 156 | - NAC 600 mg p.o. (b.i.d. 1 day before and after procedure) | Placebo p.o. (b.i.d. 1 day before and after procedure | Increase in SCr of ≥ 0.5 mg/dL | 48 | In-hospital |
| **Gulel et al.[43]** | 2005 | 50 | - NAC 600 mg p.o (b.i.d. day before and on the day of procedure)  and isotonic saline i.v. (1 mL/kg/h for 12h before and after procedure) | Isotonic saline i.v. (1 mL/kg/h for 12h before and after procedure) | Increase in SCr of > 0.5 mg/dL | 48 | In-hospital |
| **Kim et al. (ENABLE trial)[44]** | 2010 | 166 | - NAC 600 mg p.o. (b.i.d. day before and on the day of procedure) and isotonic saline i.v. (1 mL/kg/h for 12h before and 6h after procedure) | Isotonic saline i.v. (1 mL/kg/h for 12h before and 6h after procedure) | Increase in SCystC of > 0.5 mg/dL or > 25%  or  Increase in SCr of > 0.5 mg/dl or >25% | 48 | In-hospital |
| **Kimmel et al. [45]** | 2008 | 36 | - NAC 600 mg p.o (b.i.d. day before and the day of procedure) | Placebo p.o. (b.i.d. day before and day of procedure) | Increase in SCr of ≥ 0.5 mg/dL  and/or  ≥ 25% | 48 | In-hospital |
| **Kotlyar et al.[46]** | 2005 | 60 | - NAC 300 or 600 mg i.v. (in 5% dextrose; over 20 min 1–2h before and 2–4h after procedure) with isotonic saline i.v. (200 ml/h, 2h before and 5h after procedure) | Isotonic saline i.v. (200 ml/h, 2h before and 5h after procedure) | Increase in SCr of > 0.5 mg/dL  and/or  > 25% (within 48h,72h,96h and 30 days after procedure) | 48 | 30 days |
| **MacNeill et al.[47]** | 2003 | 43 | - NAC 600 mg p.o (2 doses, ﬁrst at the time of randomization, the second 4h later and 3 doses at 12-h intervals after procedure) | Placebo p.o. (2 doses, ﬁrst at the time of randomization, the second 4h later and 3 doses at 12-h intervals after procedure) | Increase in SCr of >25% (within 24h, 48h, 72h after procedure) | 72 | In-hospital |
| **Marenzi et al.[48]** | 2006 | 354 | - NAC 600-1200 mg i.v. (before procedure) and NAC 600-1200 mg p.o. (b.i.d. for 48h after procedure) | Placebo | Increase in SCr of ≥25% (within 24h, 48h, 72h after procedure and at hospital discharge) | 72 | In-hospital |
| **Namgung et al. [49]** | 2005 | 48 | - NAC 600 mg p.o. (b.i.d. before and after procedure) and saline 0.45% i.v. (1 mL/kg/h) | Saline 0.45% i.v. (1 mL/kg/h) | Increase in SCr of > 0.5mg/dL | 48 | In-hospital |
| **Seyon et al. [50]** | 2007 | 40 | - NAC 2400 mg p.o. (4 doses of 600 mg, one before and three after procedure) | Placebo p.o. (4 doses of 600 mg, one before and three after procedure) | Increase in SCr of > 0.5 mg/dL  and/or  > 25% | 48 | In-hospital |
| **Sinha et al. [51]** | 2004 | 70 | - NAC 600 mg p.o. (b.i.d. day before and the day of procedure) | Placebo p.o. (b.i.d day before and the day of procedure) | Increase in SCr of > 0.5 mg/dL (within, 24h, 48h, 168h after procedure) | 48 | In-hospital |
| **Erturk et al. [52]** | 2014 | 307 | - NAC 1200 mg p.o. (b.i.d. for 24h before and 48h after procedure) or NAC 7200 mg i.v (2400 mg 1h before procedure; 4800 mg within 4-6h after procedure)  with isotonic saline i.v. (1 mL/kg/h for 12h before and after procedure; 0.5 mL/kg/h for LVEF<35%) | Isotonic saline i.v. (1 mL/kg/h for 12h before and after procedure; 0.5 mL/kg/h for LVEF<35%) | Increase in SCr of > 0.5mg/dL  And/or  > 25%  or  cystatin C  > 0.5mg/dL  and/or  > 25%  Increase in SCr of > 0.3mg/dL  and  cystatin C  > 10% | 48 | 30 days, 1 year |
| **Merten et al. [53]** | 2004 | 137 | - Sodium bicarbonate 154 mEq/L i.v (in 5% dextrose in water; 3 mL/kg/h 1h before procedure; 1 mL/kg/h during procedure and for 6h after procedure) | Isotonic Saline i.v. (3 mL/kg/h 1h before procedure; 1 mL/kg/h during procedure and for 6h after procedure) | Increase in SCr of ≥ 25% (within 24h, 48h after procedure)  Increase in SCr of ≥ 0.5mg/dL | 24 or 48 | In-hospital |
| **Boucek et al. [54]** | 2013 | 126 | - Sodium bicarbonate 154 mL 8.4% in 846 mL 5% glucose (3mL/kg/h 1h before procedure, max. 330 mL; 1mL/kg/h for 6h after procedure, max. 660 mL) | Isotonic Saline i.v. (3mL/kg/h 1h before procedure, max. 330 mL; 1mL/kg/h for 6h after procedure, max. 660 mL) | Increase in SCr of ≥ 0.5 mg/dL  and/or  ≥ 25% | 48 | 1 month |
| **Masuda et al. [55]** | 2007 | 61 | - Sodium bicarbonate 154 mEq/L i.v.(3 mL/kg/h for 1h before procedure; 1 mL/kg/h for 6h during and after procedure) | Isotonic Saline i.v. (3 mL/kg/h for 1h before procedure; 1 mL/kg/h for 6h during and after procedure) | Increase in SCr of ≥ 0.5 mg/dL and/or  ≥ 25% | 48 | 1 year |
| **Adolph et al. (REINFORCE trial) [56]** | 2008 | 145 | - Sodium bicarbonate 154 mEq/L i.v. in 5% dextrose solution (2 mL/kg/h 2h before procedure, 1 mL/kg/h during and for 6h after procedure) | Isotonic saline i.v. (2 mL/kg/h for 2h before and 1 ml/kg/h during and for 6h after procedure) | Increase in SCr of > 0.5 mg/dL  or  > 25% (within 24h, 48h after procedure) | 24 or 48 | 10 to 14 days |
| **Pakfetrat et al.[57]** | 2009 | 192 | - Sodium bicarbonate 154 mL mEq/L i.v. in 5% dextrose (3 mL/kg/h 1h before procedure, 1 mL/kg/h for 6h after procedure) | Isotonic saline i.v. (1 mL/kg/h for 6h before and after procedure) | According to RIFLE criteria | 48 | In-hospital |
| **Hengel et al.[58]** | 2006 | 72 | - Sodium bicarbonate i.v. (3 mL/kg/h 1h before and 1 mL/kg/h 6h after procedure) | Isotonic saline i.v. (3 mL/kg/h 1h before and 1 mL/kg/h 6h after procedure) | Increase in SCr of > 0.5 mg/dL  or  > 25% | 48 to 72 | In-hospital |
| **Zhou and Chen[59]** | 2012 | 174 | - Ascobic acid 5g i.v. and p.o. (3g i.v before and 0.5g b.i.d. for 2 days after procedure) in addition to saline 1mg/kg 4h before and 12h after procedure | Isotonic saline i.v. (1mg/kg/h 4h before and 12h after procedure) | Increase in SCr of ≥0.5 mg/dL  and/or  ≥ 25% | 48 | In-hospital |
| **Tamura et al.[60]** | 2008 | 144 | - Sodium bicarbonate 20 mEq i.v. 5 min before procedure in addition Sodium chloride 0.9% 1mL/kg/h 12h before and 12h after procedure | Isotonic saline i.v. (1mL/kg/h 12h before and 12h after procedure) | Increase in SCr of ≥0.5 mg/dL  and/or  ≥ 25% | 72 | 1 week |
| **Vasheghani-Farahani et al.1[61]** | 2010 | 72 | - Sodium bicarbonate 8.4% 75 mL added to 1 L saline 0.45% 3 mL/kg/h i.v. 1h before and 1 mL/kg/h i.v. 6h after procedure | Saline 0.45% i.v. (3 mL/kg/h 1h before and 1 mL/kg/h 6h after procedure) | Increase in SCr of ≥0.5 mg/dL  and/or  ≥ 25% | 48 | In-hospital |
| **Vasheghani-Farahani et al.2[62]** | 2009 | 265 | - Sodium bicarbonate 8.4% 75 mL added to 1 L isotonic saline 3 mL/kg/h i.v. 1h before and 1 mL/kg/h i.v. 6h after procedure | Isotonic saline 3 mL/kg/h i.v. 1h before and 1 mL/kg/h i.v. 6h after procedure | Increase in SCr of ≥0.5 mg/dL  and/or  ≥ 25% | 48 | In-hospital |
| **Motohiro et al.[63]** | 2011 | 158 | - Sodium bicarbonate 154 mEq/L in 5% dextrose 1 mL/kg/h i.v.3h before and 6h after procedure and sodium chloride 0.9% 1mL/kg/h 9h before and 9h after sodium bicarbonate | Sodium chloride 0.9% 1mL/kg/h 12h before and 12h after procedure | Increase in SCr of ≥0.5 mg/dL  and/or  > 25% | 48 | 1 month |
| **Klima et al. [64]** | 2012 | 273 | -Sodium bicarbonate 166 mEq/L 3 mL/kg/h 1h before and 1 mL/kg/h during and 6h after procedure  -Sodium bicarbonate 166 mEq/L 3 mL/kg i.v. before and 500 mL mineral water within 6h p.o. after procedure | Sodium chloride 0.9% 1mL/kg/h 12h periprocedural | Increase in SCr of ≥0.5 mg/dL  or  ≥ 25% | 48 | In-hospital, 90 days |
| **Maiolli et al. [65]** | 2011 | 308 | - Sodium bicarbonate 154 mEq/L i.v (3 mL/kg in 1h, 1 mL/kg/h 12h after procedure; 0.5 mL/kg/h in patients with LVEF ≤40% and/or NYHA class III-IV) | Saline (1 mL/kg/h 12h after procedure; 0.5 mL/kg/h in patients with LVEF ≤40% and/or NYHA class III-IV) | Increase in SCr of > 0.5 mg/dL  and/or  ≥ 25% | 72 | In-hospital |
| **Manari et al.[66]** | 2014 | 592 | - Sodium bicarbonate 154 mEq/L i.v. (1 mL/kg/h for 12h or 3 mL/kg/min for 1h, then 1 mL/kg/min for 11h starting before procedure) | Normal saline i.v. (1mL/kg/h for 12h or 3 mL/kg/min for 1h, then 1 mL/kg/min for 11h starting before procedure) | Increase in SCr of  ≥ 25% | 72 | 30 days 1 year |
| **Spargias et al.[67]** | 2004 | 238 | - Ascorbic acid 7g p.o., 3g 2h before procedure and 2g in the night and the morning after procedure | Placebo p.o. | Increase in SCr of ≥0.5 mg/dL  and/or  ≥ 25% | 48 to 120 | In-hospital |
| **Boscheri et al.[68]** | 2007 | 143 | - Ascorbic acid 1g p.o. 20 min before procedure | Placebo p.o. | Increase in SCr of > 25% | 48 | In-hospital |
| **Komiyama et al.[69]** | 2011 | 70 | - Ascorbic acid 7g i.v., 3g before and 4g after procedure | Saline 0.9% i.v. (1500-2500 mL 12h after procedure) | Increase in SCr of ≥0.5 mg/dL  and/or  ≥ 25% | NA | In-hospital |
| **Li and Chen[70]** | 2012 | 149 | - Ascorbic acid 5g, 3g i.v. 2–4h before and 2g p.o after procedure,  1g p.o. on day 1 and 2 after procedure | Sodium chloride 0.9% | Increase in SCr of ≥0.5 mg/dL  and/or  ≥ 25% | 48 | In-hospital |
| **Hamdi et al.[71]** | 2013 | 202 | - Ascorbic acid 7g p.o., 3g 2h before and  2g on day 1 and 2 after procedure | Saline | Increase in SCr of > 25% | 48 to 72 | In-hospital |
| **Han et al.[72]** | 2013 | 2998 | - Rosuvastatin 50mg p.o., 10mg each 2 days before and 3 days after procedure) | Sodium Chloride 0.9% 1mL/kg/h 12h before and 24h after procedure | Increase in SCr of ≥0.5 mg/dL  and/or  ≥ 25% | 72 | 30 days |
| **Patti et al. (ARMYDA-CIN trial) [73]** | 2011 | 270 | - Atrovastatin 120 mg p.o. , 80mg 12h and 40mg 2h before procedure | Placebo p.o. | Increase in SCr of ≥0.5 mg/dL  and/or  ≥ 25% (within 24h, 48h after catheterization) | 48 | In-hospital |
| **Acikel et al.[74]** | 2010 | 160 | - Atorvastatin 40mg p.o. 3 days before and for 2 days after procedure | Saline 0.9% i.v. 1 mL/kg/h 4 h before and 24h after procedure procedure | NA | 48 | In-hospital |
| **Jo et al. (PROMISS trial)[75]** | 2008 | 247 | - Simvastatin 40mg p.o. b.i.d. for 2 days after procedure | Placebo p.o. | Increase in SCr of ≥0.5 mg/dL  and/or  ≥ 25% | 48 | 1 month  6 month |
| **Oliveira et al.[76]** | 2012 | 135 | - Rosuvastatin 40 mg p.o. 2-6h before procedure | Sodium Cholride 0.9% | Increase in SCr of ≥0.5 mg/dL  and/or  ≥ 25% | 24 | In-hospital |
| **Li et al.[77]** | 2012 | 161 | - Atorvastatin 80 mg p.o. 90 min before procedure | Placebo p.o. | NA | 48  72 | In-hospital |
| **Solomon et al.[78]** | 1994 | 78 | - Mannitol 25g i.v. (60 min before procedure) and Saline 0.45% i.v. (1 mL/kg/h 12 h before and after procedure)  - Furosemide 80 mg i.v. (30 min before procedure) and Saline 0.45% i.v. (1 mL/kg/h 12 h before and after procedure) | Saline 0.45% i.v.  (1 mL/kg/h for 12h before and after procedure) | Increase in SCr of ≥0.5 mg/dL | 48 | In hospital |
| **Majumdar et al.[79]** | 2009 | 92 | - Furosemide 100 mg and Mannitol 25g i.v. (in 500ml 0.45% Saline and 15mmol KCl; started before procedure; 4h in total) | Saline 0.45% and 15mmol KCl i.v. (started before procedure; 4h in total) | Increase in SCr of > 0.5 mg/dL  or  > 25% (within 24h, 48h after procedure, at hospital discharge and 6 weeks later) | 48 | 6 weeks |
| **Gu et al.[80]** | 2013 | 859 | - Furosemide 20 mg i.v. after procedure | Saline 0.9% i.v. (1mL/kg/h 12h before and 24h after procedure) | Increase in SCr  > 0.5 mg/dL  or  > 25% | 48 | In-hospital |
| **Yin et al. [81]** | 2013 | 217 | - Probucol 1000 mg p.o. before procedure and 500 mg b.i.d. for 3 days after procedure  with Saline 0.9% i.v. (1 mL/kg/h for 24h after procedure) | Isotonic saline i.v.  (1 mL/kg/h for 24h after procedure) | Increase in SCr of ≥ 0.5mg/dL  And/or  ≥ 25%  or  Cy C  > 10% | 72 | In-hospital |
| **Li et al. [82]** | 2009 | 205 | - Probucol 500 mg p.o. (b.i.d. 3 days before and after procedure) | Saline 0.9% i.v. (1 mL/kg/h for 12h after procedure) | Increase in SCr of ≥ 0.5mg/dL  or  ≥ 25% | 72 | In-hospital |
| **Abizaid et al. [83]** | 1999 | 40 | - Aminophylline 4 mg/kg i.v. 0.4 mg/kg/h | Saline 0.45% i.v.  (1 mL/kg/h for 12h before and 12h after procedure) | Increase in SCr of ≥ 25% (within 24h, 48h after procedure) | 24  48 | In-hospital |
| **Rohani et al. [84]** | 2010 | 60 | - Aminophylline 250 mg i.v. 30 min before procedure | Isotonic crystalloid i.v. (1.0-1.5 mL/kg/h for 3-12h before and 6-24h after procedure) | Increase in SCr of > 0.5 mg/dL (within 24h, 48h after procedure) | 48 | In-hospital |
| **Kapoor et al. [85]** | 2002 | 70 | - Theophylline 200 mg p.o. b.i.d. 24h before and for 48h after procedure | No treatment | Increase in SCr of ≥ 25%  or  Reduction of GFR ≥ 25% | 48 | In-hospital |
| **Huber et al. [86]** | 2003 | 100 | - Theophylline 200 mg i.v. in 100 mL Isotonic saline 30 min before procedure | Placebo (100 mL Saline) | Increase in SCr of ≥ 0.5mg/dL  (within 12h, 24h, 48h after procedure) | 48 | In-hospital |
| **Matejka et al. [87]** | 2010 | 58 | - Theophylline 205.7 mg i.v. in 500 mL Saline 0.9% 90 min before procedure | Placebo i.v. (500 mL Saline 0.9%) | Increase in SCr of > 0.5 mg/dL  or  > 25% | 48 | In-hospital |
| **Stone et al. [88]** | 2003 | 315 | - Fenoldopam i.v. (0.05 ug/kg/min titrated to 0.10 ug/kg/min in 20min if tolerated; 1h before and for 12h after procedure) | Placebo | Increase in SCr of ≥ 25% (within 24h, 48 to 60h, 72 to 96h after procedure) | 24 to 96 | 30 days |
| **Lee et al. [89]** | 2007 | 82 | - Isotonic saline 200 ml i.v. and hemodialysis (duration of dialysis 4h, dialysate flow 500 mL/min) | No hemodialysis | Increase in SCr of > 25% | 96 | In-hospital |
| **Vogt et al. [90]** | 2001 | 113 | - Hemodialysis after procedure (mean duration 3.1h, dialysate flow 500 mL/min) and saline i.v. (1/mL/kg/h for 12h before procedure) | No treatment | Increase in SCr of  > 1.5 mg/dL  or  > 50% | 24 to 144 | In-hospital |
| **Marenzi et al. [91]** | 2003 | 114 | - Pre/post -Hemofiltration (4-6h before procedure continued for 18-24h after procedure; dialysate flow 1000 mL/h), Hemofiltration treatment stopped during the coronary procedure | Isotonic saline at 1 ml/h/kg or 0.5 ml/h/kg if LVEF<40% for 6-8 h before and 24 h after procedure | Increase in SCr of ≥ 25% (within 24h, 48h, 72h after procedure and at hospital discharge) | 24  48  72  At hospital discharge | 1 year |
| **Marenzi et al. [92]** | 2006 | 92 | - Post-hemofiltration: i.v. hydration with isotonic saline(12h before contrast agent exposure and hemofiltration for 18-24h after procedure) or  - Pre/post-hemofiltration: Hemofiltration 6h before and for 18-24h after procedure | Isotonic saline 1 ml/h/kg or 0.5 ml/h/kg if reduced LVEF for 12 h before and after procedure | Increase in SCr of ≥ 25% (within 24h, 48h, 72h after procedure and at hospital discharge) | 24 to 72 | In-hospital |
| **Lehnert et al. [93]** | 1998 | 30 | - Haemodialysis as soon as possible (63±6 min) after last bolus of contrast | No haemodialysis | Increase in SCr of ≥ 0.5mg/dL | 48 | In-hospital |
| **Berger et al. [94]** | 2001 | 15 | - Haemodialysis for 2-3h after procedure as quickly as possible (106±6 min) | No haemodialysis | Increase in SCr of ≥ 0.5mg/dL | 48 | In-hospital |
| **Weisberg et al. [95]** | 1994 | 35 | - ANP i.v. (50 ug bolus, and 1 ug/min)  - Mannitol 15 g/dL i.v.  with 0.45% saline i.v. (100ml/h, beginning 12 h before and continuing throughout the procedure) | 0.45% saline i.v. (100ml/h, beginning 12 h before and continuing throughout the procedure) | Increase in SCr of ≥ 25% (within 24h, 48h, 72h after procedure and daily thereafter until it returned to the baseline level or stabilized) | 48 | In-hospital |
| **Morikawa et al. [96]** | 2009 | 261 | - ANP i.v. (0.042 ug/kg/min 4-6h before procedure and for 48h after procedure)  with Ringer i.v. (1.3 mL/kg/h for 4-6h before and for 48h after procedure) | Ringer i.v.  (1.3 mL/kg/h for 4-6h before and for 48h after procedure) | Increase in SCr of > 0.5 mg/dL or > 25% | 48 | 1 month |
| **Spargias et al. [97]** | 2009 | 208 | - Iloprost i.v. (1 ng/kg/ min 30-90 min before, ending 4h after procedure)  with normal saline i.v. (1.5 mL/kg/1h at least 4 h before and continued for 12 h after procedure) | Normal saline i.v.  (1.5 mL/kg/1h at least 4 h before and continued for 12 h after procedure) | Increase in SCr of ≥ 0.5 mg/dL or ≥ 25% | 48 to 120 | In- hospital |
| **Spargias et al. [98]** | 2006 | 45 | - Iloprost i.v. (1 or 2 ng/kg/min 30-90 min before and ending 4h after procedure)  with normal saline i.v. (1.5 mL/kg/1h at least 4 h before and continued for 12 h after procedure) | Normal saline i.v.  (1.5 mL/kg/1h at least 4 h before and continued for 12 h after procedure) | Increase in SCr of ≥ 0.5 mg/dL or ≥ 25% | 48 to 120 | In-hospital |
| **Li et al. [99]** | 2014 | 163 | - PGE1 i.v. (20 ng/kg/min for 6h, starting 1h before procedure) | Conventional hydration treatment | Increase in SCr of ≥ 0.5 mg/dL or ≥ 25% | 12, 24, 48 | In-hospital |
| **Briguori et al. (REMEDIAL trial) [100]** | 2007 | 351 | - Isotonic saline i.v. (1 mL/kg/h or 0.5 mL/kg for patients with LVEF < 40% for 12h before and after procedure) and NAC 1200 mg p.o. (b.i.d. day before and on the day of procedure) and Ascorbic acid 7 g i.v. (3 g 2h before and 2 g the night and the morning after procedure)  - Sodium bicarbonate 154 mEq/L i.v. (3 mL/kg/h for 1h before procedure, 1 mL/kg/h during and for 6h after procedure) and NAC 1200 mg p.o. (b.i.d. day before and on the day of procedure) | Isotonic saline i.v. (1 mL/kg/h or 0.5 mL/kg/h for patients with LVEF < 40% for 12h before and after procedure) and NAC 1200 mg p.o. (b.i.d. day before and on the day of procedure) | Increase in SCr of ≥ 25% | 48 | 1 week |
| **Recio-Mayoral et al. (RENO trial) [101]** | 2007 | 111 | - Preprocedural: Sodium bicarbonate 154 mEq/L i.v. (in 5% glucose and water 5 mL/kg/h) and Alkaline saline and NAC 2400 mg i.v. (over 1h after procedure). Postprocedural: Sodium bicarbonate 154 mEq/L i.v. (in 5% glucose and water 5 mL/kg/h) and Alkaline saline (1.5 mL/kg/h in the 12h after procedure) and NAC 600 mg p.o. (b.i.d. day after procedure) | Isotonic saline i.v. (1 ml/kg/h for 12h after procedure) and 2 oral doses of 500 mg NAC the next day | Increase in SCr of ≥ 0.5 mg/dL | 72 | 7 days |
| **Maioli et al. [102]** | 2008 | 502 | - Sodium bicarbonate 154 mEq/L i.v. (3 mL/kg for 1h before and 1 mL/kg/h for 6h after procedure; 0.5 mL/kg/h in patients with LVEF <40% or NYHA class III–IV) and NAC 600 mg p.o. b.i.d. | Isotonic saline i.v. (1 ml/kg/h for 12 h before and after procedure) and NAC 600 mg p.o. b.i.d. | Increase in SCr of ≥ 0.5 mg/dL (within 24h, 48h, 72h, 96h, 120h, and 240h after procedure) | 120 | In-hospital |
| **Heguilen et al. [103]** | 2007 | 18 | - Sodium bicarbonate 154 mEq/L i.v. (3 mL/kg/h 1h before and 1 mL/kg/h for 6h after procedure) | Isotonic saline 154 mEq/L i.v. (3 mL/kg/h for 1h before and 1 mL/kg/h for 6h after procedure) and NAC 600 mg p.o. (b.i.d. day before and on the day of procedure) | Increase in SCr of ≥ 25% | 48 to 72 | 2 weeks |
| **Lee et al. (PREVENT trial) [104]** | 2011 | 382 | - Sodium bicarbonate 154 mEq/L i.v. (1h before procedure, starting at 3 mL/kg/h and decreasing to 1 mL/kg/h during procedure and for 6h after completion of procedure; 0.5 mL/kg/h if LVEF <45%) and NAC 1200 mg p.o. (b.i.d. for 2 days) | Isotonic saline i.v. (1 ml/kg/h or 0.5 ml/kg/h LVEF < 45% for 12 h before and after procedure) and NAC 1200 mg p.o. (b.i.d. for 2 days) | Increase in SCr of > 0.5 mg/dL or > 25% | 48 | 1 month to 6 months |
| **Jo et al. (NASPI trial) [105]** | 2009 | 212 | - NAC 1200 mg p.o. (b.i.d. for 2 days - twice before procedure, starting the evening before procedure, and twice after procedure, beginning the evening of the day of procedure) | Ascorbic acid 9 g p.o. (3 g and 2 g before procedure with 12h time interval and 2 g b.i.d. after procedure) | Increase in SCr of ≥ 0.5 mg/dL or ≥ 25% | 48 | 1 month  and  6 months |
| **Toso et al. [106]** | 2010 | 304 | - Atorvastatin 80 mg p.o (2 days before and after procedure) | Placebo p.o. | Increase in SCr of ≥ 0.5 mg/dL | 24, 48, 72, 96, 120, and 240 | 1 month |
| **Ozhan et al. [107]** | 2010 | 130 | - Atorvastatin 80 mg p.o. (b.i.d. the first day; o.d. for 2 days after procedure) and NAC 600 mg p.o. (b.i.d. the first day) | NAC 600 mg p.o. (b.i.d. the first day) | Increase in SCr of > 0.5 mg/dL or > 25% | 48 | In-hospital |
| **Bilasy et al.[108]** | 2012 | 60 | - Theophylline 200 mg i.v. (in 100 mL isotonic saline; 30 min before procedure) | Isotonic saline i.v. (100 mL, 30 min. before procedure) | Increase in SCr of ≥ 0.5 mg/dL or≥ 25% | 72 | In-hospital |
| **Heng et al.[109]** | 2008 | 60 | - NAC 1200 mg p.o. (3 times before and once 1 after procedure) | Placebo p.o. | Increase in SCr of ≥ 0.5 mg/dL and/or ≥ 25% and/or decline GFR ≥ 5mL/min | 24 or 48 | In- hospital |
| **Quintavalle et al.[110]** | 2012 | 410 | - Atorvastatin 80 mg p.o. (within 24h before procedure) | No treatment | Increase in cystatin C  > 10% | 24 | In-hospital  1 year |
| **Briguori et al. (REMEDIAL II trial) [111]** | 2011 | 294 | Isotonic saline i.v. by RenalGuard hydratation system (preprocedural 250 mL over 30 min or ≤150 mL if LVEF ≤30% and/or unstable hemodynamic conditions; during procedure and for 4h after) and NAC 1500 mg i.v. (during RenalGuard therapy) | Sodium bicarbonate 154 mEq/L i.v (3 mL/kg/h 1h before procedure; 1 mL/kg/h during and for 6h after procedure) and NAC 1200 mg p.o. (b.i.d. day before and the day of procedure) and NAC 1200 mg i.v. (during procedure) | Increase in SCr of ≥ 0.3 mg/dL  or  need for dialysis | 48 | 1 month |
| **Han et al.[112]** | 2013 | 147 | - Atorvastatin 40 mg p.o (at bedtime) and Probucol 250 mg (t.i.d) and atorvastatin 40 mg and 500 mg probucol 2 h before procedure | - Atorvastatin 40 mg p.o (at bedtime) and atorvastatin 40 mg 2 h before procedure | NA | 48 | In-hospital |
| **Liu et al.[113]** | 2013 | 156 | - Alprostadil 20 µg/d i.v. (for 7d, starting 1d before procedure) and statins p.o. (atorvastatin, rosuvastatin, simvastatin, fluvastatin) | Statins p.o. (atorvastatin, rosuvastatin, simvastatin, fluvastatin) | Increase in SCr of ≥ 0.5 mg/dL and/or≥ 25%  (within 24h, 48h, 72h after procedure) | 48 | 6±1 months |
| **Luo et al. [114]** | 2014 | 216 | - Isotonic saline i.v. (1 mL/kg/h for 12h after procedure; 0.5 mL/kg/h if LVEF ≤ 30% or a Killip class 2 or 3) | No treatment | Increase in SCr of > 0.5 mg/dL or> 25% (within 24h, 48h, 72h after procedure) | 72 | In-hospital |
| **Onbasili et al. [115]** | 2007 | 82 | -Trimetazidine 20 mg p.o. (t.i.d. for 72h starting 48h before procedure) and Isotonic saline i.v. (1 mL/kg/h for 24h starting 12h before procedure) | Isotonic saline i.v. (1 mL/kg/h for 24h starting 12h before procedure) | Increase in SCr of > 0.5 mg/dL or > 25% | 24 or 48 | In-hospital |
| **Shehata et al. [116]** | 2014 | 100 | - Trimetazidine 35 mg p.o. (b.i.d. for 72h, starting 48h before procedure) and NAC 1200 mg p.o. (24h before and after procedure) and isotonic saline i.v. (1 mL/kg/h for 24h starting 12h before procedure) | Isotonic saline i.v. (1 mL/kg/h for 24h starting 12h before procedure) and NAC 1200 mg p.o. (24h before and after procedure) | Increase in SCr of ≥ 0.5 mg/dL or ≥ 25% | 72 | In-hospital |
| **Yeganehkhah et al. [117]** | 2014 | 150 | - Sodium bicarbonate i.v. (3 mL/kg/h before and 1 mL/kg/h within 6h after procedure)  - NAC 600 mg p.o. (b.i.d. 1 day before angiography and on the day of procedure) and isotonic saline (1 mL/kg/h Max. 100 mL/h for 12h before and after procedure) | Isotonic saline i.v. (1 mL/kg/h max. 100 mL/h for 12h  before and 12h after procedure) | Increase in SCr of ≥ 0.5 mg/dL or ≥ 25% | 48 | In-hospital |
| **Grygier et al.[118]** | 2011 | 152 | -NAC 1200 mg i.v. (in 250 mL saline; 5-10 min before procedure) and NAC 1200mg p.o. (b.i.d. 48h after PCI) and Saline i.v. (50-125 mL/h from randomization until 12h after procedure)  -NAC 1200 mg i.v. (in 250 mL saline; 5-10 min before procedure) and NAC 1200 mg p.o. (b.i.d. 48h after PCI) and Ascrobic acid 2000 mg p.o. (b.i.d. 48h after PCI) and Saline i.v. (50-125 mL/h from randomization until 12h after procedure) | Saline i.v. (50-125 mL/h from randomization till 12h after procedure) | increase in SCr ≥ 0.5 mg/dl or ≥25% | 48-120 | In-hospital |
| **Akgüllü et al.[119]** | 2015 | 123 | - Nebivolol 25 mg p.o. at least for a week | No treatment | Increase in SCr of ≥0.5 mg/dL and/or ≥25% | 48 and/or 120 | In-hospital |
| **Yang et al.[120]** | 2014 | 627 | - NAC 600 mg p.o. (b.i.d. 24h before and after procedure) and Sodium bicarbonate 1.5% i.v. (1.5 mL/kg/h 6h before and after procedure)  - NAC 600 mg p.o. (b.i.d. 24h before and after procedure) and Isotonic saline i.v. (1.5 mL/kg/h 6h before and after procedure)  - Sodium bicarbonate 1.5% i.v (1.5 mL/kg/h 6h before and after procedure) | Isotonic Saline i.v. (1.5 mL/kg/h 6h before and after procedure) | Increase in SCr of > 0.5 mg/dL or > 25% | 72 h | In-hospital |
| **Liu et al.[121]** | 2014 | 1000 | Human rBNP i.v. (0.005 µg/kg/min 24h before procedure) | Saline i.v. (1 mL/kg/h 24h before procedure) | Increase in SCr of ≥ 0.5 mg/dl or ≥ 25% (within 24h, 48h, 72h and 7 days after procedure) | 48 | 7 days |
| **Dvorsak et al. [122]** | 2013 | 83 | - Ascorbic acid p.o. (3 g before and 2 g after procedure) | Placebo p.o. | Increase in SCr of >25% or of SCystC >25% | 72-96h | In-hospital |
| **El Mahmoud et al. [123]** | 2003 | 120 | - NAC 600 mg p.o. b.i.d. the day before procedure with i.v. saline hydration | Saline hydration i.v. alone | Increase in SCr of >25% | 48h | In-hospital |
| **Brar et al. (Meena trial) [124]** | 2007 | 353 | - Sodium bicarbonate (3 amps −50 mEq each, + 1L 5% dextrose in water) | Isotonic saline | ≥ 25% reduction in GFR | 96h | 208 weeks |
| **Malhis et al. [125]** | 2010 | 280 | - Theophylline (p.o./i.v. 200 mg b.d. starting 24 h before radiography and continuing for 48 h thereafter; 200 mg theophylline a short infusion 30 minutes before radiography and continuing with 200 mg b.d of oral theophylline for 48 h thereafter) plus 1-2 L of intravenous sodium bicarbonate solution (150 meq/L) for 12 h after the procedure | 1-2 L of i.v. sodium bicarbonate solution (150 meq/L) for 12 h after the procedure | Increase in SCr of ≥ 0.5 mg/dL (in patients with a baseline serum creatinine <2 mg/dL) or an increase of >25% in baseline SCr (with a baseline serum creatinine ≥2 mg/dL) | 48h | In-hospital |
| **Dussol et al. [126]** | 2006 | 312 | - 5 mg/kg theophylline per os in one dose 1 h before the procedure and 0.9% saline intravenously at a rate of 15 ml/kg for 6 h before the procedure  - 3 mg/kg of furosemide intravenously just after the procedure and 0.9% saline intravenously at a rate of 15 ml/kg for 6 h before the procedure | 1 g/10 kg of body weight/day of sodium chloride per os for 2 days before the procedure or 0.9% saline i.v. at a rate of 15 ml/kg for 6 h before the procedure | Increase in the baseline SCr of ≥44 mmol/l (0.5 mg/dl) | 48h | In-hospital |
| **Shavit et al. [127]** | 2009 | 93 | - 154 mEq/L sodium bicarbonate in 5% dextrose in water mixed by adding 154 mL of 1,000 mEq/L sodium bicarbonate to 846 mL of 5% dextrose in water | sodium chloride (12-hour infusion of 154 mEq/L (0.9%) at a rate of 1 mL/kg per hour before cardiac catheterization) and NAC 600 mg × 2/d p.o. the day before and the day of the procedure | increase in SCr of ≥ 25% or ≥ 0.3 mg/dL from baseline | 48h | In-hospital |
| **Huber et al. [128]** | 2002 | 100 | - 200 mg theophylline administered intravenously as a short infusion 30 minutes before procedure | Saline administered intravenously as a short infusion 30 minutes before procedure | Increase in the baseline SCr of ≥44 mmol/l (0.5 mg/dl) | 48h | In-hospital |
| **Rashid et al. [129]** | 2004 | 94 | - NAC 1 g with normal saline i.v. (500 mL over 4 to 6h) 6 to 12h before and after angiography | Normal saline i.v. (500 mL over 4 to 6h) 6 to 12h before and after angiography | Increase in SCr of ≥0.5 mg/dL or ≥25% | 48h | 7 days |
| **Sherimani et al. [130]** | 2012 | 180 | - Furosemide | No treatment | Increase in the baseline SCr of ≥44 mmol/l (0.5 mg/dl) or ≥25% | 48h | In-hospital |
| **Brar et al. (POSEIDON) [131]** | 2014 | 396 | - Left ventricular end-diastolic pressure-guided volume expansion | standard fluid administration | Increase in the baseline SCr of >25% or 0·5 mg/dL | 96h | 6 months |
| **Briguori et al. [132]** | 2004 | 192 | - Fenoldopam 10µg/kg/min, started 1h before and continued for 12h after procedure | 0.45% Saline i.v. (1 ml/kg/h for 12h before and after procedure) and NAC 1200mg p.o. (b.i.d. the day before and on the day of procedure) | Increase in the baseline SCr ≥0.5 mg/dl or need for  dialysis | 48h | In-hospital |
| **Solomon et al. [133]** | 2015 | 391 | -Isotonic sodium bicarbonate (bolus of 5 mL/kg over 1 hour before the procedure followed by 1.5 mL/kg/hour during and for 4 hours after the angiogram) | isotonic sodium chloride (bolus of 5 mL/kg over 1 hour before the procedure followed by 1.5 mL/kg/hour during and for 4 hours after the angiogram) | Increase in the baseline SCr of ≥44 mmol/l (0.5 mg/dl) or ≥25% | 72h | 6 months |
| **Droppa et al. [134]** | 2011 | 251 | - NAC 1200 mg i.v. before and b.i.d. for 2d after procedure (total dose of 6000mg) | Placebo i.v. (10 ml NaCl 0,9%) | Increase in baseline SCr ≥ 25% | 72h | 6 months |
| **Abaci et al. [135]** | 2015 | 220 | - Rosuvastatin p.o. (40mg on admission, then 20mg/d) | No treatment | Increase in baseline SCr of ≥0.5 mg/dl or ≥25% | 48-72h | 1 year |
| **Balderramo et al. [136]** | 2004 | 61 | - NAC 1200 mg p.o. 3h before and after placebo | Placebo p.o. | Increase in baseline SCr of ≥0.5 mg/dl | 48h | In-hospital |
| **Koc et al. [137]** | 2013 | 195 | - Sodium bicarbonate i.v. | Saline i.v. | NA | NA | In-hospital |
| **Aslanger et al. [138]** | 2012 | 220 | - NAC 1200 mg i.v. during, then 1200 mg p.o. b.i.d. for 48h after procedure | Placebo i.v. (12 ml saline) | Increase in baseline SCr of ≥25% | 72h | In-hospital |
| **Gomes et al. [139]** | 2012 | 301 | - Sodium bicarbonate 154 mEq/l i.v. (3 ml/kg/h for 1h before, then 1 ml/kg/h during and for 6h after procedure) | Isotonic saline i.v. (3 ml/kg/h for 1h before, then 1 ml/kg/h during and for 6h after procedure) | Increase in baseline SCr of ≥0.5 mg/dl | 48h | In-hospital |
| **Heguilén et al. [140]** | 2013 | 133 | - NAC 600 mg b.i.d. the day before and the day of the procedure with NaHCO3 154 mmol/l i.v. (3ml/kg/h as bolus before, then 1ml/kg/h for 6-12h after procedure)  - NAC 600 mg b.i.d. the day before and the day of the procedure with isotonic saline i.v. (3ml/kg/h as bolus before, then 1ml/kg/h for 6-12h after procedure) | NaHCO3 154 mmol/l (3ml/kg/h as bolus before, then 1ml/kg/h for 6-12h after procedure) | Increase in baseline SCr of ≥25% | 48-72h | In-hospital |
| **INDA-Filho et al. [141]** | 2014 | 500 | - NAC in 500 ml of 5% dextrose i.v. at 150 mg/kg/h for 1 h before procedure, then at 50 mg/kg/h for 6 h after procedure  -150 mEq of sodium bicarbonate with 1 l of 5% dextrose i.v. at 3.5 mL/kg/h for 1 h before procedure, then at 1.18 ml/kg/h for 6 h after procedure  - NAC in 500 ml of 5% dextrose i.v. at 150 mg/kg/h for 1 h before procedure, then at 50 mg/kg/h for 6 h after procedure and 150 mEq of sodium bicarbonate with 1 l of 5% dextrose i.v. at 3.5 mL/kg/h for 1 h before procedure, then at 1.18 ml/kg/h for 6 h after procedure | Isotonic saline 0.9% at 1 ml/kg/h 1h before and 6h after procedure | -Increase in baseline sCr of ≥0.3mg/dl and/or both increase in baseline sCr and sCys of ≥10%  - Increase in baseline sCr and sCys of ≥0.3mg/dl or ≥25% | 48-72h | 72h |
| **LIU ET AL. [142]** | 2015 | 132 | -Trimetazidine 20 mg t.i.d orally 48 h before and 24 h after procedure and Isotonic saline at 1-1.5 ml/kg/h for 3 to 12 h before and up to 12 h after procedure | Isotonic saline at 1-1.5 ml/kg/h for 3 to 12 hours before  and up to 12 h after procedure | - Increase in baseline sCr of ≥0.5mg/dl or ≥25% | 48-72h | 1 year |
| **Rahman et al. [143]** | 2012 | 400 | -Trimetazidine 5 mg b.i.d for 48 h before and 48 h afters procedure | Normal saline at  1 ml/kg/h for 12 h before and 12 h after  Procedure | -- Increase in baseline sCr of ≥0.5mg/dl or ≥25% | NA | NA |
| **TANAKA ET AL. [144]** | 2011 | 76 | - NAC 705 mg p.o. the day before the intervention and after 12h, 24h and 36 h | Placebo (4 ml water) | Increase in baseline SCr of ≥25% | 72h | In-hospital |
| **NG ET AL. [145]** | 2006 | 95 | - Fenoldopam 1µg/kg/min i.v. initiated 1–2 h pre-catheterization and continued for 6 h post-catheterization | NAC 600 mg b.i.d., three doses the day before the procedure and one dose after | Increase in baseline SCr of ≥25% | 72h | In-hospital |
| **HUBER ET AL. [146]** | 2006 | 101 | - Theophylline 200 mg i.v. 30 min. before the procedure | NAC 600 mg i.v. b.i.d. the day before and the day of the procedure | Increase in baseline SCr of ≥25% | 48h | In-hospital |
| **Yavari et al. [147]** | 2014 | 199 | - Pentoxifylline 400mg p.o. t.i.d the day of the procedure and the day after, with isotonic saline at 1 ml/kg/h for 6h before, during and 6h after procedure | Isotonic saline at 1 ml/kg/h for 6h before, during and 6h after procedure | Increase in baseline SCr of ≥25% | 48h | In-hospital |
| AID - Acetylcysteine In Diabetes; APART - Acetylcysteine to Prevent Angiography-related Renal Tissue Injury; ARMYDA-CIN - Atorvastatin for Reduction of Myocardial Damage during Angioplasty-Contrast-Induced Nephropathy; CINSTEMI - Prevention of Contrast-Induced Nephropathy With N-Acetylcysteine or Sodium Bicarbonate in Patients With ST-Segment–Myocardial Infarction; ENABLE - Effect of N-acetylcysteine on cystatin C-based renal function after elective coronary angiography; LIPSIA-N-ACC - Leipzig Immediate PercutaneouS Coronary Intervention Acute Myocardial Infarction N-AC; MEENA - A Randomized Controlled Trial for the Prevention of Contrast-Induced Nephropathy with Sodium Bicarbonate in Persons Undergoing Coronary Angiography; MYTHOS - Induced Diuresis With Matched Hydration Compared to Standard Hydration for Contrast Induced Nephropathy Prevention; NASPI - N-acetylcysteine versus AScorbic acid for Preventing contrast-Induced nephropathy in patients with renal insufficiency undergoing coronary angiography; POSEIDON - Prevention of Contrast Renal Injury with Different Hydration Strategies; PRATO-ACS - Protective Effect of Rosuvastatin and Antiplatelet Therapy On Contrast-Induced Acute Kidney Injury and Myocardial Damage in Patients With Acute Coronary Syndrome; PREVENT - Preventive strategies of renal insufficiency in patients with diabetes undergoing intervention or arteriography; PROMISS - Prevention of radiocontrast medium-induced nephropathy using short-term high-dose simvastatin in patients with renal insufficiency undergoing coronary angiography; RAPPID - A rapid protocol for the prevention of contrast-induced renal dysfunction; REINFORCE - Renal Insufficiency Following Radiocontrast Exposure Trial; REMEDIAL - Renal Insufficiency After Contrast Media Administration Trial; REMEDIAL II - Renal Insufficiency After Contrast Media Administration Trial II; RENO - The reno-protective effect of hydration with sodium bicarbonate plus N-acetylcysteine in patients undergoing emergency percutaneous coronary intervention.  ANP - atrial natriuretic peptide; b.i.d. - twice a day; BNP - brain natriuretic peptide; CIAKI - contrast induced acute kidney injury; CCr - creatinine clearance; i.v. - intravenous; KCl- potassium chloride; LVEF - left ventricular ejection fraction; NA - not available; NAC - N-acetylcysteine; NYHA - New York Heart Association; p.o. - per os; SCr - serum creatinine; sCys - serum concentrations of cystatin C; t.i.d - three times a day. | | | | | | | |

**Appendix Table C. Patient characteristics.**

| **Study/authors [ref.]** | **clinical setting** | **Mean age,**  **years** | **Male (%)** | **Diabetes (%)** | **Baseline Serum Creatinine, mg/dL** | **Baseline creatinine clearance, ML/MIN** | **Baseline kidney disease (%)** |
| --- | --- | --- | --- | --- | --- | --- | --- |
| **PRATO-ACS [Leoncini M. et al][1]** | NSTEMI UA | 66.2 | 65.7 | 21.2 | 0.96 | 82.6 | 15.1 |
| **MYTHOS [Marenzi G] [2]** | NSTEMI UA Stable CAD | 73.0 | 78 | 44.0 | 1.80 | 38.0 | 100 |
| **Shaikh F et al. [3]** | Elective catheterization | 70.4 | 57.5 | 55.0 | 1.96 | 43.0 | NA |
| **CINSTEMI [Thayssen P et al][4]** | STEMI | 62.7 | 76.9 | 9.7 | 0.87 | 91 | NA |
| **Ozcan et al.[5]** | CAD (96.8%), HF | 69.0 | 74.6 | 45.1 | 1.39 | 49.7 | 100 |
| **Castini et al.[6]** | ACS  Chronic CAD | 71.1 | 87.8 | 26.9 | 1.55 | 48.4 | 100 |
| **Brueck et al.[7]** | NA | 74.6 | 63.7 | 47.3 | 1.50 | 41.5 | 100 |
| **Albabtain et al.[8]** | SA (53%)  UA (28%)  MI (14%)  VHD (3%)  CM (2%) | 61.1 | 73.0 | 84.0 | 1.29 | 66.8 | 100 |
| **Kinbara et al.[9]** | SA | 70.3 | 62.2 | 42.2 | 0.97 | 63.2 | NA |
| **Baskurt et al.[10]** | NA | 67.4 | 59.9 | 30.9 | 1.39 | 48.7 | 100 |
| **Allaqaband et al.[11]** | NA | 71.0 | 57.7 | 50.0 | 2.06 | 35.9 | 100 |
| **Loutrianakis et al.[12]** | NA | 67.0 | 76.0 | NA | 1.92 | NA | 100 |
| **Reinecke et al.[13]** | PVD (15.1%)  CAD (76.2) | 67.1 | 82.8 | 30.0 | 1.47 | 48.4 | 100 |
| **Gunebakmaz et al.[14]** | NA | 65.1 | 69.2 | 30.8 | 1.42 | 49.7 | 100 |
| **ACT Investigators [15]** | ACS (35.4%) | 68.1 | 61.4 | 60.4 | 1.20 | 67.7 | 15.7 |
| **Miner et al.[16]** | NA | 70.1 | 67.2 | 67.8 | 1.43 | 45.3 | 100 |
| **Goldenberg et al.[17]** | ACS(50%)  SA(25%)  DCM (12.5%)  Follow-up after heart transplantation(7.5%) | 70.0 | 82.5 | 43.7 | 1.95 | 39.5 | 100 |
| **Durham et al.[18]** | ACS (19.0%)  SA (19.0%)  MI (7.6%)  Preoperative evaluation (6.3%) | 70.6 | 65.8 | 48.1 | 2.25 | NA | 100 |
| **Oldemeyer et al.[19]** | CAD (42.5%) | 76.0 | 55.2 | 44.8 | 1.64 | 35.8 | 100 |
| **Baker et al. (RAPPID trial)[20]** | NA | 69.1 | 87.5 | 42.5 | 1.80 | 44.5 | 100 |
| **Kefer et al.[21]** | NA | 63.0 | 76.9 | 12.5 | 1.10 | NA | 59.3 |
| **Efrati et al.[22]** | CAD (46.9%)  PVD (42.9%)  History of stroke (4.2%) | 67.0 | 89.8 | 53.1 | 1.51 | 62.4 | 100 |
| **Diaz-Sandoval et al. (APART trial)[23]** | PVD (33%)  CAD (26%) | 73.0 | 79.6 | 38.9 | 1.60 | NA | 100 |
| **Briguori et al.[24]** | NA | 64.0 | 86.3 | 37.7 | 1.53 | 55.0 | 100 |
| **Shyu et al.[25]** | CAD | 70.0 | 67.8 | 63.6 | 2.80 | 23.4 | 100 |
| **Boccalandro et al.[26]** | NA | 65.4 | 60.3 | 61.5 | 1.86 | 51.6 | 100 |
| **Kay etal.[27]** | NA | 69.0 | 61.5 | 37.5 | 1.36 | 43.6 | 100 |
| **Ueda et al.[28]** | MI (67.8%)  UA (18.6%) | 76.0 | 78.0 | 15.3 | 1.41 | 40.6 | 100 |
| **Ochoa et al.[29]** | NA | 71.4 | 42.5 | 55.0 | 1.97 | 30.2 | 100 |
| **Thiele et al. (LIPSIA-N-ACC trial)[30]** | AMI | 68.0 | 68.1 | 29.1 | 0.90 | 85.5 | 0 |
| **Webb et al.[31]** | NA | 70.4 | 60.8 | 34.9 | 1.61 | 44.0 | 100 |
| **Carbonell et al.[32]** | ACS | 61.9 | 76.4 | 33.3 | 0.95 | 87.0 | 0 |
| **Koc et al.[33]** | NA | 63.7 | 77.3 | 34.1 | 1.30 | 59.7 | 100 |
| **Amini et al.[34]** | DM  CKD | 64.2 | 60 | 100 | 1.74 | 43.4 | 100 |
| **Azmus et al.[35]** | NA | 66.5 | 58.9 | 49.6 | 1.28 | 61.5 | 50.5 |
| **Carbonell et al.[36]** | ACS | 69.5 | 80.2 | 46.9 | 1.94 | 39.7 | 100 |
| **Coyle et al. (AID trial)[37]** | Angina/chest pain (62.8%)  ACS (8%) | 65.0 | 65.0 | 100 | 1.14 | 73.1 | 27 |
| **Drager et al.[38]** | NA | 65.0 | 83.3 | 37.5 | 1.78 | 45.5 | 100 |
| **Mahmoodi et al.[39]** | NA | 64.7 | 51.4 | NA | 1.17 | 64.8 | NA |
| **Ferrario et al.[40]** | NA | 74.9 | 65.0 | 25.0 | 1.60 | 42.5 | 100 |
| **Fung et al.[41]** | NA | 68.1 | 70.3 | 52.7 | 2.32 | 29.4 | 100 |
| **Gomes et al.[42]** | NA | 65.2 | 59.0 | 51.9 | 1.33 | 60.9 | 100 |
| **Gulel et al.[43]** | NA | 61.4 | 76.0 | 32.0 | 1.70 | 44.9 | 100 |
| **Kim et al. (ENABLE trial)[44]** | NA | 62 | 60.4 | 28 | 1.03 | NA | NA |
| **Kimmel et al.[45]** | CAD | 68.6 | 74.1 | 29.6 | 1.58 | 57.5 | 100 |
| **Kotlyar et al.[46]** | NA | 67.3 | 83.3 | 26.7 | 1.77 | 44.5 | 100 |
| **MacNeill et al.[47]** | NA | 72.5 | 86.1 | 46.5 | 1.88 | NA | 100 |
| **Marenzi et al.[48]** | STEMI | 62.0 | 80.7 | 15.34 | 1.03 | 77.6 | 26.5 |
| **Namgung et al.[49]** | NA | 66.6 | 64.6 | 50.0 | 2.06 | 31.7 | 100 |
| **Seyon et al.[50]** | ACS:  -UA high risk (27.5%)  -UA low risk (12.5%)  NSTEMI(45%)  STEMI(15%) | 75.6 | 65.0 | 40.0 | 1.49 | 35.7 | 100 |
| **Sinha et al. [51]** | NA | 70.9 | NA | 37 .0 | 2.04 | NA | 100 |
| **Erturk et al.[52]** | CAD  PAD  HF | 66.0 | 63.5 | 50.2 | 1.49 | 45.0 | 100 |
| **Merten et al.[53]** | NA | 67.9 | 74.8 | 47.9 | 1.80 | 43.0 | 100 |
| **Boucek et al.[54]** | NA | 65.0 | 75 .0 | 100 | 1.87 | 44.1 | 100 |
| **Masuda et al.[55]** | UA  MI  STEMI | 75.5 | 61.0 | 30.5 | 1.31 | 39.5 | 100 |
| **Adolph et al. (REINFORCE trial)[56]** | CAD (75%) | 71.4 | 77.9 | 33.8 | 1.56 | NA | 100 |
| **Pakfetrat et al.[57]** | NA | 57.9 | 61.2 | 29.4 | 1.10 | 72.2 | 13.3 |
| **Hengel et al.[58]** | SA  VHD  ACS | NA | NA | NA | 1.63 | NA | 100% |
| **Zhou and Chen[59]** | NA | 71.6 | 62.8 | 34.6 | 1.27 | 52.9 | 100 |
| **Tamura et al.[60]** | Elective coronary procedure | 72.8 | 87.5 | NA | 1.1 – 2.0 | NA | 100 |
| **Vasheghani-Farahani et al.1[61]** | ACS  HF | 62.1 | 79.2 | 34.7 | 1.74 | 43.5 | 100 |
| **Vasheghani-Farahani et al.2[62]** | NA | 63.3 | 83.0 | 21.5 | 1.64 | 45.9 | 100 |
| **Klima et al. [64]** | CAD (58%)  HF (44.6%) | 77.0 | 64.0 | 37.0 | 1.55 | 43.6 | 100 |
| **Motohiro et al.[63]** | Old HF (35.5%) | 72.5 | 69.7 | 60 | 1.54 | 44.3 | 100 |
| **Maiolli et al. [65]** | STEMI (100%) | 65.0 | 74.2 | 21.3 | 1.09 | 75.7 | 26.7 |
| **Manari et al.[66]** | STEMI (100%) | 64.8 | 74.8 | 16.6 | 1.01 | 81.3 | 15.88 |
| **Spargias et al.[67]** | HF (23.6%)  PVD (12.5%) | 65.5 | 92.2 | 25.1 | 1.41 | 64.5 | 100 |
| **Boscheri et al.[68]** | CAD (88.8%)  PVD(11.2%) | 71.0 | 72.0 | 59.4 | 1.74 | 40 | 100 |
| **Komiyama et al.[69]** | NA | NA | NA | NA | NA | NA | NA |
| **Li and Chen[70]** | NA | NA | NA | NA | NA | NA | NA |
| **Hamdi et al.[71]** | NA | 66.0 | 60.0 | NA | 1.12 | NA | NA |
| **Han et al.[72]** | NA | 61.4 | 65.2 | 100 | 1.07 | 74.3 | 100 |
| **Patti et al. (ARMYDA-CIN trial) [73]** | ACS(100%)  NSTEMI(38%)  UA(62%) | 65.5 | 77.6 | 28.2 | 1.04 | 78.4 | 30.7 |
| **Acikel et al.[74]** | Elective coronary angiography | 59.8 | 63.3 | 28.8 | 0.85 | 96.9 | 0 |
| **Jo et al. (PROMISS trial)[75]** | NA | 65.6 | 72.5 | 25.9 | 1.26 | 54.4 | 100 |
| **Oliveira et al.[76]** | CAD (100%) | 60.7 | 66.7 | 31.1 | 0.94 | 91.8 | 13.3 |
| **Li et al.[77]** | STEMI | NA | NA | NA | NA | NA | NA |
| **Solomon et al.[78]** | CAD  HF (28%) | 63.5 | 69.2 | 52.6 | 2.10 | NA | 100 |
| **Majumdar et al.[79]** | CAD(76%)  HF(17%) | 63.5 | 77.2 | 37.0 | 2.80 | 27.0 | 100 |
| **Gu et al.[80]** | CAD(36.3%)  MI (24.3%)  HF(1.2%) | 58.5 | 72.2 | 20.6 | 1.02 | 74.2 | 25.3 |
| **Yin et al.[81]** | ACS(100%) | 65.3 | 69.1 | 24.0 | 0.84 | 81.9 | 21.1 |
| **Li et al.[82]** | HF(16.9%) | 62.5 | 56.6 | 29.8 | 1.04 | 75.5 | 28.3 |
| **Abizaid et al.[83]** | CAD (100%) | 74.7 | 66.7 | 56.7 | 2.03 | NA | 100 |
| **Rohani et al.[84]** | NA | 61.8 | 80.5 | 17.9 | 1.76 | NA | 100 |
| **Kapoor et al.[85]** | NA | 53.2 | 91.4 | 100 | 1.18 | 86.1 | NA |
| **Huber et al.[86]** | NA | 68.8 | 83 | 31 | 1.69 | NA | 100 |
| **Matejka et al.[87]** | NA | 75.0 | 60.7 | 75.0 | 2.04 | 33.0 | 100 |
| **Stone et al.[88]** | PVD (35.2%)  UA (54.9%) | 69.6 | 66.0 | 48.6 | 1.81 | 29.1 | 100 |
| **Lee et al.[89]** | CAD(100%):  -SA(52%)  -ACS(48%) | 65.6 | 64.6 | 58.5 | 4.9 | 12.9 | 100 |
| **Vogt et al.[90]** | NA | 69.5 | 60.2 | 31.9 | 3.53 | 21.0 | 100 |
| **Marenzi et al.[91]** | Prior MI (29.8%)  Left ventricular ejection fraction <40% (24.5%) | 69.0 | 78.1 | 29.8 | 3.05 | 26.0 | 100 |
| **Marenzi et al.[92]** | Stable angina (29.3%)  Unstable angina (29.3%)  Recent MI(18,5%) | 71.7 | 70.7 | 30.4 | 3.63 | 19.3 | 100 |
| **Lehnert et al.[93]** | NA | 61.7 | 83.3 | 43.3 | 2.42 | 38.5 | 100 |
| **Berger et al.[94]** | NA | 65 | NA | 41.1 | 2.7 | 69.5 | 100 |
| **Weisberg et al.[95]** | NA | NA | NA | 48.0 | 2.50 | 33.5 | 100 |
| **Morikawa et al.[96]** | NA | 73.5 | 71.7 | 44.9 | 1.56 | 29.35 | 100 |
| **Spargias et al.[97]** | NA | 70.5 | 89 | 50.0 | 1.61 | 48.6 | 100 |
| **Spargias et al.[98]** | NA | 71.3 | 90.9 | 50.0 | 1.65 | 51.07 | 100 |
| **Li et al.[99]** | NA | 64.2 | 66.9 | 31.3 | 0.97 | 83.2 | NA |
| **Briguori et al. (REMEDIAL trial)[100]** | NA | 70.1 | 82.5 | 54.3 | 1.97 | 33.35 | 100 |
| **Recio-Mayoral et al. (RENO trial)[101]** | ACS | 64.5 | 69.5 | 29.5 | 1.0 | 74.5 | 8 |
| **Maioli et al.[102]** | NA | 73.7 | 59.0 | 24.1 | 1.20 | 42.50 | 100 |
| **Heguilen et al.[103]** | NA | 67.0 | 63.0 | NA | NA | NA | NA |
| **Lee et al. (PREVENT trial)[104]** | Silent myocardial ischemia  Stable angina  Unstable angina  Acute myocardial infarction | 68.0 | 70.9 | 100 | 1.50 | 46.0 | 100 |
| **Jo et al. (NASPI trial)[105]** | NA | 65.0 | 77.8 | 38.2 | 1.33 | 53.70 | 100 |
| **Toso et al.[106]** | NA | 75.5 | 64.5 | 21.0 | 1.19 | 46.00 | 100 |
| **Ozhan et al.[107]** | NA | 54.0 | 59.2 | 16.1 | 0.88 | 90.40 | 0 |
| **Bilasy et al.[108]** | CAD  HF | 57.0 | 60.0 | 50.0 | 1.44 | 60.20 | 63.35 |
| **Heng et al.[109]** | NA | 73.0 | 78.3 | 38.3 | 2.10 | 35.50 | 100 |
| **Quintavalle et al.[110]** | NA | 70.0 | 54.4 | 41.2 | 1.30 | 42.50 | 100 |
| **Briguori et al.(REMEDIAL II trial) II[111]** | NA | 75.5 | 65.4 | 70.2 | 1.80 | 32.00 | 100 |
| **Han et al.[112]** | CAD (100%)  ACS (86.8%) | NA | 59.1 | 24.09 | 0.94 | 80.01 | NA |
| **Liu et al.[113]** | CAD (17.9%)  AMI (11.5%) | 65.8 | 60.3 | 47.4 | 1.18 | 60.44 | 100 |
| **Luo et al.[114]** | STEMI | 67.0 | 65.7 | 25.0 | 0.87 | 70.55 | NA |
| **Onbasili et al.[115]** | ACS (56.1%)  SA (28.1%)  DCM (8.5%)  Preoperative assessment (7.3%) | 60.0 | 69.5 | 23.2 | 1.28 | 53.4 | 100 |
| **Shehata[116]** | SA (100%) | 59.0 | 68.0 | 100 | 2.0 | 48.5 | 100 |
| **Yeganehkhah et al.[117]** | NA | 59.2 | 52 | 40.7 | 1.14 | 63.9 | 46.7 |
| **Grygier et al.[118]** | ACS | 66.0 | 62.5 | 58.6 | 1.37 | 50.3 | NA |
| **Akgüllü et al.[119]** | NA | 57.6 | 49.5 | 27.7 | 0.83 | 94.0 | NA |
| **Yang et al.[120]** | Elective cardiovasculare procedures including CAG or interventional treatment | 59.0 | 53.9 | 22.2 | 0.79 | 93.2 | 8.21 |
| **Liu et al.[121]** | UA | 66.5 | 68.3 | 50.0 | 0.91 | 95.7 | 0 |
| **Dvorsak et al. [122]** | NA | NA | NA | NA | NA | NA | NA |
| **El Mahmoud et al. [123]** | NA | NA | NA | NA | 1.91 | NA | 100 |
| **Brar et al. (Meena trial) [124]** | NA | 71.0 | 65.0 | 44.0 | NA | NA | NA |
| **Malhis et al. [125]** | NA | 50.0 | 63.2 | 32.5 | 1.29 | NA | NA |
| **Dussol et al. [126]** | NA | 64.0 | 70.0 | 32.0 | 2.30 | 34.5 | 100 |
| **Shavit et al. [127]** | NA | 72.7 | 78.2 | 43.7 | 1.86 | 42.0 | 100 |
| **Huber et al. [128]** | NA | 67.5 | 36.0 | 34.0 | 1.95 | NA | NA |
| **Rashid et al. [129]** | NA | 70.4 | 63.83 | 26.6 | 1.33 | 143.5 | NA |
| **Sherimani et al. [130]** | NA | 65.5 | 50.5 | NA | NA | NA | NA |
| **Brar et al. (POSEIDON) [131]** | NA | 71.5 | 61.9 | 51.3 | 1.4 | NA | 100 |
| **Briguori et al. [132]** | Elective coronary and/or peripheral angiography | 68.5 | 85.0 | 51.0 | 1.73 | 42 | 100 |
| **Solomon et al. [133]** | Elective coronary and/or peripheral angiography | 72.0 | 57.5 | 54.0 | NA | 32.8 | 100 |
| **Droppa et al. [134]** | STEMI | 65.5 | 68.5 | 29.0 | 0.99 | 92 | NA |
| **Abaci et al. [135]** | Non-emergent elective coronary/peripheral angiography | 67.5 | 68.7 | 49.5 | 1.35 | 52.05 | 100 |
| **Balderramo et al. [136]** | NA | NA | NA | NA | 1.44 | NA | 100 |
| **Koc et al. [137]** | NA | NA | NA | 100 | NA | NA | NA |
| **Aslanger et al. [138]** | STEMI | 56.4 | 77.3 | 19 | 0.88 | 94.1 | NA |
| **Gomes et al. [139]** | NA | 64.3 | 72.0 | 29.3 | 1.49 | NA | 100 |
| **Heguilén et al. [140]** | NA | 67.2 | 72.4 | 36.7 | 1.55 | NA | 100 |
| **Inda-Filho et al. [141]** | Elective  coronary angiography or ventriculography | 59.4 | 60.6 | 22.0 | 1.03 | 76.6 | 24.5 |
| **LIU ET AL. [142]** | NA | NA | 56.8 | 60.6 | NA | NA | NA |
| **Rahman et al. [143]** | NA | 56.5 | 90.0 | 0 | NA | NA | 100 |
| **TANAKA ET AL. [144]** | STEMI | 61.7 | 82 | 29 | 0.84 | 84.5 | 27.6 |
| **NG ET AL. [145]** | Elective coronary angiography | 68 | 75.2 | 42.1 | 1.5 | NA | NA |
| **HUBER ET AL. [146]** | NA | 58.5 | 65.9 | 27.3 | 1.26 | NA | NA |
| **Yavari et al. [147]** | NA | 54.0 | NA | 25.2 | 1.05 | NA | NA |
| AID - Acetylcysteine In Diabetes; APART - Acetylcysteine to Prevent Angiography-related Renal Tissue Injury; ARMYDA-CIN - Atorvastatin for Reduction of Myocardial Damage during Angioplasty-Contrast-Induced Nephropathy; CINSTEMI - Prevention of Contrast-Induced Nephropathy With N-Acetylcysteine or Sodium Bicarbonate in Patients With ST-Segment–Myocardial Infarction; ENABLE - Effect of N-acetylcysteine on cystatin C-based renal function after elective coronary angiography; LIPSIA-N-ACC - Leipzig Immediate PercutaneouS Coronary Intervention Acute Myocardial Infarction N-AC; MEENA - A Randomized Controlled Trial for the Prevention of Contrast-Induced Nephropathy with Sodium Bicarbonate in Persons Undergoing Coronary Angiography; MYTHOS - Induced Diuresis With Matched Hydration Compared to Standard Hydration for Contrast Induced Nephropathy Prevention; NASPI - N-acetylcysteine versus AScorbic acid for Preventing contrast-Induced nephropathy in patients with renal insufficiency undergoing coronary angiography; POSEIDON - Prevention of Contrast Renal Injury with Different Hydration Strategies; PRATO-ACS - Protective Effect of Rosuvastatin and Antiplatelet Therapy On Contrast-Induced Acute Kidney Injury and Myocardial Damage in Patients With Acute Coronary Syndrome; PREVENT - Preventive strategies of renal insufficiency in patients with diabetes undergoing intervention or arteriography; PROMISS - Prevention of radiocontrast medium-induced nephropathy using short-term high-dose simvastatin in patients with renal insufficiency undergoing coronary angiography; RAPPID - A rapid protocol for the prevention of contrast-induced renal dysfunction; REINFORCE - Renal Insufficiency Following Radiocontrast Exposure Trial; REMEDIAL - Renal Insufficiency After Contrast Media Administration Trial; REMEDIAL II - Renal Insufficiency After Contrast Media Administration Trial II; RENO - The reno-protective effect of hydration with sodium bicarbonate plus N-acetylcysteine in patients undergoing emergency percutaneous coronary intervention.  ACS - acute coronary syndrome; AMI - acute myocardial infarction; CAG - coronary angiography; CAD - coronary artery disease; CKD - chronic kidney disease; CM - cardiomyopathy; DCM - dilated cardiomyopathy; DM - diabetes mellitus; HF - heart failure; MI - myocardial infarction; NA - not available; NSTEMI - non ST-segment elevation myocardial infarction; PAD - peripheral artery disease; PVD - peripheral vascular disease; SA - stable angina; STEMI - ST-segment elevation myocardial infarction; UA - unstable angina; VDH - valvular heart disease. | | | | | | | |

**Appendix Table D. Procedural characteristics.**

| **Study/AUTHORS [ref]** | **Cardiac procedure** | **PCI (%)** | **Contrast** | **Contrast type and osmolarity** | **Contrast volume (ml)** | **Contrast volume >140ml (%)** | **Hydration protocol** |
| --- | --- | --- | --- | --- | --- | --- | --- |
| **PRATO-ACS [Leoncini M. et al][1]** | Coronary angiography | 66.1 | Iodixanol | Non-ionic, dimeric, iso-osmolar | 144 | 43.3 | Isotonic saline i.v. (1 mL/kg/h; 0.5 mL/kg for patients with LVEF < 40%; for 12h before and after procedure) |
| **MYTHOS [Marenzi G] [2]** | Coronary angiography | 57 | Iomeprol | Non-ionic, low-osmolar | 169.8 | NA | Isotonic saline i.v. (1 mL/kg/h; 0.5 mL/kg/h for patients with LVEF < 40%) for 12h before and after procedure |
| **Shaikh F et al. [3]** | NA | NA | NA | NA | 117.3 | NA | Saline or Bicarbonate 154 mEq/L i.v. (3 mL/kg/h, for 1h before and 1 mL/kg/h for 6h after procedure) |
| **CINSTEMI [Thayssen P et al][4]** | Coronary angiography | 100 | Iodixanol | Non-ionic, dimeric, iso-osmolar | 140 | NA | Isotonic saline i.v. (≥ 60mL/h i.v. for a minimum of 6h) |
| **Ozcan et al.[5]** | Coronary angiography | 28.6 | Ioxaglate | Ionic, low-osmolar | 110.0 | NA | Bicarbonate or saline 154 mEq/L i.v.  (1 mL/kg/h, max. 100 mL/h for 6h before and after procedure) |
| **Castini et al.[6]** | Coronary angiography | 35.6 | Iodixanol | Non-ionic, iso-osmolar | 195.4 | NA | Bicarbonate or isotonic saline i.v. (1 mL/kg/h for 12h before and after procedure) |
| **Brueck et al.[7]** | Coronary angiography | NA | Iopromide | Non-ionic, Low-osmolar | 111.0 | NA | Isotonic saline i.v. (1.0 mL/kg/h for 12h before and after procedure) |
| **Albabtain et al.[8]** | Coronary angiography  PCI | NA | Ioxaglate | Ionic, low-osmolar | 87.6 | NA | Isotonic saline i.v. (50-125 mL/h from randomization until >6h after procedure; adjusted for LVEF and presence of clinical heart failure) |
| **Kinbara et al.[9]** | Coronary angiography  PCI | 44.4 | Iopamidol | Non-ionic, low-osmolar | 143.3 | NA | Isotonic saline i.v. (1 mL/kg/h for 30 min before and 10h after procedure) |
| **Baskurt et al.[10]** | Coronary angiography | NA | Ioversol | Non-ionic, low-osmolar | 119.9 | NA | Isotonic saline i.v.(1 mL/kg/ h for 12h before and after procedure) |
| **Allaqaband et al.[11]** | Coronary angiography  Peripheral angiography with percutaneous intervention | 27.0 | Ioversol  Iodixanol | Non-ionic, low-osmolar or iso-osmolar | 125 | NA | Saline 0.45% i.v. (1 mL/kg/h for 12h before and after procedure) |
| **Loutrianakis et al.[12]** | NA | NA | NA | NA | NA | NA | Saline 0.45% (1 mL/kg/h before and after procedure) |
| **Reinecke et al.[13]** | Left heart catheterization  Coronary angiography  PCI | NA | Iopromide | Non-ionic, iso-osmolar | 189.8 | NA | Glucose 5% 500mL i.v. and isotonic saline 500 mL i.v. (over 12h before and again after procedure) |
| **Gunebakmaz et al.[14]** | Coronary angiography  Ventriculography | 0 | Iopromide | Non-ionic, low-osmolar | 63.1 | NA | Isotonic saline i.v. (1 mL/kg/h, for 6h before and 12h after procedure) |
| **ACT Investigators [15]** | Coronary angiography | 28.8 | NA | High-osmolar 22.1%  low-osmolar 74%  iso-osmolar 2.9% | 100.0 | 22.6 | Isotonic saline i.v. (1 mL/kg/h, from 6-12h before to 6-12h after procedure; hydratation protocol could be adjusted) |
| **Miner et al.[16]** | Coronary angiography | 95.0 | Iohexol | Non-ionic, low-osmolar | 346.8 | NA | Saline 0.45% i.v. (75 mL/h for at least 24h beginning at the time of enrollment; changes in hydration were allowed at the discretion of the cardiologist) |
| **Goldenberg et al.[17]** | Coronary angiography | 45.0 | Iopamidol | Non-ionic, low-osmolar | 115.9 | NA | Saline 0.45% i.v. (1 mL/kg/h for 12h before and after procedure) |
| **Durham et al.[18]** | Coronary angography | NA | Iohexol | Non-ionic,low-osmolar | 81.2 | NA | Saline 0.45% i.v. (1 mL/kg/h up to 12h before and for up to 12h after procedure) |
| **Oldemeyer et al.[19]** | Coronary angiography  PCI | 33.3 | Iopamidol | Non-ionic,low-osmolar | 130.6 | NA | Saline 0.45% i.v. (1 mL/kg/h for 12h before and after procedure) |
| **Baker et al. (RAPPID trial)[20]** | Coronary angiography  PCI | 47.5 | Iodixanol | Non-ionic,iso-osmolar | 230.2 | NA | Isotonic saline i.v. (1 mL/kg/h for 12h befor and after procedure) |
| **Kefer et al.[21]** | Coronary angiography  PCI | NA | Iopromide or iohexol | Non-ionic,low-osmolar | 199.0 | NA | 5% Dextrose 500 mL i.v. (20 mL/h 12h before and for 24h after procedure) |
| **Efrati et al.[22]** | Coronary angiography  PCI | 40.8 | Iodine | Non-ionic, low-osmolar | 140.0 | NA | Saline 0.45% i.v. (1 mL/kg/h for 12h before and after procedure) |
| **Diaz-Sandoval et al. (APART trial)[23]** | Cardiac catheterization | NA | Ioxilan | Non-ionic, low-osmolar | 184.4 | NA | Saline 0.45% i.v. (1 mL/kg/h for 2-12h before and for 12h after procedure) |
| **Briguori et al.[24]** | Coronary angiography  PCI | 42.6 | Iopromide | Non-ionic, low-osmolar | 197.0 | 65.6 | Saline 0.45% i.v. (1 mL/kg/h for 12h before and after procedure) |
| **Shyu et al.[25]** | Coronary angiography PCI | 68.6 | Iopamidol | Non-ionic,low-osmolar | 117.0 | NA | Saline 0.45% i.v. (1 mL/kg/h for 12h before and after procedure) |
| **Boccalandro et al.[26]** | Cardiac catheterization | 36.3 | Iodixanol | Non-ionic,low-osmolar | 191.4 | NA | Saline 0.45% i.v. (75 mL/h for 12h before and after procedure) |
| **Kay etal.[27]** | Coronary angiography  PCI | 37 | Iopamidol | Non-ionic,low-osmolar | 139.0 | NA | Isotonic saline i.v. (1mL/kg/h for 12h before and for 6h after procedure) |
| **Ueda et al.[28]** | Coronary angiography PCI | 76.3 | Iopamidol  Iohexo | Non-ionic, low-osmolar | 110.1 | NA | Bicarbonate 154 mEq/L i.v. (1 mL/kg/h during and for 6h after procedure |
| **Ochoa et al.[29]** | Coronary angiography PCI | 52.5 | Diatrizoate  Ioxaglate  Iohexol  Iodixonal | Ionic, low-osmolar | 144.3 | NA | Isotonic saline i.v. (150 mL/h 4h before and for 6h after procedure; patients received a min. of 0.5 L/12h before procedure and min. of 1 L/24h after procedure) |
| **Thiele et al. (LIPSIA-N-ACC trial)[30]** | PCI | 100 | Iopromide | Non-ionic,low-osmolar | 180.0 | NA | Isotonic saline i.v. (1 mL/kg/h for 12h after procedure; 0.5 mL/kg/h in overt heart failure) |
| **Webb et al.[31]** | Cardiac catheterization | 34.7 | Ioversol | Non-ionic, low-osmolar | 120.0 | NA | Isotonic saline i.v. (200 mL before and 1.5 mL/kg/h for 6h after procedsure or until discharge) |
| **Carbonell et al.[32]** | Coronary angiography | 47.7 | Iopromide | Non-ionic, low-osmolar | 188.0 | NA | Saline 0.45% i.v. (6h before and for 12h after procedure;patients with congestive heart failure received a reduced hydration volume) |
| **Koc et al.[33]** | Coronary angiography  PCI | 24.5 | Iohexol | Non-ionic, low-osmolar | 138.0 | NA | Isotonic saline i.v. (1 mL/kg/h for 12h before and after procedure) |
| **Amini et al.[34]** | Coronary angiography | NA | Iohexol  Iodixanol  Diatrizoate meglumine/sodium | Non-ionic,low-osmolar (Iohexol)  Non-ionic, iso-osmolar (Iodixanol)  Ionic,high-osmolar (Diatrizoate meglumine/sodium) | 119.6 | NA | Fluids ≥ 1600 mL p.o. (over 12h before procedure) and Isotonic saline 1 L i.v. (started before procedure) |
| **Azmus et al.[35]** | Cardiac catheterization | 21.2 | Ioversol  Iohexol  Iopamidol  Diatrizoate  Iothalamate | Ionic, high-osmolar or  Non-ionic, low osmolar (4.3%) | 125.5 | >12.8 | Isotonic saline i.v. (1L before and after procedure) |
| **Carbonell et al.[36]** | Coronary angiography  PCI | 42.0 | Iopromide | Non-ionic, low-osmolar | 160.7 | NA | Saline 0.45% i.v. (1 mL/kg/h for 6h before and 12h after procedure) |
| **Coyle et al. (AID trial)[37]** | Coronary angiography | NA | NA | Non-ionic, low or iso-osmolar | 93.1 | NA | 1L of clear fluids p.o. over 10h before procedure and saline 0.45% i.v. 300 mL/h for 6h after the procedure |
| **Drager et al.[38]** | Coronary angiography | NA | Iopamidol | Non-ionic,low-osmolar | 101.1 | NA | Isotonic saline i.v. (2 mL/kg/h 4h before and 4h after procedure) |
| **Mahmoodi et al.[39]** | Coronary interventions | NA | Iohexol | Non-ionic, low‑osmolar | NA | NA | 1000 mL of either Sodium bicarbonate or Saline 6h before and 6h after procedure |
| **Ferrario et al.[40]** | Coronary angiography  PCI | 100 | Iodixanol | Non-ionic, iso-osmolar | 173.9 | 53.5 | Isotonic saline i.v. (1 mL/kg/h 12–24h before and 24h after procedure) |
| **Fung et al. [41]** | Coronary angiography  PCI | 5.2 | Iopromide | Non-ionic, low-osmolar | 128.5 | NA | Saline i.v. (100 mL/h from 12h before to 12h after procedure) |
| **Gomes et al. [42]** | Coronary angiography  PCI | 23.0 | Ioxaglate | Ionic, low-osmolar | 102.7 | NA | Isotonic saline i.v. (1 mL/kg/h from 12h before to 12h after procedure) |
| **Gulel et al. [43]** | Coronary angiography | 0 | Ioxaglat | Ionic, low-osmolar | NA | NA | Isotonic saline i.v. (1 mL/kg/h for 12h before and after procedure) |
| **Kim et al. (ENABLE trial) [44]** | Coronary Angiography  PCI | NA | Iodixanol (47%)  Iobitridol (27%)  Iopamidol (27%) | Non-ionic, iso-osmolar  non-ionic, low-osmolar  non-ionic, low-osmolar | 208.7 | NA | Isotonic saline (1 mL/kg/h for 12h before and 6h after procedure) |
| **Kimmel et al. [45]** | Coronary angiography | 0 | Iomeprol | Non-ionic, low-osmolar | 196.5 | NA | Saline 0.45% i.v. (1 mL/kg/h for 24h peri-procedurally) |
| **Kotlyar et al. [46]** | Coronary or peripheral angiography with or without stenting | 6.7 | Iopromide | Non-ionic, low-osmolar | 87.4 | NA | Isotonic saline i.v. (200 ml/h, 2h before and 5h after procedure) |
| **MacNeill et al. [47]** | Coronary angiography  PCI | 33.0 | Iopromide or Ioxilan | Non-ionic, low-osmolar | 110.0 | NA | Saline 0.45% i.v. (Pre-procedural 1 mL/kg/h for 12h for in-patients and 2mL/kg/h for 4h for day-case patients; Post-procedural: 75 mL/h for 12h) |
| **Marenzi et al. [48]** | Coronary angiography  PCI | 100 | Iohexol | Non-ionic,low-osmolar | 263.7 | NA | Isotonic saline i.v. (1 mL/kg/h for 12h after procedure; 0.5 mL/kg/h in cases of overt heart failure) |
| **Namgung et al. [49]** | Coronary angiography  PCI | 37.0 | NA | Non-ionic,low-osmolar | 160.5 | NA | Saline 0.45% i.v. (1 mL/kg/h) |
| **Seyon et al. [50]** | Coronary angiography  PCI | 92.5 | Iohexol (95%)  Iodixanol (5%) | Non-ionic, iso- or low-osmolar | 140.0 | NA. | Saline 0.45% i.v. (1 mL/kg/h 4-6h before and 12h after procedure) |
| **Sinha et al. [51]** | Coronary angiography  PCI | NA | Iodixanol  Iohexol | Non-ionic, iso- or low-osmolar | 129.4 | NA | Saline 0.45% i.v (1.5mL/kg/h for 8h before and after procedure) |
| **Erturk et al. [52]** | Coronary angiography  PCI | 13.3 | Iopromide | Non-ionic,low-osmolar | 125.0 | 33.2 | Isotonic saline i.v. (1 mL/kg/h for 12h before and after procedure; 0.5 mL/kg/h in patients with LVEF <35%) |
| **Merten et al. [53]** | Cardiac catheterization | NA | Iopamidol | Non-ioin, high-osmolar | 132.0 | NA | Bicarbonate or Saline 154 mEq/L i.v. (3 mL/kg/h 1h before procedure; 1 mL/kg/h during procedure and for 6h after procedure) |
| **Boucek et al. [54]** | Coronary or lower limb angiography with or without PCI | NA | Iodine | Non-ionic, low-osmolar | 109.6 | NA | Bicarbonate and Saline i.v. (3 mL/kg/h 1h before procedure, max. 330 mL; 1mL/kg/h for 6h after procedure,max. 660 mL) |
| **Masuda et al.[55]** | Coronary angiography  PCI | 61.0 | Iopamidol | Non-ionic,low-osmolar | 116.0 | NA | Bicarbonate and Saline 154 mEq/L i.v. (3 mL/kg/h for 1h before procedure; 1 mL/kg/h for 6h during and after procedure) |
| **Adolph et al. (REINFORCE trial) [56]** | Coronary angiography  PCI | NA | Iodixanol | Non-ionic, iso-osmolar | 139.5 | NA | Bicarbonate and Saline 154 mEq/L i.v. (2 mL/kg/h 2h before procedure; 1mL/kg/h during and for 6h after procedure= |
| **Pakfetrat et al. [57]** | Coronary angiography  PCI | 16.8 | Iodixanol | Non-ionic, iso-osmolar | 65.3 | NA | Saline 154 mEq/L i.v. (1 mL/kg/h for 6h before and after procedure) |
| **Hengel et al. [58]** | Coronary angiography  PCI | NA | NA | NA | 151.3 | NA | Bicarbonate and Saline i.v. (3 mL/kg/h 1h before and 1 mL/kg/h 6h after procedure) |
| **Zhou and Chen[59]** | Coronary angiography  PCI | 68.0 | Iodixanol Iopromide  Iohexol | Non-ionic, iso- or low-osmolar | 135.0 | NA | Isotonic saline i.v. (1 mg/kg/h for 4h before and at least 12h after procedure) |
| **Tamura et al.[60]** | Coronary angiography  PCI | NA | NA | NA | NA | NA | Sodium chloride 0.9% i.v. at 1ml/kg/h 12h before and 12h after procedure |
| **Vasheghani-Farahani et al.1[61]** | Coronary angiography | 0 | Iohexol | Non-ionic,low-osmolar | 117.5 | NA | Bicarbonate and Saline i.v. (3 mL/kg for 1h before procedure, 1 mL/kg/h for 6h after procedure) |
| **Vasheghani-Farahani et al.2[62]** | Coronary angiography | 0 | Iohexol  Iohexol with amidotrizoic acid  Iodixanol | Non-ionic, iso- or low-osmolar | 114.1 | NA | Bicarbonate and Saline i.v. (3 mL/kg for 1h before procedure, 1 mL/kg/h for 6h after procedure) |
| **Klima et al. [64]** | Coronary angiography  PCI | 21.0 | Iopromide  Iomeprol  Iopentol  Iohexol  Iobitridol  Isoosmolar Iodixanol | Non-ionic, iso- or low-osmolar | 100.0 | NA | Sodium chloride 0.9% 1mL/kg/h 12h periprocedural, sodium bicarbonate 166 mEq/L 3 mL/kg/h 1h before and 1 mL/kg/h during and 6h after procedure, sodium bicarbonate 166 mEq/L 3 mL/kg i.v. before and 500 mL mineral water within 6h p.o. after procedure |
| **Motohiro et al.[63]** | Coronary angiography  PCI | 48.4 | Iopamidol | Non-ionic, low-osmolar | 140.0 | NA | Isotonic saline i.v. (1 mL/kg/h before and after procedure) |
| **Maiolli et al. [65]** | PCI | 100 | Iodixanol | Non-ionic, iso-osmolar | 212.0 | NA | Isotonic saline i.v. (1 mL/kg/h for 12h after procedure; 0.5 mL/kg/h in patients with LVEF ≤40% and/or NYHA class III-IV) |
| **Manari et al.[66]** | PCI | 100 | Iodixanol | Non-ionic,Iso-osmolar | 198.2 | NA | Isotonic saline i.v. (1 mL/kg/min for 12h or 3 mL/kg/min for 1h, then 1 mL/kg/min for 11h) |
| **Spargias et al.[67]** | Coronary angiography  PCI | 41.1 | NA | Non-ionic, low or iso-smolar | 274.3 | NA | Isotonic saline i.v. (50-125 mL/h from randomization until at least 6h after procedure) |
| **Boscheri et al.[68]** | Coronary angiography  PCI | NA | Iodixanol | Non-ionic, iso-osmolar | 106.0 | 22.38 | Isotonic saline i.v. (500 mL 2h before procedure, 500 mL during and for 6h after procedure) |
| **Komiyama et al.[69]** | Emergency cardiac procedures | NA | NA | NA | NA | NA | Isotonic saline i.v. (total of 1500-2500mL) |
| **Li and Chen[70]** | Coronary angiography | NA | NA | NA | NA | NA | Routine hydratation with 0.9% Saline |
| **Hamdi et al.[71]** | Coronary angiography | NA | NA | NA | 71.6 | NA | Saline hydratation |
| **Han et al.[72]** | Cardiac catherization  Peripheral angiography  with or without stenting | 53.0 | Iodixanol | Non-ionic, iso-osmolar | 115.0 | NA | Isotonic saline i.v. (1 mL/kg/h 12h before and for 24h after procedure) |
| **Patti et al. (ARMYDA-CIN trial) [73]** | PCI | 100 | Iobitridol | Non-ionic, low-osmolar | 211.0 | NA | Patients with pre-existing renal failure: Isotonic saline i.v. (1 mL/kg/h for 12h before and 24h after procedure) |
| **Acikel et al.[74]** | Coronary angiography  PCI | 14.6 | Iohexol | Non-ionic,low-osmolar | 106.0 | NA | Isotonic saline i.v. (1.0 mL/kg/h 4h before and continuing until 24h after procedure) |
| **Jo et al. (PROMISS trial)[75]** | Coronary angiography  PCI | 28.7 | Iodixanol | Non-ionic, iso-osmolar | 182.1 | 58 | Saline 0.45% (1 mg/kg/h for 12h before and 12h after procedure) |
| **Oliveira et al.[76]** | PCI | 100 | NA | High- and low-osmolarity | 75.5 | NA | Isotonic saline i.v. in patients with baseline creatinine clearance < 60 ml/min (1 mL/kg/h for 6h before and 12h after procedure); 0.5 mL/kg/h in the case of left ventricular dysfunction |
| **Li et al.[77]** | PCI | 100 | Iodine | Non-ionic | NA | NA | Saline (0.9%) 1 ml/kg/h and 12 h after the procedure |
| **Solomon et al.[78]** | Coronary angiography | NA | NA | Ionic, low- and high-osmolar;  Non-ionic low-osmolar | 128.9 | NA | Saline 0.45% i.v. (1 mL/kg/h 12 h before and after procedure procedure) |
| **Majumdar et al.[79]** | Cardiac angiography  PCI | 28.3 | Ioxaglate | Ionic, low-osmolar | 140.5 | NA | Saline 0.45% 500ml and 15mmol KCl (2h before procedure) |
| **Gu et al.[80]** | Coronary angiography  PCI | 32.0 | NA | NA | 100 .0 | 8.96 | Saline i.v. (1mL/kg/h 4h before and for 24h after procedure) |
| **Yin et al.[81]** | PCI | 100 | Iopromide | Non-ionic, low-Osmolar | 163.1 | NA | Isotonic saline (1 mL/kg/h for 24h; 0.5 mL/kg/h in cases of overt heart failure) |
| **Li et al.[82]** | Coronary angiography  PCI | NA | Iohexol | Non-ionic, low-osmolar | 118.5 | NA | Isotonic saline (1 mL/kg/h for 12h; 0.5 mL/kg/h in cases of overt heart failure) |
| **Abizaid et al.[83]** | Coronary angioplasty | NA | Ioxaglate | Ionic, low-osmolar | 202.3 | NA | Saline 0.45% i.v. (1 mL/kg/h; 12h before and for 12h after procedure) |
| **Rohani et al.[84]** | Coronary angiography (54%) | NA | Iohexol | Non-ionic,low-osmolar | 205.0 | NA | Fluid supply of 2 L/day; Isotonic crystalloid i.v. (1.0-1.5 mL/kg/h for 3-12h before and for 6-24h after procedure) |
| **Kapoor et al.[85]** | Coronary angiography | NA | Diatrizoate  meglumine | high-osmolar | 79.0 | NA | Isotonic saline (1 mL/kg/h 12h before and for 12h after procedure) |
| **Huber et al.[86]** | Coronary angiography  PCI | NA | Iomeprol | Non-ionic, low-osmolar | 206.6 | NA | Fluid supply of 2 L/day; Additional hydration was performed according to clinical examination |
| **Matejka et al.[87]** | Cardiac catherization  PCI  Renal arteriography  Aortography | 39.3 | Iodixanol | Non-ionic,iso-osmolar | 94.6 | NA | Preprocedure Isotonic saline 500 mL i.v.. Postprocedure Isotonic saline i.v. 0.5 mL/kg/h after procedure 3 days after procedure |
| **Stone et al.[88]** | Coronary angiography  PCI | 34.6 | Iodixanol (10%) | Non-ionic,low-osmolar (90%) | 157.0 | NA | Saline 0.45% i.v. (1.5 mL/kg/h for 2-12h before drug/placebo; 1.0 mL/kg/h if heart failure was present) |
| **Lee et al.[89]** | Coronary, percutaneous transluminal renal and of the lower extremities  angiography and/or  angioplasty  CT and other radiographic interventions | 54.0 | Iohexol | Non-ionic, low-osmolar | 107.5 | NA | Isotonic saline i.v. (1 mL/kg/h for 6h before and 12h after procedure) |
| **Vogt et al.[90]** | Coronary angiography  PCI | NA | NA | Non-ionic,  low-osmolar | 175.6 | 49.56 | Isotonic saline i.v. (1mL/kg/h for 12h before and after procedure) |
| **Marenzi et al.[91]** | Coronary angiography  PCI | 86.8 | Iopentol | Non-ionic, low-osmolar | 252.4 | NA | Isotonic saline i.v. (1 mL/kg/h 6-8h before and 24h after procedure; 0.5 mL/kg/h if LVEF <40%) |
| **Marenzi et al.[92]** | Angiography | 28.3 | Iopentol | Non-ionic,low-osmolar | 230.7 | NA | isotonic saline i.v. 1 ml/h/kg (0.5 ml/h/kg if reduced left ventricular EF) 12 h before and after procedure |
| **Lehnert et al.[93]** | Angiography  Phlegbography  Coronary angiography  PCI  PTA | NA | Iopentol | Non-ionic, low-osmolar | 3.3 mL/kg | NA | Isotonic saline i.v. (83 mL/h beginning 12h before and for 12h after procedure) |
| **Berger et al.[94]** | Cardiac catherization | 6.7% | Iopromide | Non-ionic, low osmolar | 144 | 48.2% | 1000 ml 0.45% saline 12 h before procedure |
| **Weisberg et al.[95]** | Coronary angiography  PCI | NA | MD76  (66% diatrizoate meglumine, 10% diatrizoate sodium) | ionic, high-osmolar | 124.0 | NA | Saline 0.45% i.v. (100 mL/h) beginning 12 h before and throughout the cardiac catherization. |
| **Morikawa et al.[96]** | Coronary angiography  PCI | NA | Iomeprol | Non-ionic, low-osmolar | 139.5 | NA | Ringer i.v. (1.3 mL/kg/h 4-6h before and for 48h procedure) |
| **Spargias et al.[97]** | Coronary angiography  PCI | 47.1 | NA | Non-ionic,low- or iso-osmolar | 253.0 | NA | Isotonic saline i.v. (1.5 mL/kg/h at least 4h before and for at least 12h after procedure) |
| **Spargias et al.[98]** | PCI | 47.7 | NA | Non-ionic,low- or iso-osmolar | 227.0 | NA | Isotonic saline i.v. (1.5 mL/kg/h at least 4h before and for at least 12h after procedure) |
| **Li et al.[99]** | Coronary angiography  PCI | 100 | Iohexol | Non-ionic, low-osmolar | 170.0 | NA | Conventional hydration treatment |
| **Briguori et al. (REMEDIAL trial)[100]** | Coronary angiography  PCI | 51.8 | Iodixanol | Non-ionic, iso-osmolar | 172.4 | 56.1 | No standard hydratation |
| **Recio-Mayoral et al. (RENO trial)[101]** | Coronary angiography  PCI | 43.5 | Iomeprol | Non-ionic, low-osmolar | 284.5 | NA | No standard hydratation |
| **Maioli et al.[102]** | NA | 59.0 | Iodixanol | Non-ionic, iso-osmolar | 165.6 | 67.3 | No standard hydratation |
| **Heguilen et al.[103]** | Coronary angiography  PCI | NA | Ioversol | Non-ionic, low-osmolar | 284.5 | NA | No standard hydratation |
| **Lee et al. (PREVENT trial)[104]** | Coronary angiography  PCI | 45.8 | Iodixanol | Non-ionic, iso-osmolar | 116.5 | 27.25 | No standard hydratation |
| **Jo et al. (NASPI trial)[105]** | Coronary angiography  PCI | 31.6 | Iodixanol | Non-ionic, iso-osmolar | 210.0 | 69.6 | Saline 0.45% i.v. (1 mL/kg/h for 12h before and 12h after procedure) |
| **Toso et al.[106]** | Coronary angiography  PCI | 53.0 | Iodixanol | Non-ionic, iso-osmolar | 157.7 | 49.67 | Isotonic saline i.v. (1 mL/kg/h for 12h before and after procedure; 0.5 mL/kg/h if LVEF <40%) and NAC 1200mg p.o. (b.i.d. day before to the day after procedure) |
| **Ozhan et al.[107]** | Coronary angiography  PCI | 12.3 | Iopamidol | Non-ionic, low-osmolar | 94.8 | NA | Isotonic saline 1000 mL i.v. (during 6h after procedure) |
| **Bilasy et al.[108]** | Coronary angiography, ventriculography and angioplasty | 28.3 | Iopamidol | Non-ionic, low-osmolar | 116.8 | NA | Isotonic saline i.v. (1 mL/kg/h for 24h beginning 12h before procedure; 0.5 mL/kg/h if LVEF <40% or NYHA class III-IV) and NAC 600mg p.o. (b.i.d. day before and on the day of procedure) |
| **Heng et al.[109]** | coronary angiography, percutaneous coronary angioplasty, CTscan, angiography, Intra venous pyelography | NA | Iomeprol  Iomeron  Iodixanol | Non-ionic, low-osmolar (22%) or iso-osmolar (78%) | 202.0 | NA | Bicarbonate 1.4% i.v. (1 mL/kg/h for 12h before and for 12h procedure; 1 mL/kg/h for 6h before and for 6h after procedure in patients with heart failure) |
| **Quintavalle et al.[110]** | Coronary, peripheral angiography/  angioplasty | 64.4 | Iodixanol | Non-ionic, iso-osmolar | 180.5 | NA | Bicarbonate 154 mEq/L (in dextrose and water; 3 mL/kg/h for 1h before procedure; 1 mL/kg/h during and for 6h after procedure) and NAC 1200mg p.o. (b.i.d. day before and on the day of procedure) |
| **Briguori et al.(REMEDIAL II trial) II[111]** | Coronary angiography  PCI | 53.8 | Iodixanol | Non-ionic, iso-osmolar | 140.0 | NA | No standard hydratation |
| **Han et al.[112]** | Coronary angiography  PCI | 60.5 | Iopamidol  Iodinated | Non-ionic, low-osmolar | NA | NA | Initiating preprocedure hydration |
| **Liu et al.[113]** | Coronary angiography  PCI | 59.6 | Iodixanol | Non-ionic, iso-osmolar | 124.1 | NA | Isotonic saline i.v. (1-1.5 mL/ kg/h for 3-12h before and 6-24 h after procedure) |
| **Luo et al.[114]** | Coronary angiography  PCI | 100 | Iopamiron | Non-ionic, low-osmolar | 234.9 | 71.3 | No standard hydratation |
| **Onbasili et al.[115]** | Coronary angiography  PCI | 75.6 | Iopromide | Non-ionic,low-osmolar | 232.7 | NA | Isotonic saline i.v. (1 mL/kg/h for 24h starting 12h before procedure) |
| **Shehata[116]** | Coronary angiography | 100 | Iopromide | Non-ionic, low-osmolar | 275.0 | NA | Isotonic saline i.v. (1 mL/kg/h  starting 12h before and for 24 after procedure) and NAC 1200 mg 24 before and after procedure |
| **Yeganehkhah et al.[117]** | Coronary angiography  PCI | 0 | Iohexol | Non-ionic, Low-osmolar | 44.2 | NA | Isotonic saline i.v. (1 mL/kg/h max. 100 mL/h for 12h before and 12h after procedure) |
| **Grygier et al.[118]** | Coronary angiography | 100 | NA | NA | 171.8 | NA | Isotnic saline i.v. (50-125 mL/h from randomization till 12h after procedure) |
| **Akgüllü et al.[119]** | Coronary angiography  PCI | 0 | NA | NA | 110.8 | NA | Isotonic saline i.v. (1 mL/kg/h 12h before and after procedure) |
| **Yang et al.[120]** | Coronary angiography  PCI | NA | Iopromide | Non-ionic, hypotonic | 126.5 | NA | No standard hydratation |
| **Liu et al.[121]** | NA | 66.9 | Iopromide | Non-ionic | 123.5 | NA | Isotonic saline i.v. (1 mL/kg/h 24h before procedure) |
| **Dvorsak et al. [122]** | Coronary angiography  PCI | NA | NA | NA | NA | NA | Normal saline i.v. (50-100 mL/h for 2h before and 6h after procedure) |
| **El Mahmoud et al. [123]** | Coronary angiography | NA | NA | NA | NA | NA | Saline hydration i.v. |
| **Brar et al. (Meena trial) [124]** | Coronary angiography  PCI | 35.0 | NA | NA | 137 | 46.5 | Saline 0.9% hydration i.v. with or without N-acetylcysteine |
| **Malhis et al. [125]** | Coronary angiography  PCI  CT  Angiography  Intravenous pyelography | 21.1 | Iohexol, Iopamidol, Iodixanol | Low-osmolar nonionic or or isoosmolar nonionic | 140 | NA | 1-2 L of i.v. bicarbonate solution (150 meq/L) for 12 h after the procedure, hydration was performed according to clinical examination findings, radiographic evidence of pulmonary edema in patient with heart failure |
| **Dussol et al. [126]** | Coronarography  Angiography  Tomodensitometry | NA | Ioxaglate, Iobitridol, Iopromide | Low osmolality Non-ionic | 121.8 | NA | No standard hydration |
| **Shavit et al. [127]** | Coronary angiography  PCI | 27.6 | Iopamidol | Nonionic | 91.6 | NA | No standard hydration |
| **Huber et al. [128]** | Coronary angiography  Iliofemoral arteriography  Cerebrovascular arteriography  Celiacomesentericography  Transjugular portosystemic shunt placement  Computed tomography | 0 | Iomeprol  Imeron | Low osmolarity | NA | 100 | No standard hydration |
| **Rashid et al. [129]** | Coronary angiography | 0 | Iohexol | Non-ionic, Low-osmolar | 143.2 | NA | Normal saline (500 mL over 4 to 6h) 6 to 12h prior to again after angiography |
| **Sherimani et al. [130]** | PCI | 100 | NA | Low or iso-osmolar | 126.0 | NA | No standard hydration |
| **Brar et al. (POSEIDON) [131]** | cardiac catheterisation | 28.3 | Ioxilan | Non-ionic, Low-osmolar | 108.0 | NA | treatment: 5 mL/kg/h for left ventricular end-diastolic pressure <13 mm Hg, 3 mL/kg/h for pressure of 13–18 mm Hg, and 1·5 mL/kg/h for pressure >18 mm Hg. The control group 1·5 mL/kg/h. The fluid rate was set at the start of the procedure (before contrast exposure), continued for the duration of the procedure, and for 4 h post-procedure in both groups. |
| **Briguori et al. [132]** | Coronary angiography  PCI  Peripheral angiography  Peripheral angioplasty | 42.2 | Iodixanol | Non-ionic, iso-osmolar | 164.0 | 53 | Saline (0.45%) was given intravenously at a rate of 1 ml/kg of body weight/h (0.5 ml/kg for patients with left LVEF]40%) for 12 h before and 12 h after administration of the contrast agent |
| **Solomon et al. [133]** | Coronary angiography  peripheral angiography | NA | NA | NA | 107.0 | NA | No standard hydration |
| **Droppa et al. [134]** | Coronary angiography | 98.9 | NA | Iso-osmolar | 190.5 | NA | Isotonic saline i.v. at 1ml/kg/h for 12h after procedure (0.5ml/kg/h in heart failure) |
| **Abaci et al. [135]** | Non-emergent coronary or peripheral angiography | 0 | Optiray | Non-ionic, low-osmolar | 128.5 | 35.3 | Isotonic saline i.v. at 1 ml/kg/h for 12h before and 24h after procedure |
| **Balderramo et al. [136]** | Angiography | NA | NA | NA | NA | NA | Isotonic saline i.v. |
| **Koc et al. [137]** | Coronary angiography and/or intervention | NA | NA | NA | NA | NA | Saline or bicarbonate infusion |
| **Aslanger et al. [138]** | Coronary angiography  PCI | 100 | Hexabrix | Ionic, low-osmolar | 200.5 | NA | Isotonic saline i.v. at 1ml/kg/h for 12h (0.5ml/kg/h in heart failure) |
| **Gomes et al. [139]** | Elective coronary angiography  PCI | 16.95 | Hexabrix | Ionic, low-osmolar | 124.5 | 132 | Bicarbonate or isotonic saline i.v. at 3 ml/kg/h for 1h before, then 1 ml/kg/h during and for 6h after procedure |
| **Heguilén et al. [140]** | Coronary interventions | 73 | Ioversol | Non-ionic, low-osmolar | 192.5 | NA | Isotonic saline or NaHCO3 solution i.v. at 3 ml/kg/h for 2h before, then 1 ml/kg/h during and for 6-12h after procedure |
| **INDA-Filho et al. [141]** | Elective  coronary angiography or ventriculography | NA | Ioxitalamate | Ionic, high-osmolar | 90.2 | 7.2 | Isotonic saline 0.9% at 1 ml/kgh/h 1h before and 6h after procedure |
| **LIU ET Al. [142]** | Coronary angiography  PCI | 54.5 | Iodixanol | Non-ionic, iso-osmolar | NA | NA | Isotonic saline at 1-1.5 ml/kg/h for 3 to 12 hours before  and up to 12 h after procedure |
| **Rahman et al. [143]** | Coronary angiography  PCI | NA | NA | NA | NA | NA | Normal saline at  1 ml/kg/h for 12 h before and 12 h after  Procedure |
| **TANAKA ET AL. [144]** | Coronary angiography  PCI | 100 | Iopamidol | Non-ionic, low-osmolar | 210.5 | NA | Ringer lactate i.v. 1-2 ml/kg/h for more than 12 h after procedure |
| **NG ET AL. [145]** | Elective coronary angiography | NA | NA | Non-ionic, low or *iso*-osmolar contrast | 168.3 | 23 (contrast>150ml) | 0.45% saline at a rate of 1–1.5 mL/kg/h |
| **HUBER ET AL. [146]** | coronary angiography, computed tomography and peripheral angiografies | NA | Iomeprol | Non-ionic, low-osmolar | 156.8 | NA | Various, according to clinical data, laboratory results, radiographic evidence and hemodynamic findings |
| **Yavari et al. [147]** | Elective PCI | 100 | Iodixanol | Non-ionic, iso-osmolar | 188.9 | NA | Isotonic saline at 1 ml/kg/h for 6h before, during and 6h after procedure |
| AID - Acetylcysteine In Diabetes; APART - Acetylcysteine to Prevent Angiography-related Renal Tissue Injury; ARMYDA-CIN - Atorvastatin for Reduction of Myocardial Damage during Angioplasty-Contrast-Induced Nephropathy; CINSTEMI - Prevention of Contrast-Induced Nephropathy With N-Acetylcysteine or Sodium Bicarbonate in Patients With ST-Segment–Myocardial Infarction; ENABLE - Effect of N-acetylcysteine on cystatin C-based renal function after elective coronary angiography; LIPSIA-N-ACC - Leipzig Immediate PercutaneouS Coronary Intervention Acute Myocardial Infarction N-AC; MEENA - A Randomized Controlled Trial for the Prevention of Contrast-Induced Nephropathy with Sodium Bicarbonate in Persons Undergoing Coronary Angiography; MYTHOS - Induced Diuresis With Matched Hydration Compared to Standard Hydration for Contrast Induced Nephropathy Prevention; NASPI - N-acetylcysteine versus AScorbic acid for Preventing contrast-Induced nephropathy in patients with renal insufficiency undergoing coronary angiography; POSEIDON - Prevention of Contrast Renal Injury with Different Hydration Strategies; PRATO-ACS - Protective Effect of Rosuvastatin and Antiplatelet Therapy On Contrast-Induced Acute Kidney Injury and Myocardial Damage in Patients With Acute Coronary Syndrome; PREVENT - Preventive strategies of renal insufficiency in patients with diabetes undergoing intervention or arteriography; PROMISS - Prevention of radiocontrast medium-induced nephropathy using short-term high-dose simvastatin in patients with renal insufficiency undergoing coronary angiography; RAPPID - A rapid protocol for the prevention of contrast-induced renal dysfunction; REINFORCE - Renal Insufficiency Following Radiocontrast Exposure Trial; REMEDIAL - Renal Insufficiency After Contrast Media Administration Trial; REMEDIAL II - Renal Insufficiency After Contrast Media Administration Trial II; RENO - The reno-protective effect of hydration with sodium bicarbonate plus N-acetylcysteine in patients undergoing emergency percutaneous coronary intervention.  CT = computed tomography; LVEF = left ventricular ejection fraction; NA = not available; PCI = percutaneous coronary intervention. | | | | | | | |

**Appendix Table E**. Data synthesis for pairwise comparisons of primary and secondary outcomes.

| **Treatment comparison** | **Number of studies** | **Intervention** | | **Control** | |
| --- | --- | --- | --- | --- | --- |
|  |  | **Number of events** | **Number of patients** | **Number of events** | **Number of patients** |
| **Contrast-induced acute kidney injury** | | | | | |
| **saline+NAC vs saline** | 58 | 763 | 5576 | 886 | 5402 |
| **sodium bicarbonate vs saline** | 25 | 336 | 2703 | 381 | 2612 |
| **sodium bicarbonate+NAC vs saline** | 4 | 121 | 531 | 120 | 547 |
| **ascorbic acid vs saline** | 10 | 68 | 750 | 149 | 846 |
| **statin vs saline** | 7 | 57 | 2092 | 106 | 2099 |
| **furosemide vs saline** | 5 | 73 | 632 | 87 | 784 |
| **probucol vs saline** | 2 | 12 | 207 | 38 | 215 |
| **methylxanthines vs saline** | 11 | 35 | 608 | 66 | 706 |
| **fenoldopam vs saline** | 3 | 53 | 216 | 53 | 221 |
| **device-guided matched hydration vs saline** | 2 | 26 | 197 | 53 | 193 |
| **renal replacement therapy vs saline** | 7 | 67 | 377 | 92 | 347 |
| **nebivolol vs saline** | 2 | 13 | 100 | 16 | 103 |
| **natriuretic peptides vs saline** | 3 | 37 | 640 | 93 | 646 |
| **mannitol vs saline** | 2 | 10 | 35 | 9 | 43 |
| **prostaglandins vs saline** | 3 | 11 | 215 | 35 | 201 |
| **trimetazidine vs saline** | 3 | 14 | 302 | 49 | 312 |
| **LVEDP-guided hydration vs saline** | 1 | 12 | 196 | 28 | 200 |
| **sodium bicarbonate vs saline+NAC** | 9 | 159 | 832 | 130 | 815 |
| **sodium bicarbonate+NAC vs saline+NAC** | 11 | 182 | 1375 | 199 | 1380 |
| **ascorbic acid vs saline+NAC** | 5 | 42 | 433 | 77 | 544 |
| **statin vs saline+NAC** | 3 | 34 | 484 | 61 | 493 |
| **methylxanthines vs saline+NAC** | 4 | 1 | 168 | 19 | 168 |
| **fenoldopam vs saline+NAC** | 4 | 28 | 201 | 23 | 214 |
| **renal replacement therapy vs saline+NAC** | 1 | 18 | 138 | 6 | 146 |
| **nebivolol vs saline+NAC** | 1 | 8 | 40 | 9 | 40 |
| **trimetazidine vs saline+NAC** | 1 | 6 | 50 | 14 | 50 |
| **sodium bicarbonate+NAC vs sodium bicarbonate** | 6 | 126 | 603 | 140 | 623 |
| **ascorbic acid vs sodium bicarbonate+NAC** | 1 | 10 | 116 | 2 | 117 |
| **statin vs sodium bicarbonate+NAC** | 1 | 9 | 202 | 37 | 208 |
| **device-guided matched hydration vs sodium bicarbonate+NAC** | 1 | 16 | 147 | 30 | 147 |
| **probucol vs statin** | 1 | 4 | 54 | 4 | 93 |
| **prostaglandins vs statin** | 1 | 5 | 76 | 6 | 80 |
| **methylxanthines vs furosemide** | 1 | 2 | 80 | 5 | 79 |
| **mannitol vs furosemide** | 1 | 7 | 25 | 10 | 25 |
| **mannitol vs natriuretic peptides** | 1 | 3 | 10 | 5 | 10 |
| **Mortality** | | | | | |
| **saline+NAC vs saline** | 11 | 94 | 2645 | 89 | 2387 |
| **sodium bicarbonate vs saline** | 7 | 60 | 914 | 57 | 831 |
| **statin vs saline** | 1 | 3 | 1498 | 5 | 1500 |
| **furosemide vs saline** | 1 | 0 | 46 | 3 | 46 |
| **methylxanthines vs saline** | 1 | 0 | 50 | 1 | 50 |
| **fenoldopam vs saline** | 1 | 3 | 157 | 6 | 158 |
| **device-guided matched hydration vs saline** | 2 | 4 | 197 | 13 | 193 |
| **renal replacement therapy vs saline** | 4 | 13 | 313 | 30 | 284 |
| **prostaglandins vs saline** | 1 | 0 | 103 | 1 | 105 |
| **LVEDP-guided hydration vs saline** | 1 | 1 | 196 | 8 | 200 |
| **sodium bicarbonate+NAC vs saline+NAC** | 3 | 11 | 499 | 9 | 496 |
| **ascorbic acid vs saline+NAC** | 1 | 2 | 106 | 2 | 106 |
| **statin vs saline+NAC** | 2 | 3 | 424 | 3 | 423 |
| **methylxanthines vs saline+NAC** | 1 | 0 | 51 | 3 | 50 |
| **fenoldopam vs saline+NAC** | 1 | 1 | 95 | 0 | 97 |
| **renal replacement therapy vs saline+NAC** | 1 | 3 | 138 | 1 | 146 |
| **device-guided matched hydration vs sodium bicarbonate+NAC** | 1 | 6 | 146 | 6 | 146 |
| **Myocardial infarction** | | | | | |
| **saline+NAC vs saline** | 3 | 9 | 323 | 9 | 308 |
| **sodium bicarbonate vs saline** | 1 | 2 | 154 | 6 | 154 |
| **fenoldopam vs saline** | 1 | 5 | 157 | 3 | 158 |
| **device-guided matched hydration vs saline** | 1 | 0 | 90 | 1 | 86 |
| **renal replacement therapy vs saline** | 3 | 8 | 175 | 10 | 144 |
| **LVEDP-guided hydration vs saline** | 1 | 4 | 200 | 13 | 196 |
| **ascorbic acid vs saline+NAC** | 1 | 3 | 106 | 1 | 106 |
| **statin vs saline+NAC** | 1 | 2 | 271 | 5 | 272 |
| **Need for dialysis** | | | | | |
| **saline+NAC vs saline** | 12 | 16 | 2528 | 20 | 2270 |
| **sodium bicarbonate vs saline** | 10 | 22 | 1300 | 20 | 1211 |
| **statin vs saline** | 5 | 0 | 2168 | 7 | 2169 |
| **furosemide vs saline** | 2 | 6 | 133 | 7 | 129 |
| **methylxanthines vs saline** | 2 | 2 | 150 | 0 | 174 |
| **fenoldopam vs saline** | 1 | 4 | 137 | 3 | 146 |
| **device-guided matched hydration vs saline** | 1 | 0 | 108 | 3 | 108 |
| **renal replacement therapy vs saline** | 4 | 14 | 216 | 40 | 183 |
| **natriuretic peptides vs saline** | 1 | 0 | 130 | 1 | 131 |
| **LVEDP-guided hydration vs saline** | 1 | 1 | 196 | 4 | 200 |
| **sodium bicarbonate vs saline+NAC** | 1 | 1 | 88 | 0 | 88 |
| **sodium bicarbonate+NAC vs saline+NAC** | 1 | 1 | 116 | 1 | 117 |
| **ascorbic acid vs saline+NAC** | 2 | 7 | 222 | 2 | 223 |
| **methylxanthines vs saline+NAC** | 1 | 0 | 51 | 1 | 50 |
| **fenoldopam vs saline+NAC** | 1 | 1 | 95 | 0 | 97 |
| **renal replacement therapy vs saline+NAC** | 1 | 2 | 138 | 1 | 146 |
| **ascorbic acid vs sodium bicarbonate+NAC** | 1 | 4 | 116 | 1 | 117 |
| **device-guided matched hydration vs sodium bicarbonate+NAC** | 1 | 1 | 146 | 7 | 146 |
| **Heart failure** | | | | | |
| **saline+NAC vs saline** | 6 | 21 | 591 | 17 | 465 |
| **sodium bicarbonate vs saline** | 3 | 22 | 215 | 26 | 214 |
| **statin vs saline** | 1 | 39 | 1498 | 64 | 1500 |
| **furosemide vs saline** | 1 | 0 | 422 | 1 | 437 |
| **renal replacement therapy vs saline** | 2 | 2 | 120 | 3 | 86 |
| **natriuretic peptides vs saline** | 1 | 1 | 130 | 1 | 131 |
| **sodium bicarbonate+NAC vs saline+NAC** | 1 | 1 | 56 | 2 | 55 |
| **statin vs saline+NAC** | 1 | 1 | 152 | 0 | 152 |
| **sodium bicarbonate+NAC vs sodium bicarbonate** | 1 | 1 | 28 | 0 | 32 |

**Appendix Table F. Difference in treatment estimates from direct and indirect comparisons using node-splitting method and corresponding test for inconsistency.**

| **Treatment** | **Control** | **Estimates model** | **CIAKI overall**  **OR (95% CrI)** | **p-value** |
| --- | --- | --- | --- | --- |
| **Saline + NAC** | Saline | Direct | 0.70 (0.55, 0.88) | 0.51 |
|  |  | Indirect | 0.85 (0.49, 1.46) |  |
| **Sodium bicarbonate** | Saline | Direct | 0.72 (0.50, 1.03) | 0.20 |
|  |  | Indirect | 1.58 (0.50, 5.21) |  |
| **Sodium bicarbonate + NAC** | Saline | Direct | 1.05 (0.47, 2.36) | 0.06 |
|  |  | Indirect | 0.39 (0.21, 0.70) |  |
| **Ascorbic acid** | Saline | Direct | 0.49 (0.27, 0.88) | 0.11 |
|  |  | Indirect | 1.57 (0.42, 5.99) |  |
| **Statin** | Saline | Direct | 0.48 (0.23, 0.97) | 0.21 |
|  |  | Indirect | 0.25 (0.12, 0.53) |  |
| **Probucol** | Saline | Direct | 0.33 (0.12, 0.88) | 0.58 |
|  |  | Indirect | 0.64 (0.08, 5.42) |  |
| **Methylxanthines** | Saline | Direct | 0.49 (0.26, 0.92) | NA |
|  |  | Indirect | 0.03 (0.01, 0.26) |  |
| **Fenoldopam** | Saline | Direct | 0.88 (0.30, 2.51) | 0.37 |
|  |  | Indirect | 1.88 (0.51, 7.03) |  |
| **Device-guided matched hydration** | Saline | Direct | 0.33 (0.10, 1.05) | 0.82 |
|  |  | Indirect | 0.26 (0.05, 1.30) |  |
| **Prostaglandins** | Saline | Direct | 0.21 (0.06, 0.66) | 0.73 |
|  |  | Indirect | 0.31 (0.04, 2.25) |  |
| **Trimetazidine** | Saline | Direct | 0.23 (0.08, 0.69) | 0.99 |
|  |  | Indirect | 0.24 (0.04, 1.38) |  |
| **LVEDP hydration** | Saline | Direct | 0.54 (0.09-1.85) | NA |
|  |  | Indirect | not estimable |  |
| **Sodium bicarbonate** | Saline + NAC | Direct | 1.14 (0.63, 2.05) | 0.66 |
|  |  | Indirect | 0.95 (0.58, 1.57) |  |
| **Sodium bicarbonate + NAC** | Saline + NAC | Direct | 0.71 (0.42, 1.20) | 0.26 |
|  |  | Indirect | 1.48 (0.46, 4.57) |  |
| **Ascorbic acid** | Saline + NAC | Direct | 0.77 (0.33, 1.77) | 0.90 |
|  |  | Indirect | 0.72 (0.34, 1.52) |  |
| **Statin** | Saline + NAC | Direct | 0.51 (0.19, 1.36) | 0.93 |
|  |  | Indirect | 0.49 (0.25, 0.93) |  |
| **Methylxanthines** | Saline + NAC | Direct | 0.03 (0.01, 0.20) | NA |
|  |  | Indirect | not estimable |  |
| **Fenoldopam** | Saline + NAC | Direct | 1.19 (0.45, 3.13) | 0.78 |
|  |  | Indirect | 1.54 (0.33, 7.17) |  |
| **Renal replacement therapy** | Saline + NAC | Direct | 3.67 (0.72, 19.90) | NA |
|  |  | Indirect | not estimable |  |
| **Nebivolol** | Saline + NAC | Direct | 0.86 (0.14, 5.05) | 0.68 |
|  |  | Indirect | 1.48 (0.21, 10.40) |  |
| **Trimetazidine** | Saline + NAC | Direct | 0.34 (0.06, 1.95) | 0.99 |
|  |  | Indirect | 0.33 (0.11, 0.98) |  |
| **Sodium bicarbonate + NAC** | Sodium bicarbonate | Direct | 0.77 (0.38, 1.57) | 0.76 |
|  |  | Indirect | 0.66 (0.31, 1.39) |  |
| **Ascorbic acid** | Sodium bicarbonate + NAC | Direct | 6.42 (0.86, 69.40) | 0.06 |
|  |  | Indirect | 0.82 (0.40, 1.65) |  |
| **Statin** | Sodium bicarbonate + NAC | Direct | 0.21 (0.04, 1.01) | 0.13 |
|  |  | Indirect | 0.76 (0.38, 1.55) |  |
| **Device-guided matched hydration** | Sodium bicarbonate + NAC | Direct | 0.47 (0.10, 2.23) | 0.82 |
|  |  | Indirect | 0.59 (0.17, 2.05) |  |
| **Probucol** | Statin | Direct | 1.75 (0.22, 13.90) | 0.58 |
|  |  | Indirect | 0.90 (0.30, 2.77) |  |
| **Prostaglandines** | Statin | Direct | 0.85 (0.13, 5.64) | 0.74 |
|  |  | Indirect | 0.58 (0.15, 2.08) |  |
| **Methylxanthines** | Furosemide | Direct | 0.33 (0.03, 2.94) | 0.93 |
|  |  | Indirect | 0.29 (0.10, 0.84) |  |
| **Mannitol** | Furosemide | Direct | 0.57 (0.09, 3.53) | 0.70 |
|  |  | Indirect | 0.32 (0.03, 2.97) |  |
| **Mannitol** | Natiuretic peptides | Direct | 0.39 (0.03, 4.06) | 0.11 |
|  |  | Indirect | 4.90 (0.64, 37.70) |  |

**Appendix Table G. Between-trial heterogeneity and evaluation of model fit.**

| **Outcome** | **Heterogeneity** | **Number of data points** | **Residual deviance** |
| --- | --- | --- | --- |
| **CIAKI** | 0.29 | 318 | 334.5* |
| **Mortality** | 0.10 | 77 | 76.68 |
| **Myocardial infarction** | 0.48 | 24 | 22.9 |
| **Need for Dialysis** | 0.71 | 89 | 83.4 |
| **Heart failure** | 0.39 | 34 | 34.44 |
| **A τau^2^ estimate (between-trial variance) of 0.04 may be interpreted as low, 0.14 as moderate and 0.40 as substantial heterogeneity between trials. The model is considered to provide adequate fit to the data if the mean of the residual deviance is similar to the number of data points used in the model. *Each individual data point’s contribution to the residual deviance was assessed: the only points with high contributions were those with zero cells in one arm (eight studies). We therefore concluded that the model fit was adequate.** | | | |

**Appendix Table H. Bayesian event rates for secondary clinical outcomes.**

| ***Treatment*** | **Mortality**  **[%]** | **Myocardial infarction**  **[%]** | **Dialysis**  **[%]** | **Heart failure**  **[%]** |
| --- | --- | --- | --- | --- |
| ***Saline*** | 2.09 | 3.37 | 0.75 | 3.97 |
| ***Saline + NAC*** | 1.69 | 1.77 | 0.49 | 4.48 |
| ***Sodium Bicarbonate*** | 1.55 | 4.07 | 0.73 | 2.60 |
| ***Sodium Bicarbonate + NAC*** | 1.51 | na | 0.39 | 4.36 |
| ***Ascorbic Acid*** | 1.66 | 4.26 | 1.75 | na |
| ***Statin*** | 1.44 | 0.62 | 0.14 | 2.75 |
| ***Furosemide*** | 0.15 | na | 0.50 | 0.60 |
| ***Probucol*** | na | na | na | na |
| ***Methylxanthines*** | 0.24 | na | 0.50 | na |
| ***Fenoldopam*** | 1.39 | 6.18 | 1.00 | na |
| ***Device-guided matched hydration*** | 0.89 | 0.61 | 0.01 | na |
| ***Renal replacement therapy*** | 0.44 | 1.72 | 0.22 | 1.83 |
| ***Nebivolol*** | na | na | na | na |
| ***Natiuretic Peptides*** | na | na | 0.12 | 4.13 |
| ***Mannitol*** | na | na | na | na |
| ***Prostaglandins*** | 2.03 | na | na | na |
| ***Trimetazidine*** | na | na | na | na |
| ***LVEDP-guided hydration*** | 0.18 | 0.98 | 0.13 | na |

**Appendix Figure A. Summary Risk of bias of studies included in the network meta-analysis.**


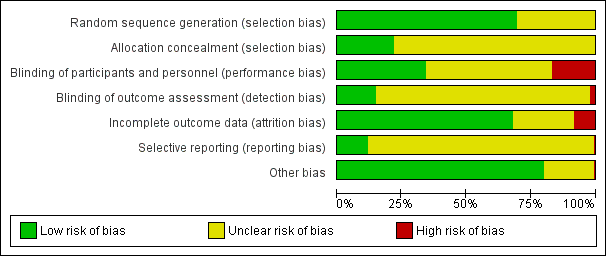


We followed the recommended approach for assessing risk of bias in studies included in Cochrane reviews. This tool addresses specific bias domains including methods for generating the random sequence, allocation concealment, blinding of participants and investigators, blinding of outcome assessment, incompleteness of outcome data, and selective outcome reporting. Each item is adjudicated within each study and the results are represented in a risk of bias table. The adjudication of the risk of bias is achieved by answering prespecified questions about the methods reported by each study in relation to the risk domain, such that the conclusion is either low risk of bias, unclear risk of bias or high risk of bias. Low risk of bias (+); unclear risk of bias (?); high risk of bias (-).

**Appendix Figure B. Funnel plots of randomised controlled trials included in the network meta-analysis for risk of: CIAKI (A), mortality (B), myocardial infarction (C), need for dialysis (D), heart failure (E). NAC – N-acetyl cysteine, OR – odds ratio, SE – standard error.**

A


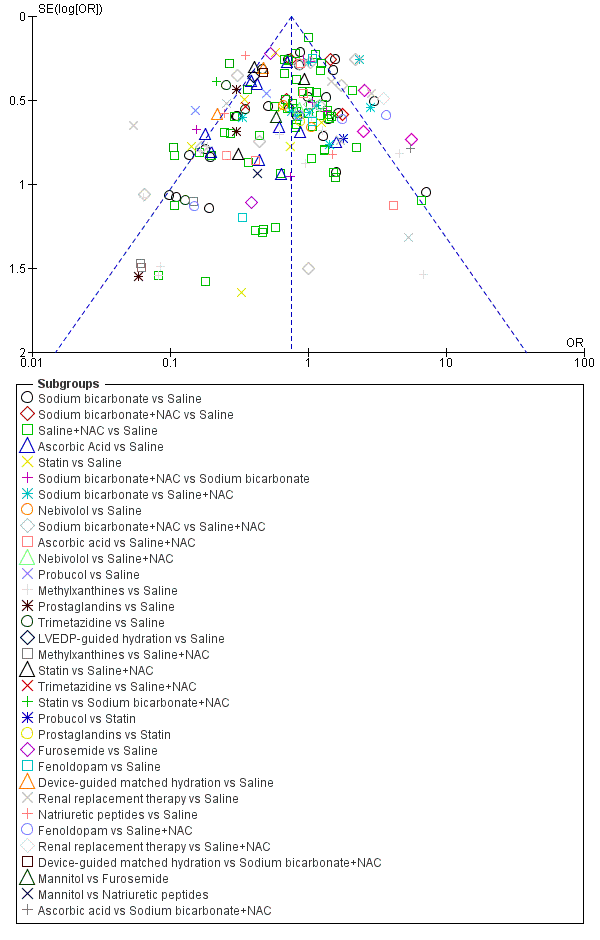


B


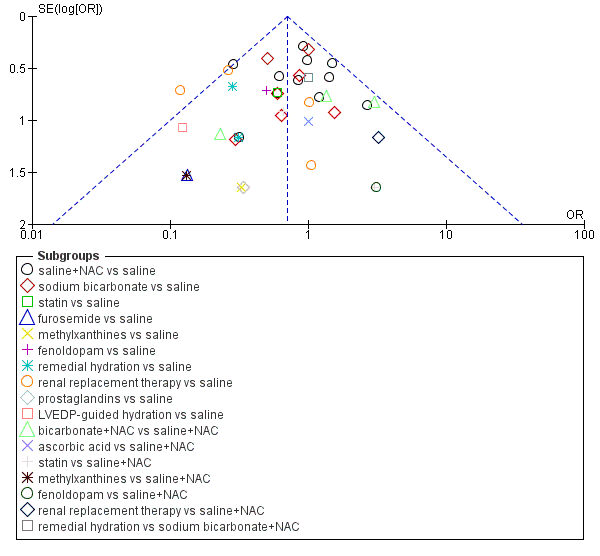


C
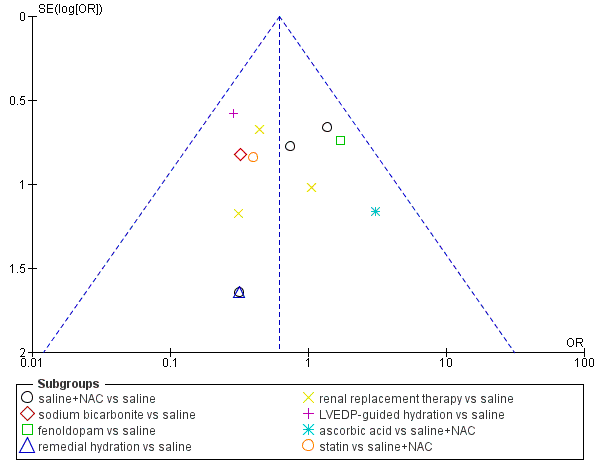


D


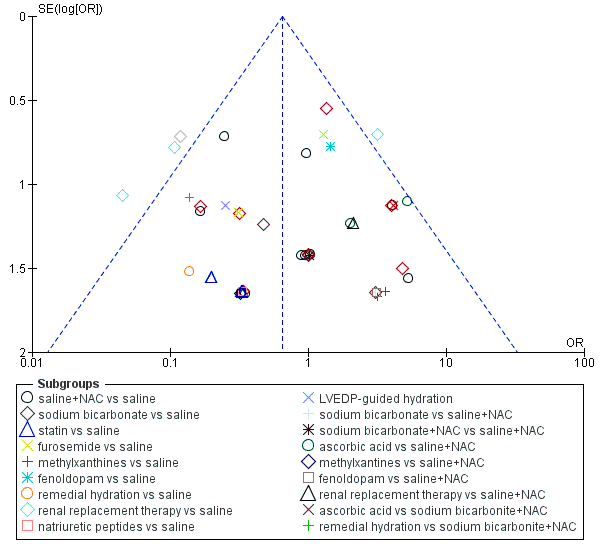


E


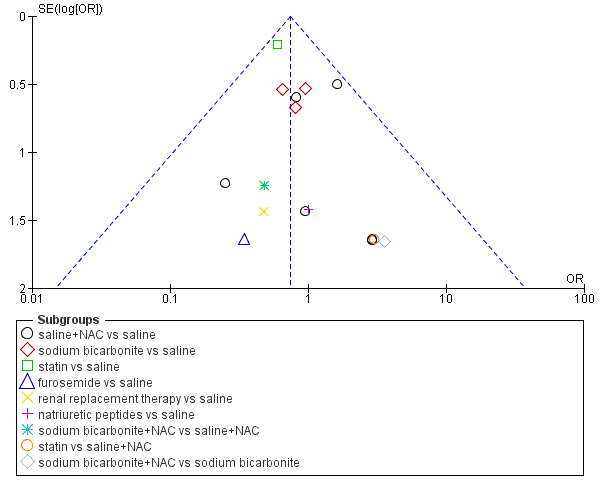


**Appendix Figure C. Rankograms of strategies to prevent contrast-induced acute kidney injury (CIAKI). The probability of achieving a given rank (x axis) in CIAKI prevention is shown for each strategy.**

**
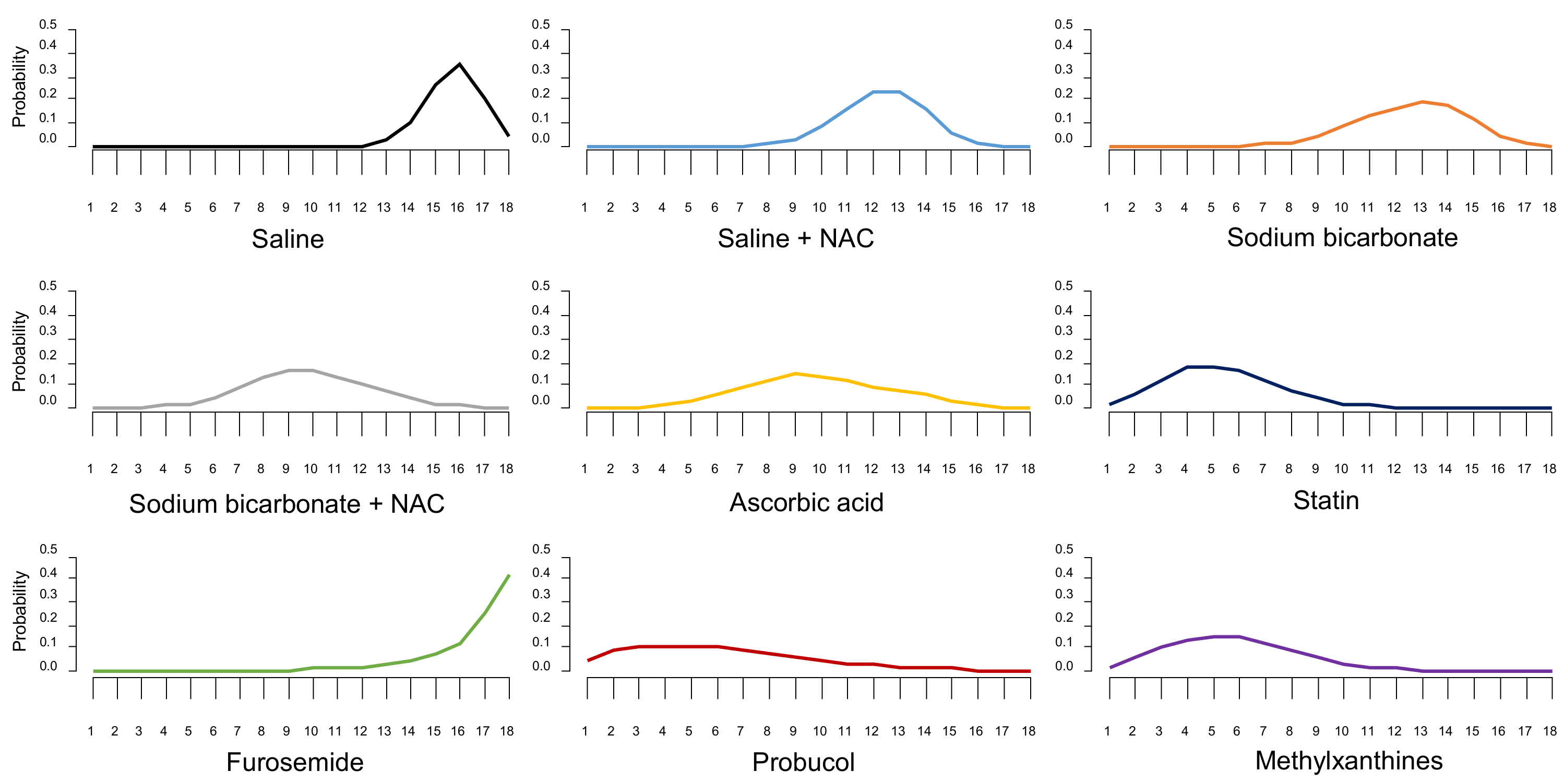
**

**
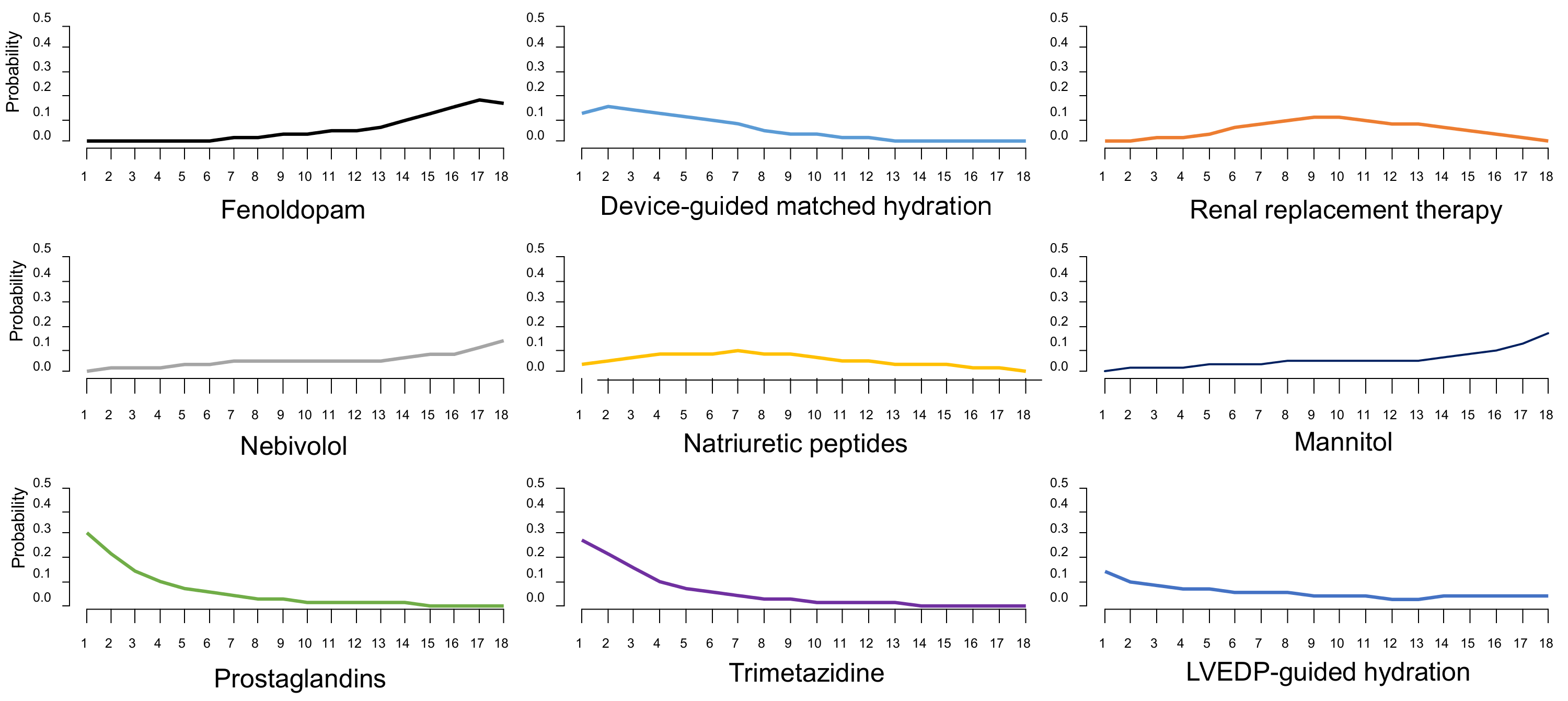
**

**Appendix Figure D. Network of treatments for secondary clinical outcomes. LVEDP, left ventricular end-diastolic pressure. NAC, N-acetyl cysteine.
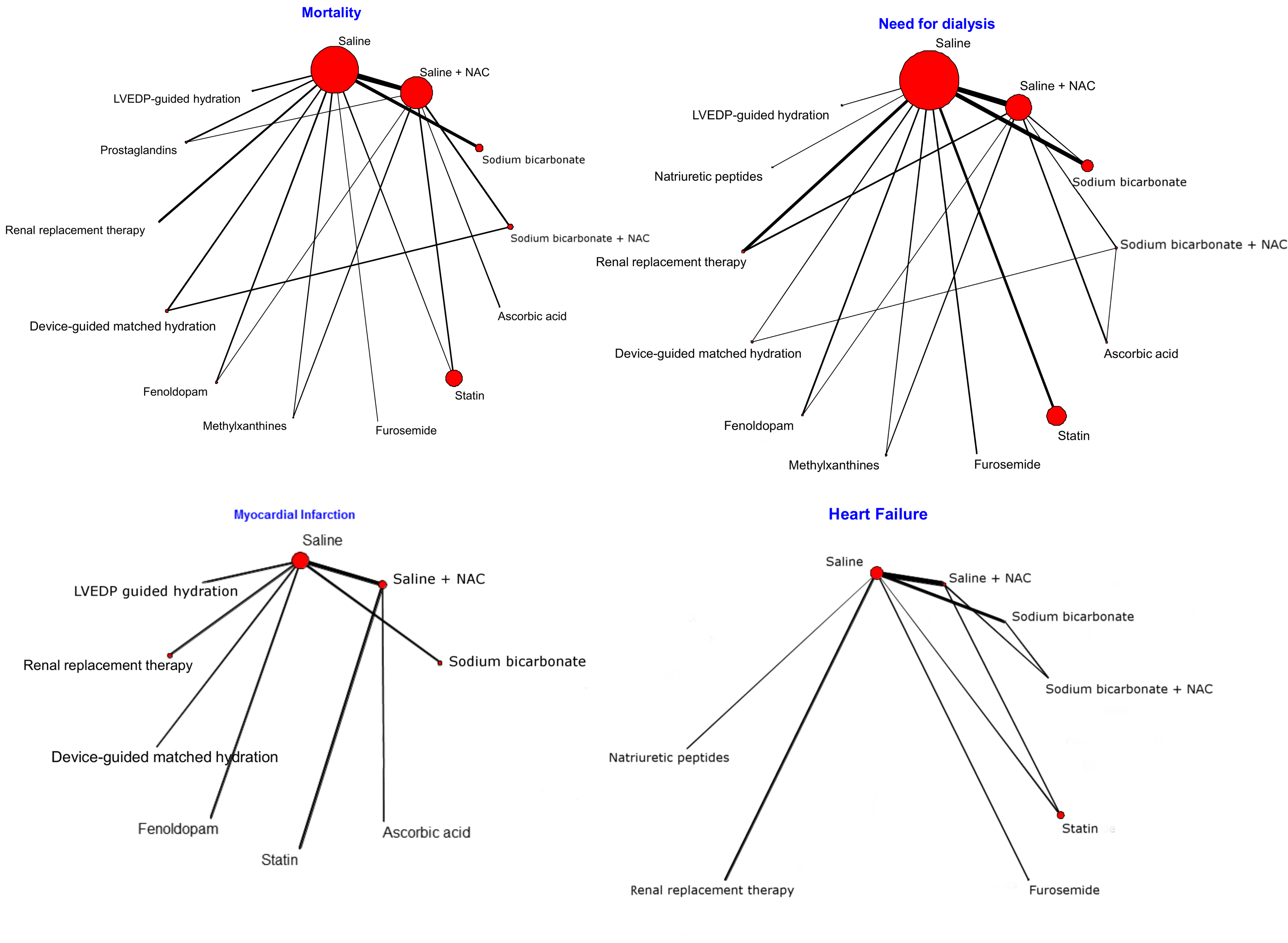
**

**Appendix Figure E.** **Pooled odds ratio and 95% credible intervals determined by network meta-analysis for contrast-induced acute kidney injury in patients with moderate chronic kidney disease. ER = event rate.**


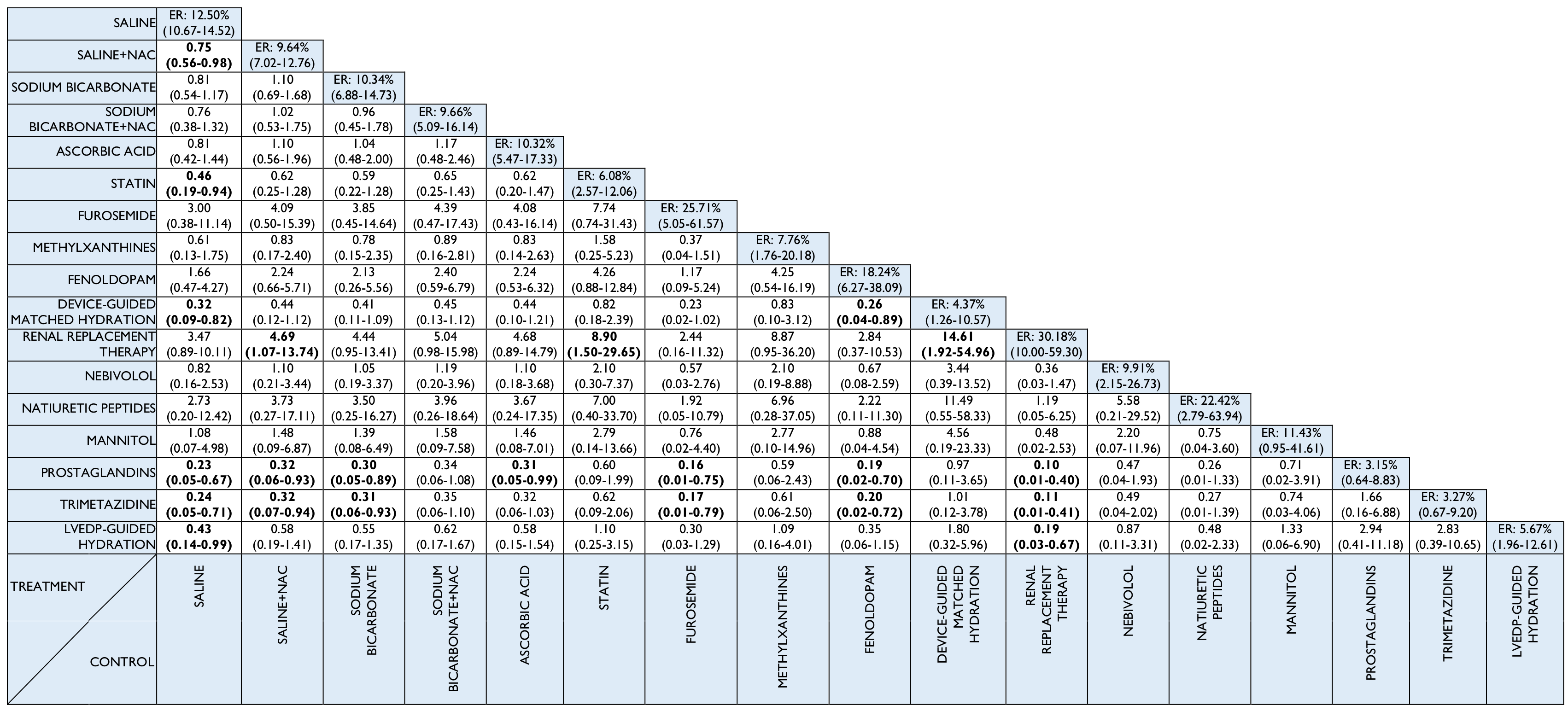


**Appendix Figure F.** **Pooled odds ratio and 95% credible intervals determined by network meta-analysis for contrast-induced acute kidney injury in patients with severe chronic kidney disease. ER = event rate.**


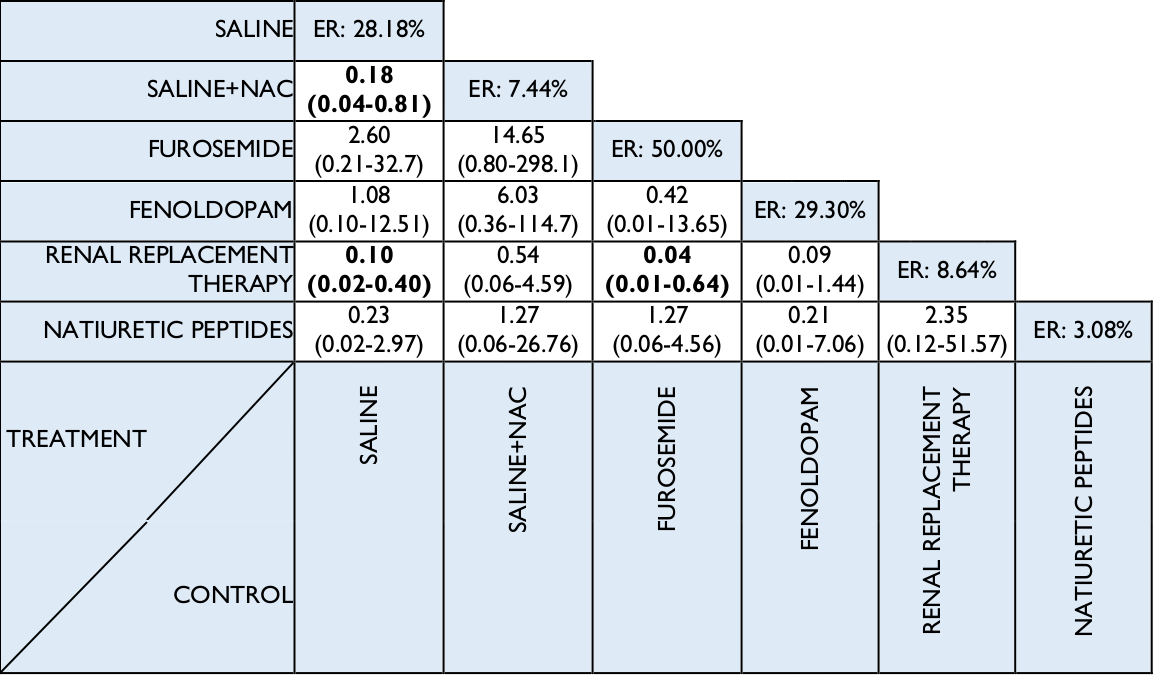


**Appendix Figure G. Pooled odds ratio and 95% credible intervals determined by network meta-analysis for contrast-induced acute kidney injury in patients receiving >100 ml of contrast medium.**


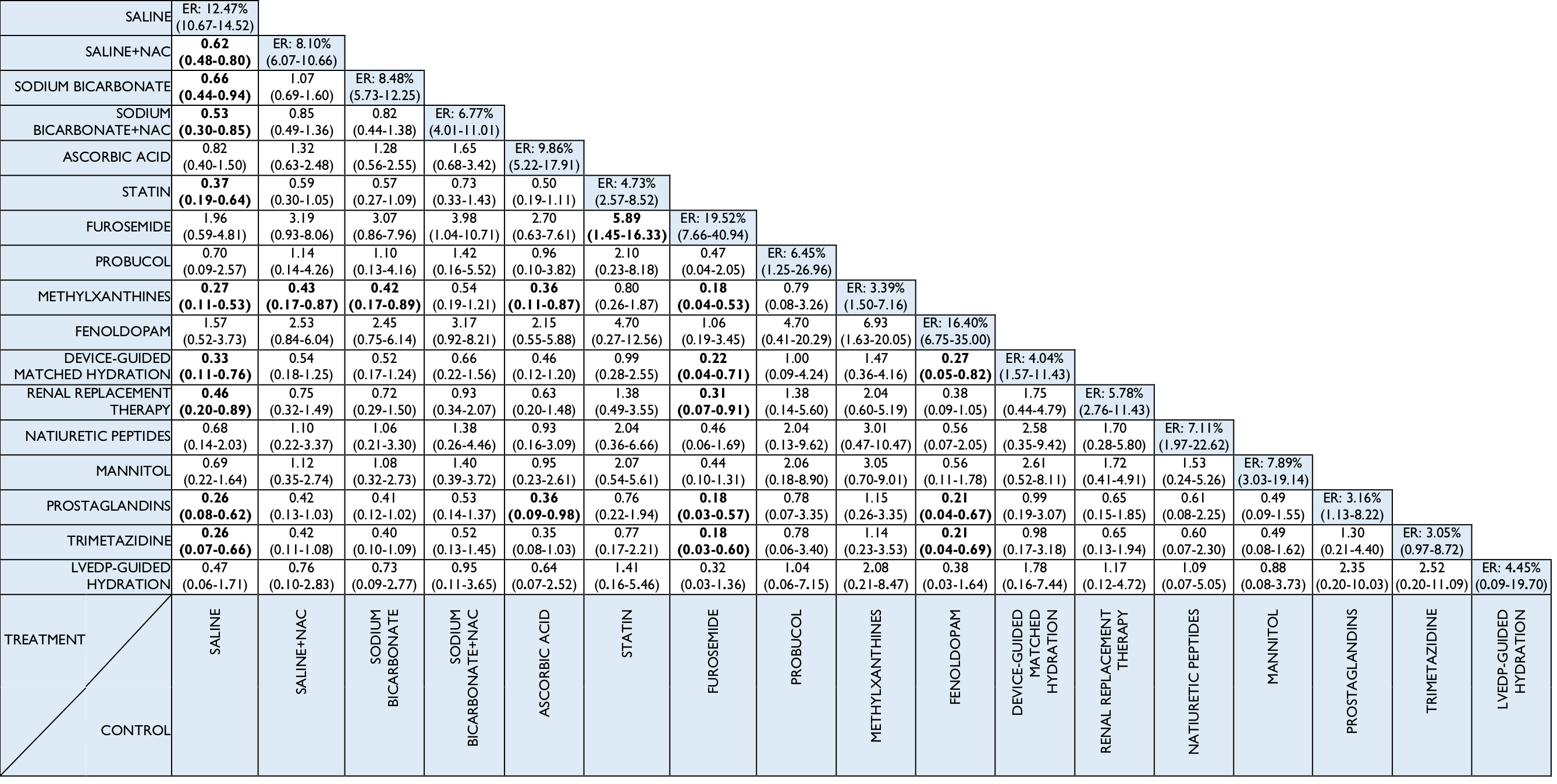


**Appendix Figure H. Pooled odds ratio and 95% credible intervals determined by network meta-analysis for contrast-induced acute kidney injury defined according to a relative (>25%) or absolute (>0.5 mg/dL or >44 umol/L) increase in serum creatinine from baseline within 48-72 hours.**


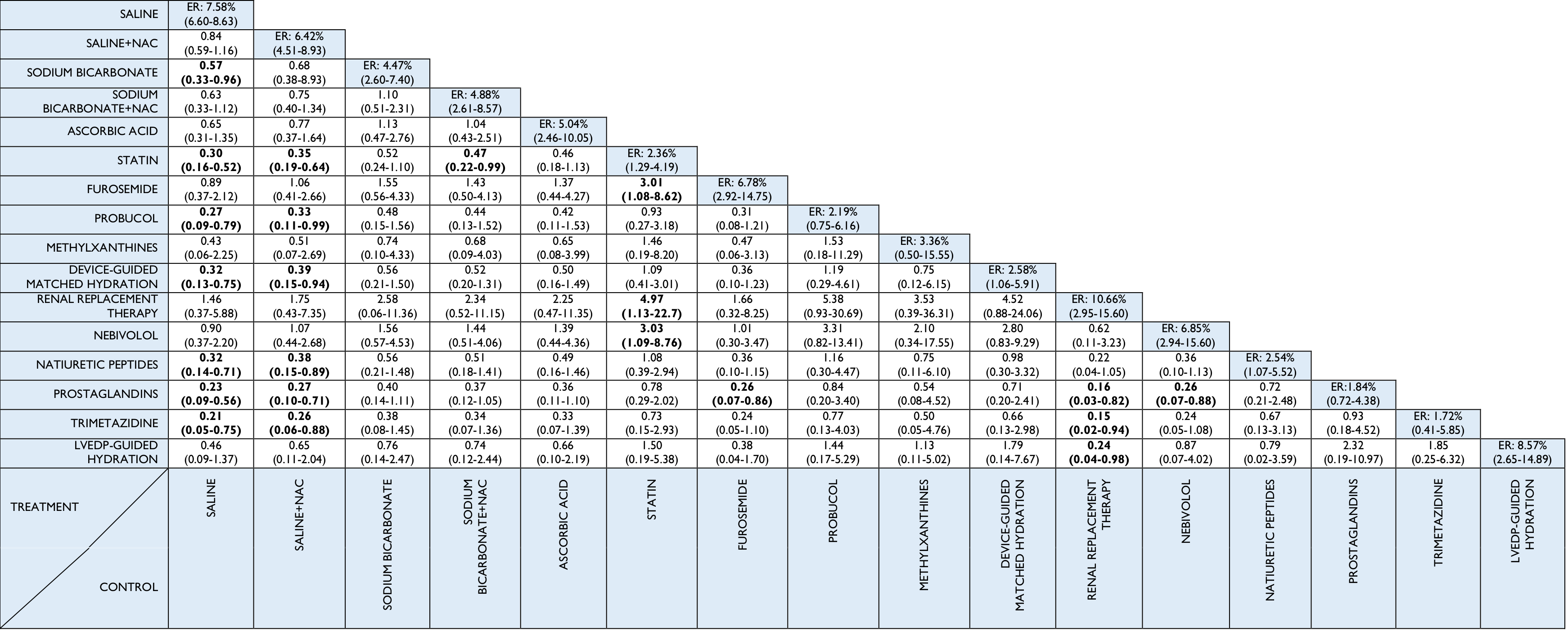


**1. Summary of current 2014 European Society of Cardiology guideline recommendations on CIAKI prevention:**

1. Patients undergoing coronary angiography or multidetector computer tomography should be assessed for risk of CIAKI. (IIa C)
2. Patients with moderate-to-severe CKD
   1. should:
      1. be hydrated with isotonic saline (I A)
      2. receive low-osmolar or iso-osmolar contrast media (I A)
      3. be considered for short-term, high-dose statin therapy (IIa A)
      4. be considered for iso-osmolar contrast media over low-osmolar contrast media (IIa A)
      5. be considered for minimised volume of contrast media (IIa B)
   2. may be considered to receive furosemide with matched hydration over standard hydration in patients at very high risk for CIN or in cases where prophylactic hydration before the procedure cannot be accomplished (IIb A)
   3. are not recommended:
      1. for N-acetyl cysteine instead of standard hydration (III A)
      2. for sodium bicarbonate 0.84% infusion
3. Patients with severe CKD:
   1. may be considered for prophylactic haemofiltration 6 hours before complex PCI (IIb B)
   2. are not recommended for prophylactic renal replacement therapy as a preventive measure (III B)

**2. Summary of current** **2011 ACCF/AHA/SCAI Guideline for Percutaneous Coronary Intervention recommendations on CIAKI prevention:**

- - - 1. Patients should be assessed for risk of contrast induced AKI before PCI. (I C)
      2. Patients undergoing cardiac catheterisation with contrast media should receive adequate preparatory hydration. (I B)
      3. In patients with CKD (creatinine clearance <60 mL/min), the volume of contrast media should be minimised. (I B)
      4. Administration of N-acetyl-L-cysteine is not useful for the prevention of contrast-induced AKI. (III A)

**3. Summary of current 2012 KDIGO Clinical Practice Guideline for Acute Kidney Injury recommendations on CIAKI prevention:**

1. Patients at risk for CI-AKI should be given the lowest possible dose of contrast medium. (Not Graded)
2. Patients at increased risk of CIAKI:
   1. should receive either iso-osmolar or low-osmolar iodinated contrast media, rather than high-osmolar iodinated contrast media (I B)
   2. should receive i.v. volume expansion with either isotonic sodium chloride or sodium bicarbonate solutions, rather than no i.v. volume expansion (I A)
   3. should not receive oral fluids alone (I C)
   4. may be considered for oral NAC, together with i.v. isotonic crystalloids (II D)
   5. should not be considered for prophylactic intermittent haemodialysis or haemofiltration for contrast-media removal (II C)
3. Theophylline use is not recommended to prevent CIAKI. (II C)
4. Fenoldopam should not be used to prevent CIAKI. (I B)

**4. Summary of 2014 KHA-CARI guideline recommendations on CIAKI prevention:**

1. Patients at increased risk of CIAKI should:

a. receive iso-osmolar or low-osmolar, rather than high-osmolar, iodinated contrast media (1B)

b. receive IV volume expansion with isotonic saline or sodium bicarbonate, rather than no IV volume expansion (1A)

c. not be considered for prophylactic intermittent haemodialysis or haemofiltration for contrast media removal. (1C)

2. Oral NAC, in addition to IV isotonic crystalloids, in patients at increased risk for CIAKI is not of proven benefit. (2A)

3. Oral route of hydration may be beneficial compared to fasting state in reducing the risk of CIAKI in patients at increased risk for CIAKI. (2C)

**Appendix references**

1. Leoncini M, Toso A, Maioli M, et al. Early high-dose rosuvastatin for contrast-induced nephropathy prevention in acute coronary syndrome: Results from the PRATO-ACS Study (Protective Effect of Rosuvastatin and Antiplatelet Therapy On contrast-induced acute kidney injury and myocardial damage in patients with Acute Coronary Syndrome). *J Am Coll Cardiol* 2014; **63**(1):71-9.

2. Marenzi G, Ferrari C, Marana I, et al. Prevention of contrast nephropathy by furosemide with matched hydration: the MYTHOS (Induced Diuresis With Matched Hydration Compared to Standard Hydration for Contrast Induced Nephropathy Prevention) trial. *JACC Cardiovasc Interv* 2012; **5**(1):90-7.

3. Shaikh F, Maddikunta R, Museitif R, et al. A prospective randomized trial comparing normal saline and sodium bicarbonate with or without N-acetyleysteine for prevention of contrast-induced nephropathy (Abstract). *Am J Cardiol* 2007; **100 [Suppl 1]**(122L-123L).

4. Thayssen, P, Lassen JF, Jensen SE, et al. Prevention of contrast-induced nephropathy with N-acetylcysteine or sodium bicarbonate in patients with ST-segment-myocardial infarction: a prospective, randomized, open-labeled trial. *Circ Cardiovasc Interv* 2014; **7**(2):216-24.

5. Ozcan EE, Guneri S, Akdeniz B, et al. Sodium bicarbonate, N-acetylcysteine, and saline for prevention of radiocontrast-induced nephropathy. A comparison of 3 regimens for protecting contrast-induced nephropathy in patients undergoing coronary procedures. A single-center prospective controlled trial. *Am Heart J* 2007; **154**(3):539-44.

6. Castini D, Lucreziotti S, Bosotti, et al. Prevention of contrast-induced nephropathy: a single center randomized study. *Clin Cardiol* 2010; **33**(3):E63-8.

7. Brueck M, Cengiz H, Hoeltgen R, et al. Usefulness of N-acetylcysteine or ascorbic acid versus placebo to prevent contrast-induced acute kidney injury in patients undergoing elective cardiac catheterization: a single-center, prospective, randomized, double-blind, placebo-controlled trial. *J Invasive Cardiol* 2013; **25**(6):276-83.

8. Albabtain MA, Almasood A, Alshurafah H, et al. Efficacy of ascorbic acid, N-acetylcysteine, or combination of both on top of saline hydration versus saline hydration alone on prevention of contrast-Induced nephropathy: a prospective randomized study. *J Interv Cardiol* 2013; **26**(1):90-6.

9. Kinbara T, Hayano T, Ohtani N, et al. Efficacy of N-acetylcysteine and aminophylline in preventing contrast-induced nephropathy*. J Cardiol* 2010; **55**(2):174-9.

10. Baskurt M, Okcun B, Abaci O, et al. N-acetylcysteine versus N-acetylcysteine + theophylline for the prevention of contrast nephropathy. *Eur J Clin Invest* 2009; **39**(9):793-9.

11. Allaqaband S Tumuluri R, Malik AM, et al. Prospective randomized study of N-acetylcysteine, fenoldopam, and saline for prevention of radiocontrast-induced nephropathy. *Catheter Cardiovasc Interv* 2002; **57**(3):279-83.

12. Loutrianakis E, Stella D, Hussain A, et al. Randomized comparison of fenoldopam and N-acetylcysteine to saline in the prevention of radio-contrast induced nephropathy. *J Am Coll Cardiol* 2003; **41**(6s1):327-327.

13. Reinecke H, Fobker M, Wellmann J, et al. A randomized controlled trial comparing hydration therapy to additional hemodialysis or N-acetylcysteine for the prevention of contrast medium-induced nephropathy: the Dialysis-versus-Diuresis (DVD) Trial. *Clin Res Cardiol* 2007; **96**(3):130-9.

14. Günebakmaz O, Kaya MG, Koc F et al. Does nebivolol prevent contrast-induced nephropathy in humans? *Clin Cardiol* 2012; **35**(4):250-4.

15. ACT Investigators. Acetylcysteine for prevention of renal outcomes in patients undergoing coronary and peripheral vascular angiography: main results from the randomized Acetylcysteine for Contrast-induced nephropathy Trial (ACT). *Circulation* 2011; **124**(11):1250-9.

16. Miner SE, Dzavik V, Nguyen-Ho P, et al. N-acetylcysteine reduces contrast-associated nephropathy but not clinical events during long-term follow-up. *Am Heart J* 2004; **148**(4):690-5.

17. Goldenberg I, Shechter M, Matetzky S, et al. Oral acetylcysteine as an adjunct to saline hydration for the prevention of contrast-induced nephropathy following coronary angiography. A randomized controlled trial and review of the current literature. *Eur Heart J* 2004; **25**(3):212-8.

18. Durham JD, Caputo C, Dokko J, et al. A randomized controlled trial of N-acetylcysteine to prevent contrast nephropathy in cardiac angiography. *Kidney Int* 2002; **62**(6):2202-7.

19. Oldemeyer JB, Biddle WP, Wurdeman RL, et al. Acetylcysteine in the prevention of contrast-induced nephropathy after coronary angiography. *Am Heart J* 2003; **146**(6):E23.

20. Baker CS, Wragg A, Kumar S, et al. A rapid protocol for the prevention of contrast-induced renal dysfunction: the RAPPID study*. J Am Coll Cardiol* 2003; **41**(12):2114-8.

21. Kefer JM, Hanet CE, Boitte S, et al. Acetylcysteine, coronary procedure and prevention of contrast-induced worsening of renal function: which benefit for which patient? *Acta Cardiol* 2003; **58**(6):555-60.

22. Efrati S, Dishy V, Averbukh M, et al. The effect of N-acetylcysteine on renal function, nitric oxide, and oxidative stress after angiography. *Kidney Int* 2003; **64**(6):2182-7.

23. Diaz-Sandoval LJ, Kosowsky BD, Losordo DW. Acetylcysteine to prevent angiography-related renal tissue injury (the APART trial). *Am J Cardiol*, 2002. **89**(3):356-8.

24. Briguori C, Manganelli F, Scarpato P, et al. Acetylcysteine and contrast agent-associated nephrotoxicity. *J Am Coll Cardiol* 2002; **40**(2):298-303.

25. Shyu KG, Cheng JJ, Kuan P. Acetylcysteine protects against acute renal damage in patients with abnormal renal function undergoing a coronary procedure. *J Am Coll Cardiol* 2002; **40**(8):1383-8.

26. Boccalandro F, Amhad M, Smalling RW, et al. Oral acetylcysteine does not protect renal function from moderate to high doses of intravenous radiographic contrast. *Catheter Cardiovasc Interv* 2003; **58**(3):336-41.

27. Kay J, Chow WH, Chan TM, et al. Acetylcysteine for prevention of acute deterioration of renal function following elective coronary angiography and intervention: a randomized controlled trial. *JAMA* 2003; **289**(5):553-8.

28. Ueda H, Yamada T, Masuda M, et al. Prevention of contrast-induced nephropathy by bolus injection of sodium bicarbonate in patients with chronic kidney disease undergoing emergent coronary procedures. *Am J Cardiol* 2011; **107**(8):1163-7.

29. Ochoa A, Pellizzon G, Addala S, et al., Abbreviated dosing of N-acetylcysteine prevents contrast-induced nephropathy after elective and urgent coronary angiography and intervention*. J Interv Cardiol* 2004; **17**(3):159-65.

30. Thiele H, Hildebrand L, Schirdewahn C, et al. Impact of high-dose N-acetylcysteine versus placebo on contrast-induced nephropathy and myocardial reperfusion injury in unselected patients with ST-segment elevation myocardial infarction undergoing primary percutaneous coronary intervention. The LIPSIA-N-ACC (Prospective, Single-Blind, Placebo-Controlled, Randomized Leipzig Immediate PercutaneouS Coronary Intervention Acute Myocardial Infarction N-ACC) Trial. *J Am Coll Cardiol* 2010; **55**(20):2201-9.

31. Webb JG, Pate GE, Humphries KH, et al. A randomized controlled trial of intravenous N-acetylcysteine for the prevention of contrast-induced nephropathy after cardiac catheterization: lack of effect. *Am Heart J* 2004; **148**(3):422-9.

32. Carbonell N, Blasco M, Sanjuán R, et al. Intravenous N-acetylcysteine for preventing contrast-induced nephropathy: a randomised trial. *Int J Cardiol* 2007; **115**(1):57-62.

33. Koc F, Ozdemir K, Kaya MG, et al. Intravenous N-acetylcysteine plus high-dose hydration versus high-dose hydration and standard hydration for the prevention of contrast-induced nephropathy: CASIS--a multicenter prospective controlled trial. *Int J Cardiol* 2012; **155**(3):418-23.

34. Amini M, Salarifar M, Amirbaigloo A, et al. N-acetylcysteine does not prevent contrast-induced nephropathy after cardiac catheterization in patients with diabetes mellitus and chronic kidney disease: a randomized clinical trial. *Trials* 2009; **10**:45.

35. Azmus AD, Gottschall C, Manica A. Effectiveness of acetylcysteine in prevention of contrast nephropathy*. J Invasive Cardiol* 2005; **17**(2):80-4.

36. Carbonell N, Sanjuán R, Blasco M, et al. N-acetylcysteine: short-term clinical benefits after coronary angiography in high-risk renal patients. *Rev Esp Cardiol* 2010; **63**(1):12-9.

37. Coyle LC, Rodriguez A, Jeschke RE, et al. Acetylcysteine In Diabetes (AID): a randomized study of acetylcysteine for the prevention of contrast nephropathy in diabetics. *Am Heart J* 2006; **151**(5):1032 e9-12.

38. Drager LF, Andrade L, Barros de Toledo JF, et al. Renal effects of N-acetylcysteine in patients at risk for contrast nephropathy: decrease in oxidant stress-mediated renal tubular injury. *Nephrol Dial Transplant* 2004; **19**(7):1803-7.

39. Mahmoodi K, Sohrabi B, Ilkhchooyi F, et al. The Efficacy of Hydration with Normal Saline Versus Hydration with Sodium Bicarbonate in the Prevention of Contrast-induced Nephropathy. *Heart Views* 2014; **15**(2):33-6.

40. Ferrario F, Barone MT, Landoni G, et al. Acetylcysteine and non-ionic isosmolar contrast-induced nephropathy--a randomized controlled study. *Nephrol Dial Transplant* 2009; **24**(10):3103-7.

41. Fung JW, Szeto CC, Chan WW, et al. Effect of N-acetylcysteine for prevention of contrast nephropathy in patients with moderate to severe renal insufficiency: a randomized trial. *Am J Kidney Dis* 2004; **43**(5):801-8.

42. Gomes VO, Poli de Figueredo CE, Caramori P, et al. N-acetylcysteine does not prevent contrast induced nephropathy after cardiac catheterisation with an ionic low osmolality contrast medium: a multicentre clinical trial. *Heart* 2005; **91**(6):774-8.

43. Gulel O, Keles T, Eraslan H, et al. Prophylactic acetylcysteine usage for prevention of contrast nephropathy after coronary angiography. *J Cardiovasc Pharmacol* 2005; **46**(4):464-7.

44. Kim BJ, Sung KC, Kim BS, et al. Effect of N-acetylcysteine on cystatin C-based renal function after elective coronary angiography (ENABLE Study): a prospective, randomized trial. *Int J Cardiol* 2010; **138**(3):239-45.

45. Kimmel M, Butscheid M, Brenner S, et al. Improved estimation of glomerular filtration rate by serum cystatin C in preventing contrast induced nephropathy by N-acetylcysteine or zinc--preliminary results. *Nephrol Dial Transplant* 2008; **23**(4):1241-5.

46. Kotlyar E, Keogh AM, Thavapalachandran S, et al. Prehydration alone is sufficient to prevent contrast-induced nephropathy after day-only angiography procedures--a randomised controlled trial. *Heart Lung Circ* 2005; **14**(4):245-51.

47. MacNeill BD, Harding SA, Bazari H, et al. Prophylaxis of contrast-induced nephropathy in patients undergoing coronary angiography. *Catheter Cardiovasc Interv* 2003; **60**(4):458-61.

48. Marenzi G, Assanelli E, Marana I, et al. N-acetylcysteine and contrast-induced nephropathy in primary angioplasty. *N Engl J Med* 2006; **354**(26):2773-82.

49. Namgung J, Doh JH, Lee SY, et al. Effect of N-Acetylcysteine in Prevention of Contrast-Induced Nephropathy after Coronary Angiography *Korean Circ J* 2005; **35**(9):696-701.

50. Seyon RA, Jensen LA, Ferguson IA, et al. Efficacy of N-acetylcysteine and hydration versus placebo and hydration in decreasing contrast-induced renal dysfunction in patients undergoing coronary angiography with or without concomitant percutaneous coronary intervention. *Heart Lung* 2007; **36**(3):195-204.

51. Sinha SK, Berry WAD, Bueti J, et al. The Prevention of Radiocontrast-Induced Nephropathy Trial (PRINT): A Prospective, Double-Blind, Randomized, Controlled Trial of Iso-Osmolar Versus Low-Osmolar Radiocontrast in Combination with N-Acetylcysteine Versus Placebo (Abstract). *Circulation* 2004; **110 (suppl 5):377**.

52. Erturk M, Uslu N, Gorgulu S, et al. Does intravenous or oral high-dose N-acetylcysteine in addition to saline prevent contrast-induced nephropathy assessed by cystatin C? *Coron Artery Dis* 2014; **25**(2):111-7.

53. Merten GJ, Burgess WP, Gray LV, et al. Prevention of contrast-induced nephropathy with sodium bicarbonate: a randomized controlled trial. *JAMA* 2004; **291**(19):2328-34.

54. Boucek P, Havrdova T, Oliyarnyk O, et al. Prevention of contrast-induced nephropathy in diabetic patients with impaired renal function: a randomized, double blind trial of sodium bicarbonate versus sodium chloride-based hydration. *Diabetes Res Clin Pract* 2013; **101**(3):303-8.

55. Masuda M, Yamada T, Mine T, et al. Comparison of usefulness of sodium bicarbonate versus sodium chloride to prevent contrast-induced nephropathy in patients undergoing an emergent coronary procedure*. Am J Cardiol* 2007; **100**(5):781-6.

56. Adolph E, Holdt-Lehmann B, Chatterjee T, et al. Renal Insufficiency Following Radiocontrast Exposure Trial (REINFORCE): a randomized comparison of sodium bicarbonate versus sodium chloride hydration for the prevention of contrast-induced nephropathy. *Coron Artery Dis* 2008; **19**(6):413-9.

57. Pakfetrat M, Nikoo MH, Malekmakan L,et al. A comparison of sodium bicarbonate infusion versus normal saline infusion and its combination with oral acetazolamide for prevention of contrast-induced nephropathy: a randomized, double-blind trial. *Int Urol Nephrol* 2009; **41**(3):629-34.

58. Hengel C, Chase A, Klinke W et al. Bicarbonate for coronary angiographic renal protection (BICAR) trial [abstract] in Paper presented at Canadian Cardiovascular Congress: Vancouver, Canada 2006.

59. Zhou L, Chen H, Prevention of contrast-induced nephropathy with ascorbic acid. *Intern Med* 2012; **51**(6):531-5.

60. Tamura A, Miyamoto, K, Naono S, et al. A single bolus intravenous administration of sodium bicarbonate is effective in the prevention of contrast-induced nephropathy in patients with renal insufficiency undergoing diagnostic coronary arteriography or elective percutaneous coronary intervention. *Circulation* 2008; **118**(18 Supplement):S_658.

61. Vasheghani-Farahani A, Sadigh G, Kassaian SE, et al. Sodium bicarbonate in preventing contrast nephropathy in patients at risk for volume overload: a randomized controlled trial. *J Nephrol* 2010; **23**(2):216-23.

62. Vasheghani-Farahani A, Sadigh G, Kassaian SE, et al. Sodium bicarbonate plus isotonic saline versus saline for prevention of contrast-induced nephropathy in patients undergoing coronary angiography: a randomized controlled trial. *Am J Kidney Dis* 2009; **54**(4):610-8.

63. Motohiro M, Kamihata H, Tsujimoto S, et al. A new protocol using sodium bicarbonate for the prevention of contrast-induced nephropathy in patients undergoing coronary angiography. *Am J Cardiol* 2011; **107**(11):1604-8.

64. Klima T, Christ A, Marana I, et al. Sodium chloride vs. sodium bicarbonate for the prevention of contrast medium-induced nephropathy: a randomized controlled trial*. Eur Heart J* 2012; **33**(16):2071-9.

65. Maioli M, Toso A, Leoncini M, et al. Effects of hydration in contrast-induced acute kidney injury after primary angioplasty: a randomized, controlled trial*. Circ Cardiovasc Interv* 2011; **4**(5):456-62.

66. Manari A, Magnavacchi P, Puggioni E, et al. Acute kidney injury after primary angioplasty: effect of different hydration treatments*. J Cardiovasc Med (Hagerstown)* 2014; **15**(1):60-7.

67. Spargias K, Alexopoulos E, Kyrzopoulos S, et al. Ascorbic acid prevents contrast-mediated nephropathy in patients with renal dysfunction undergoing coronary angiography or intervention. *Circulation* 2004; **110**(18):2837-42.

68. Boscheri A, Weinbrenner C, Botzek B, et al. Failure of ascorbic acid to prevent contrast-media induced nephropathy in patients with renal dysfunction. *Clin Nephrol* 2007; **68**(5):279-86.

69. Komiyama K., Tejima T, Tanabe Y, et al. Impact of Bolus Administration of Ascorbic Acid to Prevent Contrast Induced Nephropathy Undergoing Emergent Cardiovascular Procedure. *Am J Cardiol* 2011; **107**(8):29A.

70. Li R, Chen H. Prevention of contrast-induced nephropathy with ascorbic acid. *Heart* 2012; **98**(Suppl 2):E211-E211.

71. Hamdi S, Selmi W, Hraiech A, et al. Prevention Of Contrast Induced Nephropathy In Patients Undergoing Coronarography With Ascorbic Acid. *JACC: Cardiovascular Interventions* 2013; **6**(2_S):S22-S22.

72. Han Y, Zhu G, Han L, et al. Short-term rosuvastatin therapy for prevention of contrast-induced acute kidney injury in patients with diabetes and chronic kidney disease. *J Am Coll Cardiol* 2014; **63**(1):62-70.

73. Patti G, Ricottini E, Nusca A, et al. Short-term, high-dose Atorvastatin pretreatment to prevent contrast-induced nephropathy in patients with acute coronary syndromes undergoing percutaneous coronary intervention (from the ARMYDA-CIN [atorvastatin for reduction of myocardial damage during angioplasty--contrast-induced nephropathy] trial. *Am J Cardiol* 2011; **108**(1):1-7.

74. Acikel S, Muderrisoglu H, Yildirir A, et al. Prevention of contrast-induced impairment of renal function by short-term or long-term statin therapy in patients undergoing elective coronary angiography. *Blood Coagul Fibrinolysis* 2010; **21**(8):750-7.

75. Jo SH, Koo BK, Park JS, et al. Prevention of radiocontrast medium-induced nephropathy using short-term high-dose simvastatin in patients with renal insufficiency undergoing coronary angiography (PROMISS) trial--a randomized controlled study. *Am Heart J* 2008; **155**(3):499 e1-8.

76. Oliveira MS, Martins KBA, Costa JR, et al., Impact on Renal Function of Rosuvastatin Preload Prior to Elective Percutaneous Coronary Intervention in Chronic Statin Users. *Revista Brasileira de Cardiologia Invasiva English Version* 2012;**20**(3):303-308.

77. Li W, Fu X, Wang Y et al. Beneficial effects of high-dose atorvastatin pretreatment on renal function in patients with acute ST-segment elevation myocardial infarction undergoing emergency percutaneous coronary intervention. *Cardiology* 2012; **122**(3):195-202.

78. Solomon R, Werner C, Mann D, et al. Effects of saline, mannitol, and furosemide on acute decreases in renal function induced by radiocontrast agents. *N Engl J Med* 1994; **331**(21):1416-1420.

79. Majumdar SR, Kjellstrand CM, Tymchak WJ, et al Forced euvolemic diuresis with mannitol and furosemide for prevention of contrast-induced nephropathy in patients with CKD undergoing coronary angiography: a randomized controlled trial. *Am J Kidney Dis* 2009; **54**(4):602-9.

80. Gu G, Zhang Y, Lu R, et al. Low-dose furosemide administered with adequate hydration reduces contrast-induced nephropathy in patients undergoing coronary angiography. *Cardiology* 2013; **125**(2):69-73.

81. Yin L, Li G, Liu T, et al. Probucol for the prevention of cystatin C-based contrast-induced acute kidney injury following primary or urgent angioplasty: a randomized, controlled trial. *Int J Cardiol* 2013; **167**(2):426-9.

82. Li G, Yin L, Liu T, et al. Role of probucol in preventing contrast-induced acute kidney injury after coronary interventional procedure. *Am J Cardiol* 2009; **103**(4):512-4.

83. Abizaid AS, Clark CE, Mintz GS, et al. Effects of dopamine and aminophylline on contrast-induced acute renal failure after coronary angioplasty in patients with preexisting renal insufficiency. *Am J Cardiol* 1999; **83**(2):260-3, A5.

84. Rohani A. Effectiveness of aminophylline prophylaxis of renal impairment after coronary angiography in patients with chronic renal insufficiency. *Indian J Nephrol* 2010; **20**(2):80-3.

85. Kapoor A, Kumar S, Gulati S, Gambhir S, Sethi RS, Sinha N. The role of theophylline in contrast-induced nephropathy: a case-control study. *Nephrol Dial Transplant* 2002; **17**(11):1936-41.

86. Huber W, Schipek C, Ilgmann K, et al. Effectiveness of theophylline prophylaxis of renal impairment after coronary angiography in patients with chronic renal insufficiency. *Am J Cardiol* 2003; **91**(10):1157-62.

87. Matejka J, Varvarovsky I, Vojtisek P, et al. Prevention of contrast-induced acute kidney injury by theophylline in elderly patients with chronic kidney disease. *Heart Vessels* 2010; **25**(6):536-42.

88. Stone GW, McCullough PA, Tumlin JA, et al. Fenoldopam mesylate for the prevention of contrast-induced nephropathy: a randomized controlled trial. *JAMA* 2003; **290**(17):2284-91.

89. Lee PT, Chou KJ, Liu CP, et al. Renal protection for coronary angiography in advanced renal failure patients by prophylactic hemodialysis. A randomized controlled trial. *J Am Coll Cardiol* 2007; **50**(11):1015-20.

90. Vogt B, Ferrari P, Schönholzer C, et al. Prophylactic hemodialysis after radiocontrast media in patients with renal insufficiency is potentially harmful. *Am J Med* 2001; **111**(9):692-8.

91. Marenzi G, Marana I, Lauri G, et al. The prevention of radiocontrast-agent-induced nephropathy by hemofiltration. *N Engl J Med* 2003; **349**(14):1333-40.

92. Marenzi G, Lauri G, Campodonico J, et al. Comparison of two hemofiltration protocols for prevention of contrast-induced nephropathy in high-risk patients. *Am J Med* 2006; **119**(2):155-162.

93. Lehnert T, Keller E, Gondolf K, et al. Effect of haemodialysis after contrast medium administration in patients with renal insufficiency. *Nephrol Dial Transplant* 1998; **13**(2):358-62.

94. Berger ED, Bader BD, Bosker J, et al. [Contrast media-induced kidney failure cannot be prevented by hemodialysis]. *Deutsche medizinische Wochenschrift* (1946) 2001; **126**(7):162-166.

95. Weisberg LS, Kurnik PB, Kurnik BR. Risk of radiocontrast nephropathy in patients with and without diabetes mellitus. *Kidney Int* 1994; **45**(1):259-65.

96. Morikawa S, Sone T, Tsuboi H, et al. Renal protective effects and the prevention of contrast-induced nephropathy by atrial natriuretic peptide. *J Am Coll Cardiol* 2009; **53**(12):1040-6.

97. Spargias K, Adreanides E, Demerouti E, et al. Iloprost prevents contrast-induced nephropathy in patients with renal dysfunction undergoing coronary angiography or intervention. *Circulation* 2009; **120**(18):1793-9.

98. Spargias K, Adreanides E, Giamouzis G, et al. Iloprost for prevention of contrast-mediated nephropathy in high-risk patients undergoing a coronary procedure. Results of a randomized pilot study. *Eur J Clin Pharmacol* 2006; **62**(8):589-95.

99. Li WH, Li DY, Qian WH, et al. Prevention of contrast-induced nephropathy with prostaglandin E1 in high-risk patients undergoing percutaneous coronary intervention. *Int Urol Nephrol* 2014; **46**(4):781-6.

100. Briguori C, Airoldi F, D'Andrea D, et al. Renal Insufficiency Following Contrast Media Administration Trial (REMEDIAL): a randomized comparison of 3 preventive strategies. *Circulation* 2007; **115**(10):1211-7.

101. Recio-Mayoral A, Chaparro M, Prado B, et al. The reno-protective effect of hydration with sodium bicarbonate plus N-acetylcysteine in patients undergoing emergency percutaneous coronary intervention: the RENO Study. *J Am Coll Cardiol* 2007; **49**(12):1283-8.

102. Maioli M, Toso A, Leoncini M, et al. Sodium bicarbonate versus saline for the prevention of contrast-induced nephropathy in patients with renal dysfunction undergoing coronary angiography or intervention. *J Am Coll Cardiol* 2008; **52**(8):599-604.

103. Heguilen R, Liste A, Gabriela R. Prevention of contrast-induced nephropathy: Volume expansion, N-acetylcysteine or both? Results from a pilot study. *Nephrol Dial Transplant* 2007; **22:vi 54, (suppl 6; abstr)**.

104. Lee SW, Kim WJ, Kim YH, et al. Preventive strategies of renal insufficiency in patients with diabetes undergoing intervention or arteriography (the PREVENT Trial). *Am J Cardiol* 2011; **107**(10):1447-52.

105. Jo SH, Koo BK, Park JS, et al. N-acetylcysteine versus AScorbic acid for preventing contrast-Induced nephropathy in patients with renal insufficiency undergoing coronary angiography NASPI study-a prospective randomized controlled trial. *Am Heart J* 2009; **157**(3):576-83.

106. Toso A, Maioli M, Leoncini M, et al. Usefulness of atorvastatin (80 mg) in prevention of contrast-induced nephropathy in patients with chronic renal disease. *Am J Cardiol* 2010; **105**(3):288-92.

107. Ozhan H, Erden I, Ordu S, et al. Efficacy of short-term high-dose atorvastatin for prevention of contrast-induced nephropathy in patients undergoing coronary angiography. *Angiology* 2010; **61**(7):11-4.

108. Bilasy ME, Oraby MA, Ismail HM, et al. Effectiveness of theophylline in preventing contrast-induced nephropathy after coronary angiographic procedures. *J Interv Cardiol* 2012; **25**(4):404-10.

109. Heng AE, Cellarier E, Aublet-Cuvelier B, et al. Is treatment with N-acetylcysteine to prevent contrast-induced nephropathy when using bicarbonate hydration out of date? *Clin Nephrol* 2008; **70**(6):475-84.

110. Quintavalle C, Fiore D, De Micco F, et al. Impact of a high loading dose of atorvastatin on contrast-induced acute kidney injury. *Circulation* 2012; **126**(25):3008-16.

111. Briguori C, Visconti G, Focaccio A, et al. Renal Insufficiency After Contrast Media Administration Trial II (REMEDIAL II): RenalGuard System in high-risk patients for contrast-induced acute kidney injury. *Circulation* 2011; **124**(11):1260-9.

112. Han S, Li XM, Mohammed Ali LA, et al .Effect of short-term different statins loading dose on renal function and CI-AKI incidence in patients undergoing invasive coronary procedures. *Int J Cardiol* 2013; **168**(5):5101-3.

113. Liu WJ, Zhang BC, Guo R, et al. Renoprotective effect of alprostadil in combination with statins in patients with mild to moderate renal failure undergoing coronary angiography. *Chin Med J (Engl)* 2013; **126**(18):3475-80.

114. Luo Y, Wang X, Ye Z, et al. Remedial hydration reduces the incidence of contrast-induced nephropathy and short-term adverse events in patients with ST-segment elevation myocardial infarction: a single-center, randomized trial. Intern Med 2014; **53**(20):2265-72.

115. Onbasili AO, Yeniceriglu Y, Agaoglu P, et al. Trimetazidine in the prevention of contrast-induced nephropathy after coronary procedures. *Heart* 2007; **93**(6):698-702.

116. Shehata M. Impact of trimetazidine on incidence of myocardial injury and contrast-induced nephropathy in diabetic patients with renal dysfunction undergoing elective percutaneous coronary intervention. *Am J Cardiol* 2014; **114**(3):389-94.

117. Yeganehkhah MR, Iranirad L, Dorri F, et al. Comparison between three supportive treatments for prevention of contrast-induced nephropathy in high-risk patients undergoing coronary angiography. *Saudi J Kidney Dis Transpl* 2014; **25**(6):1217-23.

118. Grygier M, Janus M, Araszkiewicz A, et al. Combined treatment with ascorbic acid and N-acetylcysteine prevents contrast-induced nephropathy in high-risk patients with acute myocardial infarction undergoing percutaneous coronary intervention. *Eur Heart J (Abstract Supplement)* 2011; **32**: p. 954-955.

119. Akgüllü Ç, Eryılmaz U, Güngör H, et al. A clinical study about contrast nephropathy: risk factors and the role of beta blockers. *Anatol J Cardiol* 2015;15(3):232-40.

120. Yang K, Liu W, Ren W, et al. Different interventions in preventing contrast-induced nephropathy after percutaneous coronary intervention. *Int Urol Nephrol* 2014; **46**(9):1801-7.

121. Liu JM, Xie YN, Gao ZH, et al. Brain natriuretic peptide for prevention of contrast-induced nephropathy after percutaneous coronary intervention or coronary angiography. *Can J Cardiol* 2014; **30**(12):1607-12.

122. Dvoršak B, Kanič V, Ekart R, et al. Ascorbic Acid for the prevention of contrast-induced nephropathy after coronary angiography in patients with chronic renal impairment: a randomized controlled trial. *Ther Apher Dial.* 2013;**17**(4):384-90.

123. El Mahmoud R, Le Feuvre C, Le Quan Sang KH, et al. [Absence of nephro-protective effect of acetylcysteine in patients with chronic renal failure investigated by coronary angiography]. *Arch Mal Coeur Vaiss* 2003; **96**(12):1157-61.

124. Brar SS. MEENA (A Randomized Controlled Trial for the Prevention of Contrast-Induced Nephropathy with Sodium Bicarbonate in Persons Undergoing Coronary Angiography). *Clin. Cardiol* 2007. **30**(8)**:** p.416.

125. Malhis M, Al-Bitar S, Al-Deen Zaiat K. The Role of Theophylline in Prevention of Radiocontrast Media-Induced Nephropathy. *Saudi* *J Kidney Dis Transpl* 2010; **21**(2): p.276-283.

126. Dussol B, Morange S, Loundoun A, et al. A randomized trial of saline hydration to prevent contrast nephropathy in chronic renal failure patients. *Nephrol Dial Transplant* 2006; **21**(8):2120-2126.

127. Shavit L, Korenfeld R, Lifschitz M, et al. Sodium Bicarbonate versus Sodium Chloride and Oral N-Acetylcysteine for the Prevention of Contrast-Induced Nephropathy in Advanced Chronic Kidney Disease. *J Interv Cardiol.* 2009;**22**(6):556-63.

128. Huber W, Ilgmann K, Page M, et al. Effect of Theophylline on Contrast Material–induced Nephropathy in Patients with Chronic Renal Insufficiency: Controlled, Randomized, Double-blinded Study. Radiology 2002; **223**(3):772-9.

129. Rashid ST, Salman M, Myint F, et al. Prevention of contrast-induced nephropathy in vascular patients undergoing angiography: A randomized controlled trial of intravenous N-acetylcysteine. *J Vasc Surg* 2004; **40**(6):1136-41.

130. Shemirani H, Pourrmoghaddas M. A Randomized Trial of Saline Hydration to Prevent Contrast-Induced Nephropathy in Patients on Regular Captopril or Furosemide Therapy Undergoing Percutaneous Coronary Intervention. *Saudi J Kidney Dis Transpl* 2012. **23**(2): 280-5.

131. Brar SS, Aharonian V, Mansukhani P, et al. Haemodynamic-guided fluid administration for the prevention of contrast-induced acute kidney injury: the POSEIDON randomised controlled trial. *Lancet* 2014; **383**(9931):1814-23.

132. Briguori C, Colombo A, Airoldi F, et al. N-Acetylcysteine versus fenoldopam mesylate to prevent contrast agent-associated nephrotoxicity. *J Am Coll Cardiol* 2004; **44**(4):762-5.

133. Solomon R, Gordon P, Manoukian SV, et al. Randomized Trial of Bicarbonate or Saline Study for the Prevention of Contrast-Induced Nephropathy in Patients with CKD. *Clin J Am Soc Nephrol* 2015; **10**(9):1519-24.

134. Droppa M, Desch S, Blase P, et al. Impact of N-acetylcysteine on contrast-induced nephropathy defined by cystatin C in patients with ST-elevation myocardial infarction undergoing primary angioplasty. *Clin Res Cardiol* 2011; **100**(11):1037-43.

135. Abaci O, Arat Ozkan A, Kocas C, et al. Impact of Rosuvastatin on contrast-induced acute kidney injury in patients at high risk for nephropathy undergoing elective angiography. *Am J Cardiol.* 2015; **115**(7):867-71.

136. Balderramo DC, Verdu MB, Ramacciotti CF, et al. Renoprotective effect of high periprocedural doses of oral N-acetylcysteine in patients scheduled to undergo a same-day angiography. *Rev Fac Cien Med Univ Nac Cordoba*. 2004. **61**(2):13-9.

137. Koc F, Ozdemir K, Altunkas F, et al. Sodium bicarbonate versus isotonic saline for the prevention of contrast-induced nephropathy in patients with diabetes mellitus undergoing coronary angiography and/or intervention: a multicenter prospective randomized study. *J Investig* Med 2013; **61**(5):872-7.

138. Aslanger E, Uslu B, Akdeniz C, et al. Intrarenal application of N-acetylcysteine for the prevention of contrast medium-induced nephropathy in primary angioplasty. *Coron Artery Dis* 2012; **3**(4):265-70.

139. Gomes VO, Lasevitch R, Lima VC, et al. Hydration with sodium bicarbonate does not prevent contrast nephropathy: a multicenter clinical trial. *Arq Bras Cardiol* 2012; **99**(6):1129-34.

140. Heguilén RM, Liste AA, Payaslian M, et al. N-acethyl-cysteine reduces the occurrence of contrast-induced acute kidney injury in patients with renal dysfunction: a single-center randomized controlled trial. *Clin Exp Nephrol* 2013; **17**(3):396-404.

141. Inda-Filho AJ, Caixeta A, Manggini M, et al. Do intravenous N-acetylcysteine and sodium bicarbonate prevent high osmolal contrast-induced acute kidney injury? A randomized controlled trial. *PLoS One* 2014; **9**(9):e107602.

142. Liu W, Ming Q, Shen J, et al. Trimetazidine Prevention of Contrast-Induced Nephropathy in Coronary Angiography. *Am J Med Sci* 2015; **350**(5):398-402.

143. Rahman MM, Haque SS, Rokeya B, et al. Trimetazidine in the prevention of contrast induced nephropathy after coronary angiogram. *Mymensingh Med J* 2012; **21**(2):292-9.

144. Tanaka A, Suzuki Y, Suzuki N, et al. Does N-acetylcysteine reduce the incidence of contrast-induced nephropathy and clinical events in patients undergoing primary angioplasty for acute myocardial infarction? *Intern Med* 2011; **50**(7):673-7.

145. Ng TM, Shurmur SW, Silver M, et al. Comparison of N-acetylcysteine and fenoldopam for preventing contrast-induced nephropathy (CAFCIN). *Int J Cardiol* 2006; **109**(3):322-8.

146. Huber W, Eckel F, Hennig M, et al. Prophylaxis of contrast material-induced nephropathy in patients in intensive care: acetylcysteine, theophylline, or both? A randomized study. *Radiology* 2006; **239**(3):793-804.

147. Yavari V, Ostovan MA, Kojuri J, et al. The preventive effect of pentoxifylline on contrast-induced nephropathy: a randomized clinical trial. *Int Urol Nephrol* 2014; **46**(1):41-6.
